# Evolution of the global burden of vascular intestinal diseases in middle-aged and elderly population: Trend analysis from 1990 to 2021 and projections to 2040

**DOI:** 10.1097/MD.0000000000046774

**Published:** 2025-12-26

**Authors:** Zengkai Zhou, Tong Zhang, Ruo Shu, Huayou Luo

**Affiliations:** a Department of Gastrointestinal Surgery, The First Affiliated Hospital of Kunming Medical University, Yunnan, China.

**Keywords:** global burden of disease, middle-aged and elderly population, projection, trend analysis, vascular intestinal diseases

## Abstract

Vascular intestinal diseases (VID), including mesenteric ischemia and ischemic colitis, are increasingly important public health issues, particularly among aging populations. However, their long-term epidemiological trends and future global burden remain underexplored. This observational cross-sectional study aimed to assess global VID trends from 1990 to 2021 and project future burden through 2040 using data from the Global Burden of Disease 2021 (GBD 2021) database. We extracted VID-related incidence, prevalence, and disability-adjusted life years (DALYs) data for individuals aged ≥45 years from GBD 2021. Age-standardized rates (ASRs) and estimated annual percentage change were used to quantify trends. Joinpoint regression, age-period-cohort analysis, decomposition analysis, and health inequality indices were applied to evaluate patterns and contributing factors. The Bayesian age-period-cohort model was used to project VID burden to 2040. From 1990 to 2021, global ASRs for VID showed a consistent decline: –0.49%/yr for incidence, –0.51% for prevalence, and –1.48% for DALYs. Despite this, the absolute number of VID cases and DALYs increased, driven primarily by population growth and aging. Age-period-cohort analysis revealed that VID risk rose with age but declined across successive birth cohorts. Decomposition analysis identified demographic changes as the main drivers of the increased burden. Health inequality metrics showed a narrowing of global disparities over time. Projections indicate continued increases in total VID cases by 2040, although ASRs are expected to decline further. The global burden of VID in middle-aged and elderly populations reflects both progress in disease control and emerging demographic challenges. While individual risk is decreasing, population aging continues to drive up the absolute burden. Efforts should prioritize early intervention, equitable healthcare access, and targeted public health strategies, particularly in low- and middle-sociodemographic index regions.

## 1. Introduction

Vascular intestinal diseases (VID), encompassing mesenteric ischemia, ischemic colitis, and intestinal angiodysplasia, represent life-threatening conditions arising from compromised intestinal blood supply.^[1]^ With a global incidence of 8.11/1,00,000 person-years and mortality rate of 1.26/1,00,000 person-years, VID poses a substantial public health challenge, particularly as mortality escalates dramatically with age – from approximately 1% to 3% in younger adults to nearly 50% in individuals aged 95 years and older.^[1]^ These disorders frequently necessitate emergency surgical intervention for complications including intestinal obstruction, perforation, and bowel necrosis, with delayed diagnosis often resulting in catastrophic outcomes.^[2]^ The clinical presentation of VID ranges from acute abdominal pain and gastrointestinal bleeding to chronic symptoms such as postprandial pain and weight loss, yet the insidious nature of many cases contributes to significant diagnostic delays.^[3]^ Current management strategies have evolved from predominantly open surgical approaches to increasingly sophisticated endovascular interventions, though overall mortality rates remain concerningly high, particularly for acute mesenteric ischemia.^[3]^

Previous investigations have begun to illuminate the global burden of VID, though substantial gaps remain in our understanding of age-specific trends and future projections. Jiang and colleagues analyzed VID burden using Global Burden of Disease (GBD) 2019 data, reporting that global prevalent cases increased from 1,00,158 in 1990 to 1,75,740 in 2019, while age-standardized prevalence rates (ASPRs) declined from 2.47 to 2.21/1,00,000 population over the same period.^[4]^ Their analysis identified high and high-middle sociodemographic index (SDI) regions as bearing the greatest disease burden.^[4]^ More recently, Zhang et al extended this analysis through 2021 using Bayesian age-period-cohort (BAPC) modeling, projecting that global VID incidence would rise from 1.09/1,00,000 in 2021 to 1.48/1,00,000 by 2044.^[2]^ Regional analyses have further revealed marked geographic disparities: in the United States, despite an overall decline in age-adjusted mortality rates from 9.35/1,00,000 in 1999 to 5.81/1,00,000 in 2020, certain demographic groups – including women, Native Americans, and residents of rural Midwest regions – continue to experience disproportionately high mortality burdens.^[1]^ Within aging societies such as Japan, where VID constitutes a growing component of gastrointestinal disease burden, projections indicate continued increases in crude disease rates through 2035, driven primarily by demographic shifts.^[5]^

Despite these advances in understanding VID epidemiology, critical knowledge gaps persist that limit effective public health planning and resource allocation. First, existing studies have predominantly employed all-age analyses, failing to adequately capture the distinctive burden experienced by middle-aged and elderly populations who constitute the primary at-risk demographic. The arbitrary yet clinically meaningful threshold of 45 years – representing the transition to middle age and coinciding with accelerated vascular aging and increased comorbidity accumulation – has not been systematically examined as a specific population segment.^[6]^ Second, while recent studies have extended analyses through 2021, none have specifically leveraged the GBD 2021 dataset, which uniquely incorporates pandemic-era data and represents the most comprehensive and methodologically rigorous burden estimation to date.^[2]^ Third, projection models extending beyond the next 2 decades remain scarce, limiting our capacity to anticipate healthcare system demands as global population aging accelerates through mid-century. Finally, the interplay between sociodemographic development and VID burden evolution remains inadequately characterized, particularly regarding whether improvements in healthcare infrastructure and economic development translate into meaningful reductions in disease burden across diverse settings.^[1,4]^

This study addresses these knowledge gaps by providing the first comprehensive assessment of VID burden specifically focused on middle-aged and elderly populations (aged ≥ 45 years) from 1990 to 2021, with projections extending to 2040. Utilizing the GBD 2021 database, we systematically analyzed temporal trends in VID incidence and disability-adjusted life years (DALYs) across 204 countries and territories, stratified by sex, age group, and SDI quintile. We employed joinpoint regression analysis to identify critical inflection points in disease trends and utilized BAPC modeling to generate evidence-based projections through 2040. By specifically targeting the middle-aged and elderly population – the demographic cohort bearing the greatest VID burden and projected to expand most rapidly in coming decades – this analysis aims to inform targeted prevention strategies, optimize healthcare resource allocation, and guide policy development for an aging global population. Our findings will provide essential epidemiological evidence for clinicians, public health officials, and policymakers seeking to mitigate the growing impact of VID in an increasingly aged world.

## 2. Materials and methods

### 2.1. Data source

This study utilized data from the GBD 2021 database (https://ghdx.healthdata.org/gbd-2021), which provides comprehensive estimates for 204 countries and territories, 371 diseases and injuries, and 88 risk factors. We extracted data on incidence, prevalence, and DALYs for VID in the middle-aged and elderly population (aged 45 years and above) from 1990 to 2021.^[7–10]^ Analyses were stratified by sex, SDI quintiles, 21 GBD regions, and 204 countries and all indicators were obtained from GBD 2021, which uses standardized modeling to address data gaps; no additional imputation was performed. The selection of 45 years as the lower age limit for middle-aged and elderly populations was based on 3 considerations: epidemiological evidence demonstrating accelerated VID mortality escalation beginning in this age group^[1]^; physiological factors, as 45 years represents the transition to middle age with accelerated vascular aging and comorbidity accumulation^[6]^; and demographic relevance, as this population segment is projected to expand most rapidly due to global aging trends, making it the most policy-relevant target for VID prevention and management strategies.

### 2.2. Ethics approval and consent to participate

This study is based solely on de-identified, aggregated data from the GBD 2021 database, which is publicly available and does not include information on individual participants. As such, ethical approval and informed consent were not required.

### 2.3. Disease definition

According to the GBD 2021 classification, VID is categorized under non-communicable diseases, specifically within the digestive diseases category. In the International Classification of Diseases 10th revision (ICD-10), VID is primarily coded under K55, while in ICD-11, it is classified under DD10.^[8,9]^

### 2.4. Estimated annual percentage change (EAPC)

The EAPC was calculated to quantify the long-term trends in age-standardized rates (ASRs) of VID burden. A linear regression model was applied to the natural logarithm of ASR against calendar year to estimate EAPC (95% confidence interval [CI]). The EAPC and its 95% CI were derived from the regression coefficient and its standard error.

### 2.5. Joinpoint regression analysis

Joinpoint regression was used to identify significant changes in trends over time. This method determines the points where a statistically significant change in the linear slope of the trend occurs. We calculated the annual percent change for each identified trend segment and the average annual percent change (AAPC) for the entire study period.

### 2.6. Age-period-cohort (APC) analysis

We constructed an age-period-cohort (APC) model to disentangle the effects of age, period, and birth cohort on VID burden. The model was fitted using a generalized linear model framework, with age, period, and cohort as independent variables and the disease burden measure as the dependent variable.

### 2.7. Health inequality analysis

To assess health inequalities, we calculated the slope index of inequality (SII) and the concentration index. The SII measures absolute inequality across socioeconomic groups, while the CI quantifies relative inequality, ranging from −1 to 1, with 0 indicating perfect equality.

### 2.8. Decomposition analysis

We performed decomposition analysis to attribute changes in VID burden to factors such as population growth, population aging, and epidemiological changes.

### 2.9. Sociodemographic index (SDI) analysis

We examined the relationship between VID burden and the SDI, a composite measure of development status. LOESS smoothing was applied to visualize trends, and Spearman’s correlation analysis was conducted to quantify the relationship between SDI and disease burden indicators. SDI stratification was based on the standard 5-level classification provided by GBD 2021 (low, low-middle, middle, high-middle, and high SDI). All primary analyses were conducted within these predefined SDI strata to examine burden heterogeneity across socioeconomic development levels.

### 2.10. Bayesian age-period-cohort (BAPC) projection model

We employed the BAPC model to project future trends in VID burden from 2022 to 2040. This Bayesian framework incorporates age, period, and cohort effects to generate predictions of disease rates. To assess the predictive performance of the BAPC model, we conducted posterior predictive checks by fitting the model to data from 1990 to 2014 and comparing predicted values for 2015 to 2021 with observed data. Model adequacy was evaluated using the mean absolute percentage error and coverage probability of 95% credible intervals.

### 2.11. Statistical software

All data processing, statistical analyses, and visualizations were performed using R version 4.4.1. Specific packages used included “Joinpoint,” “apc,” “INLA,” and custom scripts for inequality and decomposition analyses.

## 3. Results

### 3.1. Global burden of vascular intestinal diseases in middle-aged and elderly population: a 32-year analysis

**Figure 1.**
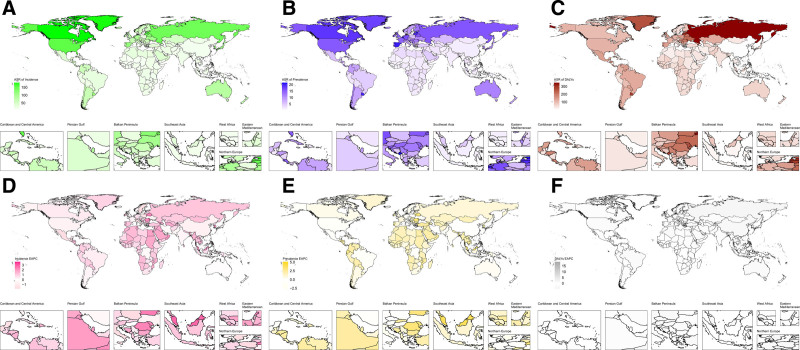
Global burden of vascular intestinal diseases (VID) in middle-aged and elderly population: incidence, prevalence, and DALYs in 2021 and their changes from 1990 to 2021. (A) Age-standardized incidence rate of VID globally in 2021; (B) estimated annual percentage change (EAPC) in age-standardized incidence rate of VID from 1990 to 2021; (C) age-standardized prevalence rate of VID globally in 2021; (D) estimated annual percentage change (EAPC) in age-standardized prevalence rate of VID from 1990 to 2021; (E) age-standardized DALY rate of VID globally in 2021; and (F) estimated annual percentage change (EAPC) in age-standardized DALY rate of VID from 1990 to 2021. ASR = age-standardized rate, DALY = disability-adjusted life year, EAPC = estimated annual percentage change, SDI = sociodemographic index, UI = uncertainty interval, VID = vascular intestinal diseases.

The global burden of VID among middle-aged and elderly individuals has exhibited notable trends over the past 32 years. Although the absolute number of cases has increased, the ASR has shown a consistent decline. Specifically, the global incidence of VID rose from 5,89,892.93 cases (95% uncertainty interval [UI]: 4,14,410.57–7,95,964.83) → 11,35,437.63 cases (95% UI: 8,43,821.17–14,74,539.68) in 2021. Despite this increase, the ASR decreased from 57.71 (41.01–77.08) → 48.56 (36.20–62.87), with an EAPC of −0.49% (−0.58 to −0.41; Table 1). Figure 1A, B illustrates the global age-standardized incidence rate of VID in 2021 and its EAPC from 1990 to 2021. Similarly, the prevalence followed a comparable pattern, rising from 72,442.92 cases (95% UI: 57,358.68–90,979.54) in 1990 to 1,42,531.85 cases (95% UI: 1,16,326.93–1,73,250.93) in 2021, while the ASR declined from 7.07/1,00,000 (95% UI: 5.65–8.80) to 6.11 (95% UI: 5.00–7.40), with an EAPC of –0.51% (95% CI: –0.62% to –0.41%; Table 2). Figure 1C, D presents the global ASPR of VID in 2021 and the corresponding EAPC from 1990 to 2021. Notably, the change in DALYs was even more pronounced, increasing from 10,12,673.52 (95% UI: 9,26,459.09–11,16,136.38) → 15,42,022.95 (95% UI: 13,93,487.43–16,68,706.96). However, the ASR for DALYs declined substantially from 103.78 (94.16–114.22) → 66.82 (60.12–72.39), with an EAPC −1.48% (−1.59 to −1.36; Table 3). Figure 1E, F shows the global age-standardized DALY rate of VID in 2021 and its trend over the past 3 decades. Together, the data presented in Figure 1 provide a comprehensive overview of the burden of VID in 2021 and its changes since 1990 across incidence, prevalence, and DALYs.

**Table 1:** Global and regional incidence of middle-aged and elderly VID in 1990 and 2021, and EAPC of ASR from 1990 to 2021.

Location	1990		2021		EAPC, 1990–2021
	Number	ASR	Number	ASR	
Global	5,89,892.93 (4,14,410.57–7,95,964.83)	57.71 (41.01–77.08)	11,35,437.63 (8,43,821.17–14,74,539.68)	48.56 (36.20–62.87)	−0.49 (−0.58 to −0.41)
SDI quintiles					
High SDI	3,40,372.73 (2,41,921.62–4,53,987.43)	111.68 (78.76–150.19)	6,00,002.79 (4,53,314.44–7,65,794.92)	104.48 (77.76–135.36)	−0.15 (−0.25 to −0.05)
High-middle SDI	1,61,158.29 (1,12,830.98–2,18,297.04)	61.04 (43.13–82.16)	2,87,874.57 (2,14,687.49–3,72,196.39)	52.57 (39.24–67.94)	−0.4 (−0.45 to −0.34)
Middle SDI	53,302.70 (34,937.54–76,665.82)	20.58 (13.91–28.91)	1,49,487.20 (1,02,630.02–2,06,960.09)	20.71 (14.42–28.35)	0.04 (0–0.08)
Low-middle SDI	26,790.49 (17,472.20–38,609.17)	17.32 (11.64–24.43)	77,448.43 (53,376.85–1,06,955.18)	20.69 (14.51–28.17)	0.66 (0.61–0.7)
Low SDI	7846.99 (5118.27–11,362.23)	14.09 (9.47–19.97)	19,805.94 (13,604.68–27,779.57)	15.73 (11.11–21.56)	0.36 (0.34–0.38)
SDI regions					
Andean Latin America	955.19 (646.38–1331.77)	18.04 (12.42–24.81)	3591.89 (2487.53–4985.62)	22.65 (15.77–31.29)	0.79 (0.71–0.88)
Australasia	5722.13 (3866.17–7998.59)	87.50 (58.89–122.86)	12,586.81 (8393.42–18,014.62)	82.50 (54.46–119.07)	−0.08 (−0.12 to −0.04)
Caribbean	2013.35 (1348.12–2877.41)	28.92 (19.47–41.17)	4926.72 (3398.12–6883.37)	33.22 (22.91–46.40)	0.51 (0.47–0.55)
Central Asia	3179.17 (2092.61–4554.29)	25.37 (16.89–36.00)	7174.03 (4905.02–9907.51)	33.68 (23.58–45.69)	1.18 (1.06–1.3)
Central Europe	13,966.61 (9756.75–19,355.55)	34.54 (24.18–47.80)	25,924.73 (19,613.30–34,353.61)	42.15 (31.45–56.46)	0.36 (0.15–0.58)
Central Latin America	11,462.05 (7975.93–15,686.50)	53.84 (38.08–72.57)	32,569.69 (22,760.88–44,415.39)	48.59 (34.19–65.90)	−0.41 (−0.47 to −0.36)
Central Sub-Saharan Africa	1113.89 (726.34–1603.82)	21.48 (14.49–30.30)	2950.78 (1968.09–4248.42)	22.18 (15.27–31.13)	0.07 (−0.12 to 0.26)
East Asia	39,178.66 (23,133.34–59,987.86)	17.48 (10.66–26.18)	98,948.24 (65,523.28–1,42,344.60)	16.20 (10.84–23.14)	−0.38 (−0.53 to −0.24)
Eastern Europe	86,897.41 (61,425.77–1,15,821.21)	113.60 (80.56–151.03)	1,32,005.26 (98,763.82–1,68,214.39)	134.05 (100.09–171.20)	0.8 (0.69–0.91)
Eastern Sub-Saharan Africa	2216.80 (1471.17–3186.99)	13.01 (8.90–18.32)	6443.46 (4416.12–9070.91)	16.47 (11.64–22.66)	0.86 (0.81–0.92)
High-income Asia Pacific	57,172.74 (36,530.82–83,190.90)	102.20 (65.68–147.90)	1,29,292.85 (90,430.28–1,74,585.12)	110.22 (74.06–154.48)	0.52 (0.35–0.69)
High-income North America	1,78,259.03 (1,29,624.10–2,30,465.49)	182.92 (131.56–239.17)	2,85,549.95 (2,20,724.96–3,53,204.92)	156.87 (120.30–195.92)	−0.54 (−0.68 to −0.41)
North Africa and Middle East	8535.83 (5389.15–12,583.62)	19.47 (12.64–28.07)	35,694.41 (23,435.24–51,123.89)	29.90 (20.17–41.82)	1.28 (1.2–1.36)
Oceania	76.82 (46.29–117.10)	9.70 (6.13–14.34)	230.95 (147.84–337.26)	11.23 (7.48–15.87)	0.4 (0.34–0.46)
South Asia	26,428.02 (16,999.94–38,229.47)	18.27 (12.16–25.77)	76,629.55 (52,783.52–1,05,879.39)	19.92 (13.95–27.15)	0.29 (0.23–0.36)
Southeast Asia	8310.33 (5248.52–12,236.95)	13.42 (8.78–19.28)	28,974.27 (18,986.91–41,329.74)	17.56 (11.82–24.57)	0.89 (0.88–0.91)
Southern Latin America	8222.68 (5644.57–11,211.04)	65.65 (45.17–89.41)	17,293.78 (12,562.36–23,118.60)	71.75 (51.88–96.28)	0.23 (0.13–0.32)
Southern Sub-Saharan Africa	1221.64 (771.71–1792.01)	16.90 (10.92–24.38)	3020.35 (2061.29–4210.42)	19.66 (13.73–26.86)	0.56 (0.46–0.66)
Tropical Latin America	7549.56 (5665.78–10,032.47)	31.98 (24.35–42.02)	20,245.22 (14,671.18–27,492.24)	28.82 (20.99–39.01)	0.39 (0.04–0.74)
Western Europe	1,24,665.88 (86,472.80–1,73,768.76)	76.80 (52.66–108.13)	2,03,786.75 (1,49,395.55–2,70,520.92)	78.53 (56.32–105.94)	0.25 (0.12–0.38)
Western Sub-Saharan Africa	2745.13 (1719.17–4087.84)	12.28 (7.91–17.94)	7597.93 (4943.89–10,944.14)	15.44 (10.38–21.66)	0.8 (0.73–0.88)

ASR = age-standardized rate (per 1,00,000 population), CI = confidence interval, EAPC = estimated annual percentage change, SDI = sociodemographic index, VID = vascular intestinal diseases.

**Table 2:** Global and regional prevalence of middle-aged and elderly VID in 1990 and 2021, and EAPC of ASR from 1990 to 2021.

Location	1990		2021		EAPC, 1990–2021
	Number	ASR	Number	ASR	
Global	72,442.92 (57,358.68–90,979.54)	7.07 (5.65–8.80)	1,42,531.85 (1,16,326.93–1,73,250.93)	6.11 (5.00–7.40)	−0.51 (−0.62 to −0.41)
SDI quintiles					
High SDI	41,651.60 (33,357.77–51,536.97)	13.60 (10.77–16.99)	74,509.67 (61,610.54–89,269.76)	12.89 (10.47–15.69)	−0.24 (−0.35 to −0.14)
High-middle SDI	20,985.23 (16,791.54–26,042.65)	7.92 (6.36–9.79)	39,356.50 (32,739.96–47,043.60)	7.20 (5.99–8.61)	−0.3 (−0.43 to −0.17)
Middle SDI	6367.22 (4570.54–8675.99)	2.38 (1.75–3.17)	18,363.61 (13,974.39–23,854.02)	2.53 (1.95–3.25)	0.28 (0.24–0.33)
Low-middle SDI	2606.25 (1786.18–3647.14)	1.62 (1.14–2.22)	8029.11 (5980.12–10,512.14)	2.10 (1.59–2.72)	0.93 (0.87–0.98)
Low SDI	764.50 (520.88–1069.62)	1.30 (0.91–1.79)	2121.14 (1562.28–2792.96)	1.61 (1.22–2.09)	0.71 (0.66–0.76)
SDI regions					
Andean Latin America	92.07 (65.62–125.61)	1.71 (1.24–2.30)	501.83 (379.60–652.11)	3.17 (2.41–4.09)	2.17 (2.11–2.23)
Australasia	714.46 (565.76–888.20)	10.89 (8.55–13.65)	1721.91 (1378.58–2129.99)	11.20 (8.81–14.07)	−0.03 (−0.09 to 0.04)
Caribbean	279.45 (214.02–359.13)	3.98 (3.05–5.10)	796.16 (631.72–991.59)	5.38 (4.27–6.69)	0.96 (0.92–1)
Central Asia	343.91 (251.05–464.86)	2.72 (2.01–3.64)	834.65 (642.34–1071.54)	3.93 (3.09–4.94)	1.52 (1.37–1.66)
Central Europe	2406.04 (1909.17–2999.73)	5.86 (4.64–7.32)	5535.52 (4628.85–6563.90)	8.91 (7.37–10.69)	1.13 (0.87–1.39)
Central Latin America	1233.63 (969.85–1553.25)	5.69 (4.55–7.08)	4636.08 (3759.03–5644.58)	6.90 (5.62–8.36)	0.6 (0.57–0.63)
Central Sub-Saharan Africa	122.54 (86.09–166.39)	2.18 (1.56–2.93)	364.68 (274.37–471.03)	2.58 (1.98–3.28)	0.46 (0.18–0.73)
East Asia	4319.84 (2694.89–6517.22)	1.91 (1.24–2.81)	11,653.72 (8280.27–16,139.64)	1.91 (1.38–2.63)	0.07 (−0.01 to 0.15)
Eastern Europe	9707.34 (7934.86–11,810.92)	12.58 (10.25–15.33)	14,518.60 (12,470.08–16,743.67)	14.78 (12.64–17.12)	0.68 (0.56–0.8)
Eastern Sub-Saharan Africa	243.39 (169.10–333.46)	1.33 (0.94–1.80)	821.49 (618.71–1065.56)	1.96 (1.51–2.50)	1.33 (1.28–1.39)
High-income Asia Pacific	6030.39 (4087.58–8500.80)	10.77 (7.36–15.09)	13,084.21 (9822.01–17,023.14)	11.17 (7.93–15.19)	0.36 (0.19–0.52)
High-income North America	18,773.83 (15,397.91–22,760.61)	19.32 (15.63–23.73)	28,529.34 (23,975.30–33,768.99)	15.75 (13.08–18.84)	−0.76 (−0.85 to −0.67)
North Africa and Middle East	857.69 (564.19–1238.17)	1.92 (1.30–2.70)	3902.65 (2777.98–5343.51)	3.28 (2.42–4.36)	1.78 (1.73–1.83)
Oceania	7.63 (4.67–11.55)	0.93 (0.60–1.37)	23.48 (15.90–33.44)	1.12 (0.80–1.54)	0.5 (0.43–0.56)
South Asia	2417.36 (1615.92–3430.45)	1.60 (1.10–2.22)	7176.48 (5239.78–9562.39)	1.82 (1.35–2.40)	0.45 (0.38–0.51)
Southeast Asia	828.17 (544.74–1205.42)	1.31 (0.90–1.84)	3281.71 (2371.20–4432.21)	2.01 (1.50–2.64)	1.42 (1.39–1.45)
Southern Latin America	942.54 (755.77–1161.16)	7.47 (5.99–9.21)	2485.77 (2056.20–2967.92)	10.26 (8.45–12.31)	1.01 (0.94–1.09)
Southern Sub-Saharan Africa	117.33 (76.36–170.08)	1.59 (1.06–2.27)	292.33 (211.99–393.58)	1.86 (1.38–2.46)	0.62 (0.49–0.75)
Tropical Latin America	1604.35 (1288.48–1965.74)	6.47 (5.24–7.87)	3267.73 (2640.17–3987.26)	4.62 (3.74–5.63)	−0.73 (−0.87 to −0.58)
Western Europe	21,128.67 (17,138.08–25,734.80)	12.79 (10.24–15.78)	38,309.03 (32,056.32–45,480.20)	14.39 (11.83–17.38)	0.24 (0.08–0.4)
Western Sub-Saharan Africa	272.29 (175.06–397.81)	1.18 (0.78–1.69)	794.48 (561.25–1090.58)	1.57 (1.14–2.09)	0.96 (0.89–1.04)

ASR = age-standardized rate (per 1,00,000 population), CI = confidence interval, EAPC = estimated annual percentage change, SDI = sociodemographic index, VID = vascular intestinal diseases.

**Table 3:** Global and regional DALYs of middle-aged and elderly VID in 1990 and 2021, and EAPC of ASR from 1990 to 2021.

Location	1990		2021		EAPC, 1990–2021
	Number	ASR	Number	ASR	
Global	10,12,673.52 (9,26,459.09–11,16,136.38)	103.78 (94.16–114.22)	15,42,022.95 (1,393,487.43–16,68,706.96)	66.82 (60.12–72.39)	−1.48 (−1.59 to −1.36)
SDI quintiless					
High SDI	4,42,793.06 (4,07,759.68–4,67,473.79)	144.68 (132.90–152.93)	5,53,794.52 (4,90,401.20–5,92,740.81)	91.67 (82.36–97.66)	−1.56 (−1.69 to −1.43)
High-middle SDI	3,22,773.26 (2,98,265.88–3,56,160.50)	127.83 (117.30–140.75)	5,13,894.48 (4,65,327.24–5,52,806.57)	94.85 (85.62–102.12)	−0.95 (−1.09 to −0.8)
Middle SDI	1,16,789.29 (1,05,121.15–1,30,718.95)	48.39 (43.18–54.25)	2,40,332.41 (2,15,603.30–2,66,812.73)	34.36 (30.62–38.27)	−1.2 (−1.29 to −1.11)
Low-middle SDI	95,015.43 (66,719.68–1,38,115.70)	62.66 (43.61–90.99)	1,75,122.43 (1,39,831.87–2,21,776.83)	48.08 (38.20–61.04)	−0.91 (−0.96 to −0.86)
Low SDI	33,493.66 (23,950.47–45,650.06)	58.62 (41.14–80.94)	56,776.58 (43,961.50–72,177.36)	44.59 (34.34–56.88)	−0.99 (−1.07 to −0.9)
SDI regions					
Andean Latin America	4413.81 (3380.22–5535.78)	84.96 (65.08–106.46)	9533.55 (7271.64–12,224.21)	60.80 (46.45–77.86)	−1.09 (−1.23 to −0.95)
Australasia	7739.29 (6775.00–8706.28)	120.63 (105.26–135.82)	10,254.34 (8556.43–11,882.85)	64.59 (54.24–74.75)	−2.04 (−2.19 to −1.88)
Caribbean	7398.84 (6478.50–8460.90)	108.27 (94.69–123.74)	11,607.99 (9675.51–13,812.90)	78.02 (65.06–92.88)	−1.13 (−1.2 to −1.06)
Central Asia	7555.69 (6676.14–8777.84)	60.88 (53.66–71.12)	15,819.76 (13,559.44–18,530.10)	77.06 (66.30–89.55)	0.48 (0.35–0.61)
Central Europe	80,276.38 (75,172.22–85,463.19)	203.02 (189.30–216.43)	92,099.08 (82,561.82–1,01,193.54)	146.08 (130.95–160.63)	−1.17 (−1.37 to −0.96)
Central Latin America	35,338.50 (33,157.44–37,445.14)	170.75 (159.40–181.31)	84,055.25 (72,203.49–95,204.24)	125.87 (108.21–142.44)	−0.92 (−1.03 to −0.81)
Central Sub-Saharan Africa	3216.00 (2104.55–4886.60)	60.03 (39.63–90.14)	7448.68 (4372.13–11,529.73)	56.59 (33.35–87.73)	−0.2 (−0.26 to −0.15)
East Asia	13,164.76 (10,142.18–15,790.61)	6.84 (5.07–8.25)	24,414.49 (20,180.16–28,963.26)	4.44 (3.64–5.27)	−1.72 (−2.33 to −1.12)
Eastern Europe	1,58,890.38 (1,44,142.80–1,85,357.60)	209.62 (189.76–244.17)	3,03,009.98 (2,73,088.77–3,31,107.29)	308.84 (278.18–337.58)	1.3 (1.11–1.48)
Eastern Sub-Saharan Africa	8420.55 (5609.49–11,343.51)	45.36 (29.68–62.27)	17,028.05 (9966.96–25,047.55)	40.84 (23.74–60.78)	−0.46 (−0.54 to −0.37)
High-income Asia Pacific	26,923.00 (24,461.08–29,169.03)	50.23 (45.33–54.48)	72,627.63 (59,062.84–81,882.21)	48.26 (40.55–54.05)	0.13 (−0.09 to 0.36)
High-income North America	1,61,353.73 (1,47,711.96–1,70,929.93)	165.06 (151.40–174.79)	2,02,172.38 (1,81,363.46–2,15,061.12)	111.02 (100.27–117.86)	−1.56 (−1.75 to −1.37)
North Africa and Middle East	23,725.22 (16,760.26–31,171.91)	56.82 (39.70–75.25)	39,406.93 (31,894.98–49,019.40)	35.20 (28.37–43.83)	−1.7 (−1.81 to −1.59)
Oceania	57.23 (38.97–81.09)	7.01 (4.77–9.95)	140.54 (99.31–188.68)	6.56 (4.62–8.76)	0.04 (−0.11 to 0.18)
South Asia	93,454.34 (56,752.41–1,48,330.89)	65.91 (39.20–105.12)	1,67,505.93 (1,22,120.23–2,26,378.78)	44.99 (32.70–60.81)	−1.36 (−1.44 to −1.27)
Southeast Asia	11,658.13 (8158.35–16,637.48)	20.55 (14.08–29.83)	26,549.04 (20,525.19–36,675.64)	17.84 (13.63–24.96)	−0.57 (−0.66 to −0.49)
Southern Latin America	25,787.00 (22,864.65–28,807.10)	209.85 (185.58–234.56)	30,793.61 (26,932.41–34,540.42)	126.65 (110.91–142.04)	−1.5 (−1.61 to −1.39)
Southern Sub-Saharan Africa	2818.80 (1910.60–3753.81)	40.54 (26.84–54.23)	7780.05 (5896.55–9261.65)	51.73 (38.55–61.87)	0.96 (0.67–1.26)
Tropical Latin America	48,570.12 (44,909.42–51,976.67)	207.78 (190.56–222.82)	86,986.95 (78,274.28–94,696.87)	124.13 (111.44–135.21)	−1.84 (−1.91 to −1.76)
Western Europe	2,79,452.59 (2,54,111.42–3,00,065.35)	169.85 (154.22–182.46)	3,05,586.11 (2,63,851.84–3,32,981.63)	109.12 (95.97–118.30)	−1.42 (−1.55 to −1.29)
Western Sub-Saharan Africa	12,459.16 (9153.54–16,884.80)	52.89 (38.75–71.21)	27,202.61 (20,051.87–34,614.13)	51.95 (38.39–65.42)	−0.08 (−0.19 to 0.03)

ASR = age-standardized rate (per 1,00,000 population), CI = confidence interval, DALY = disability-adjusted life year, EAPC = estimated annual percentage change, SDI = sociodemographic index, VID = vascular intestinal diseases.

### 3.2. Sociodemographic disparities in the burden of vascular intestinal diseases among middle-aged and elderly: a global perspective

VID among middle-aged and elderly populations exhibits marked variations across different SDI regions. While high-SDI regions reported the highest absolute number of VID cases, the ASR demonstrated a declining trend. In 2021, the incidence of VID in high-SDI regions reached 6,00,002.79 cases (95% UI: 4,53,314.44–7,65,794.92), with an ASR of 104.48 (95% UI: 77.76–135.36), representing a decline from 111.68 (95% UI: 78.76–150.19) in 1990, corresponding to an EAPC of –0.15% (95% CI: –0.25% to –0.05%; Table 1). Conversely, ASRs increased in low-SDI and low-middle-SDI regions, with EAPCs of 0.36% (95% CI: 0.34–0.38%) and 0.66% (95% CI: 0.61–0.70%), respectively.

A similar pattern was observed in prevalence rates. The ASR of VID prevalence in high-SDI regions declined from 13.60/1,00,000 population (95% UI: 10.77–16.99) in 1990 to 12.89 (95% UI: 10.47–15.69) in 2021. In contrast, low-SDI regions experienced an increase in prevalence ASR from 1.30 (95% UI: 0.91–1.79) to 1.61 (95% UI: 1.22–2.09) over the same period (Table 2). Similarly, trends in DALYs reflected region-specific differences. All SDI regions experienced a decline in DALY ASRs, though the magnitude of reduction varied. The most pronounced decrease occurred in high-SDI regions, with an EAPC of –1.56% (95% CI: –1.69% to –1.43%), while the smallest decline was observed in low-middle-SDI regions, with an EAPC of –0.91% (95% CI: –0.96% to –0.86%; Table 3).

### 3.3. Regional disparities in the burden of vascular intestinal diseases among middle-aged and elderly: a global burden of disease study

The global burden of VID among middle-aged and elderly populations varies significantly across geographic regions, reflecting complex epidemiological patterns and healthcare system challenges. In 2021, the highest incidence of VID was reported in high-income North America, with 2,85,549.95 cases (95% UI: 2,20,724.96–3,53,204.92) and an ASR of 156.87/1,00,000 population (95% UI: 120.30–195.92). Despite this, the region experienced a declining trend, with an EAPC of –0.54% (95% CI: –0.68% to –0.41%; Table 1). In contrast, the North Africa and Middle East region exhibited the fastest-growing ASR, with an EAPC of 1.28% (95% CI: 1.20–1.36%). Prevalence trends revealed similarly diverse patterns. The Andean Latin America region experienced the most notable increase, with an EAPC of 2.17% (95% CI: 2.11–2.23%), while Tropical Latin America showed a declining trend, with an EAPC of –0.73% (95% CI: –0.87% to –0.58%; Table 2). Regarding DALYs, Eastern Europe reported the highest ASR in 2021 at 308.84/1,00,000 population (95% UI: 278.18–337.58), accompanied by an increasing trend (EAPC = 1.30%, 95% CI: 1.11–1.48%). In contrast, most other regions experienced declining DALY trends, with Tropical Latin America showing the most pronounced reduction (EAPC = –1.84%, 95% CI: –1.91% to –1.76%; Table 3).

### 3.4. Global heterogeneity in the burden of vascular intestinal diseases among middle-aged and elderly: a 204-country analysis

The prevalence of VID among middle-aged and elderly populations across 204 countries and regions exhibits notable geographic disparities and temporal trends. In terms of incidence, Equatorial Guinea demonstrated the fastest growth, with an ASR EAPC of 2.93% (95% CI: 2.70–3.16%), rising from 15.88 (95% UI: 10.59–22.52) in 1990 to 34.35 (95% UI: 22.65–48.82) in 2021 (Table S1, Supplemental Digital Content, https://links.lww.com/MD/Q994). In contrast, Sweden experienced the most significant decline, with an EAPC of –1.74% (95% CI: –2.08% to –1.40%). A similar trend was observed for prevalence. Equatorial Guinea and Taiwan, China, recorded the highest increases, both with an EAPC of 4.03%, while Sweden saw the largest reduction, with an EAPC of –2.37% (95% CI: –2.49% to –2.24%; Table S2, Supplemental Digital Content, https://links.lww.com/MD/Q994). Regarding DALYs, Georgia experienced a dramatic increase, with an EAPC of 17.63% (95% CI: 15.55–19.74%), as its ASR surged from 2.49 (95% UI: 1.99–3.13) in 1990 to 144.48 (95% UI: 108.43–187.03) in 2021. Conversely, Bahrain showed the most significant decline, with an EAPC of –4.11% (95% CI: –4.43% to –3.80%; Table S3, Supplemental Digital Content, https://links.lww.com/MD/Q994). These findings reveal the complex dynamics of the global VID burden, reflecting disparities in population demographics, healthcare systems, and socioeconomic development. Notably, some high-income countries such as the United States, Norway, and Italy experienced declining VID burdens, whereas several low- and middle-income countries (LMICs), including Kyrgyzstan and the Republic of Moldova, showed rising trends, underscoring the persistent challenges of global health inequities. To further characterize how VID incidence and prevalence evolve across age, period, and birth cohort, age-, period-, and cohort-specific curves are provided in Figures S1 and S2, Supplemental Digital Content, https://links.lww.com/MD/Q994.

### 3.5. Temporal trends in vascular intestinal diseases: a joinpoint regression analysis from 1990 to 2021

**Figure 2.**
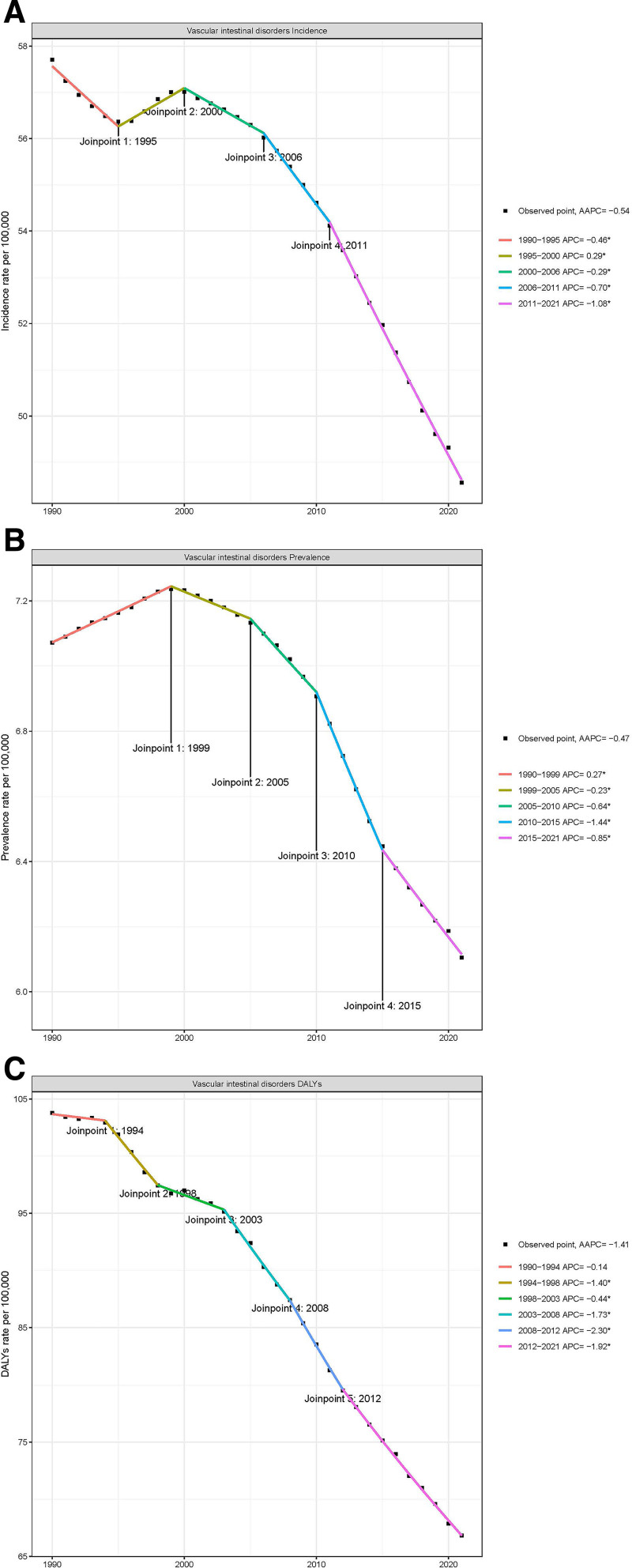
Joinpoint regression analysis of age-standardized rates for vascular intestinal diseases in middle-aged and elderly population from 1990 to 2021. (A) Joinpoint regression analysis of age-standardized incidence rate of VID; (B) joinpoint regression analysis of age-standardized prevalence rate of VID; and (C) joinpoint regression analysis of age-standardized DALY rate of VID. DALY = disability-adjusted life year, VID = vascular intestinal diseases.

The epidemiological characteristics of VID exhibited complex changing patterns from 1990 to 2021. Joinpoint regression analysis revealed distinct trends in incidence, prevalence, and disease burden (Fig. 2). The incidence of VID experienced several turning points, showing a declining trend from 1990 to 1995 (APC = –0.46), followed by a brief increase from 1995 to 2000 (APC = 0.29). Afterward, the incidence consistently declined, with a more pronounced decrease after 2011 (APC = –1.08). Overall, the AAPC for incidence from 1990 to 2021 was –0.54 (Fig. 2A).

The trend in VID prevalence showed a slightly different pattern. It increased between 1990 and 1999 (APC = 0.27), then began to decline, with the most significant reduction occurring from 2010 to 2015 (APC = –1.44). The AAPC for prevalence from 1990 to 2021 was –0.47 (Fig. 2B).

The change in disease burden, measured by DALYs, was the most striking. Despite a brief increase from 1994 to 1998 (APC = 1.40), the overall trend was downward, with an accelerated decline after 2008 (APC = –2.30). The AAPC for DALYs from 1990 to 2021 reached –1.41, indicating a significant reduction in the overall VID burden (Fig. 2C). The long-term trajectories of the absolute number of VID cases, crude rates, and ASR for incidence, prevalence, and DALYs from 1990 to 2021 are depicted in Figure S3, Supplemental Digital Content, https://links.lww.com/MD/Q994.

### 3.6. Age-period-cohort analysis of middle-aged and elderly vascular intestinal diseases: incidence and prevalence patterns

**Figure 3.**
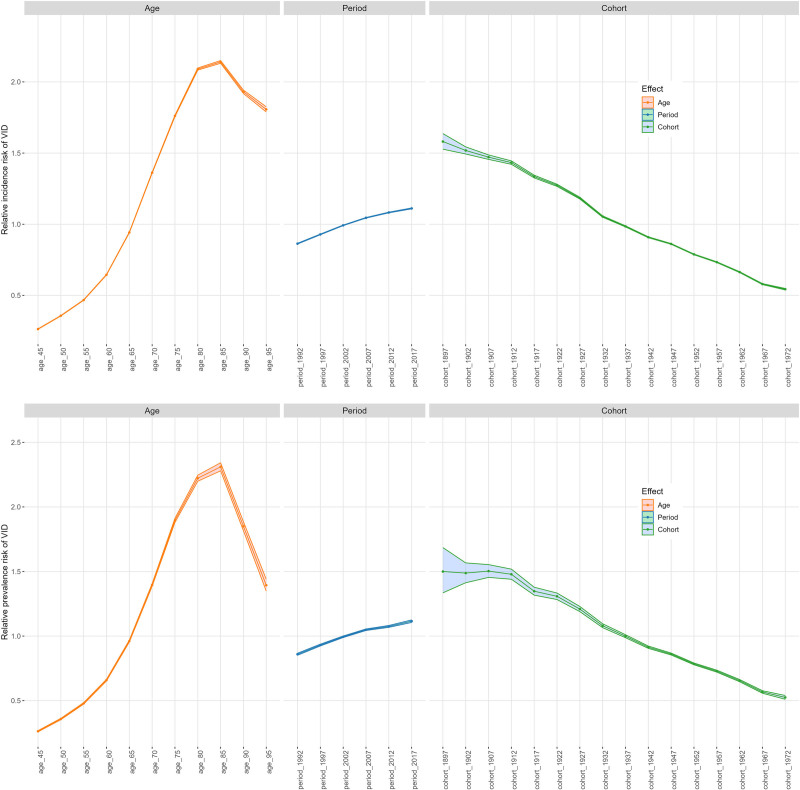
Age-period-cohort analysis of vascular intestinal diseases in middle-aged and elderly population.

The APC analysis revealed complex patterns in the incidence and prevalence of VID among middle-aged and elderly populations (Table 4). The age effect showed a significant increase in VID risk with advancing age, peaking in the 85 to 89 age group (incidence RR = 2.140, 95% CI: 2.131–2.150; prevalence RR = 2.311, 95% CI: 2.279–2.342), followed by a slight decline. The period effect demonstrated an upward trend, reflecting improvements in diagnostic techniques and increased disease awareness. From 1992 (incidence RR = 0.864, 95% CI: 0.861–0.866; prevalence RR = 0.859, 95% CI: 0.851–0.867) to 2017 (incidence RR = 1.111, 95% CI: 1.108–1.114; prevalence RR = 1.115, 95% CI: 1.105–1.125), both incidence and prevalence steadily increased. The cohort effect showed a notable decline, with a significant reduction from the 1897 birth cohort (incidence RR = 1.581, 95% CI: 1.527–1.637; prevalence RR = 1.500, 95% CI: 1.335–1.685) to the 1972 birth cohort (incidence RR = 0.543, 95% CI: 0.538–0.549; prevalence RR = 0.525, 95% CI: 0.510–0.540). This trend likely reflects improvements in healthcare services, better living conditions, and healthier lifestyle practices among more recent cohorts. Notably, the consistency between incidence and prevalence APC patterns suggests that these factors similarly influence both disease onset and persistence. These results are illustrated in Figure 3, which presents the age, period, and cohort effects on the global burden of VID (Fig. 3). More detailed age-, period-, and cohort-specific curves for VID incidence and prevalence, stratified by age group and calendar year, are presented in Figures S1 and S2, Supplemental Digital Content, https://links.lww.com/MD/Q994.

**Table 4:** Relative risks (RRs) of middle-aged and elderly VID incidence and prevalence for sexes due to age, period, and birth cohort effects.

Factor	Incidence		Prevalence	
	RR (95% CI)	*P*	RR (95% CI)	*P*
Age (yr)				
45–49	0.263 (0.262–0.265)	<.001	0.263 (0.259–0.268)	<.001
50–54	0.357 (0.355–0.359)	<.001	0.358 (0.353–0.363)	<.001
55–59	0.467 (0.466–0.469)	<.001	0.479 (0.473–0.484)	<.001
60–64	0.645 (0.643–0.647)	<.001	0.659 (0.653–0.665)	<.001
65–69	0.942 (0.939–0.945)	<.001	0.961 (0.954–0.969)	<.001
70–74	1.362 (1.359–1.366)	<.001	1.396 (1.385–1.406)	<.001
75–79	1.761 (1.756–1.766)	<.001	1.896 (1.880–1.912)	<.001
80–84	2.090 (2.083–2.098)	<.001	2.222 (2.199–2.246)	<.001
85–89	2.140 (2.131–2.150)	<.001	2.311 (2.279–2.342)	<.001
90–94	1.930 (1.918–1.942)	<.001	1.850 (1.815–1.886)	<.001
95–99	1.807 (1.789–1.825)	<.001	1.393 (1.348–1.440)	<.001
Period				
1992	0.864 (0.861–0.866)	<.001	0.859 (0.851–0.867)	0.001
1997	0.928 (0.926–0.931)	<.001	0.930 (0.924–0.937)	<.001
2002	0.992 (0.990–0.994)	<.001	0.995 (0.989–1.000)	.068
2007	1.045 (1.043–1.047)	<.001	1.049 (1.043–1.055)	<.001
2012	1.082 (1.080–1.085)	<.001	1.075 (1.068–1.082)	<.001
2017	1.111 (1.108–1.114)	<.001	1.115 (1.105–1.125)	<.001
Birth cohort				
1897	1.581 (1.527–1.637)	<.001	1.500 (1.335–1.685)	<.001
1902	1.518 (1.493–1.544)	<.001	1.488 (1.413–1.567)	<.001
1907	1.471 (1.456–1.487)	<.001	1.503 (1.454–1.553)	<.001
1912	1.433 (1.421–1.445)	<.001	1.479 (1.440–1.518)	<.001
1917	1.335 (1.326–1.345)	<.001	1.347 (1.317–1.378)	<.001
1922	1.274 (1.266–1.281)	<.001	1.308 (1.283–1.333)	<.001
1927	1.183 (1.177–1.190)	<.001	1.209 (1.189–1.229)	<.001
1932	1.054 (1.049–1.059)	<.001	1.080 (1.065–1.095)	<.001
1937	0.985 (0.981–0.989)	<.001	0.999 (0.988–1.011)	.864
1942	0.909 (0.906–0.912)	<.001	0.914 (0.905–0.923)	<.001
1947	0.862 (0.859–0.864)	<.001	0.861 (0.854–0.868)	<.001
1952	0.788 (0.786–0.791)	<.001	0.785 (0.778–0.791)	<.001
1957	0.734 (0.731–0.736)	<.001	0.728 (0.721–0.736)	<.001
1962	0.664 (0.661–0.667)	<.001	0.655 (0.647–0.664)	<.001
1967	0.579 (0.576–0.583)	<.001	0.567 (0.558–0.577)	<.001
1972	0.543 (0.538–0.549)	<.001	0.525 (0.510–0.540)	<.001

CI = confidence interval, RR = relative risk, VID = vascular intestinal diseases.

The APC analysis also revealed significant sex-specific differences in VID risk patterns (Table S4, Supplemental Digital Content, https://links.lww.com/MD/Q994). Both sexes experienced increasing VID risk with age, although peak ages and relative risks differed. Women reached peak incidence and prevalence in the 85 to 89 age group (incidence RR = 2.206, 95% CI: 2.194–2.217; prevalence RR = 2.446, 95% CI: 2.407–2.485), while men peaked slightly earlier and at lower relative risks (incidence RR = 2.026, 95% CI: 2.009–2.044; prevalence RR = 2.137, 95% CI: 2.081–2.195). The period effect showed an upward trend in both sexes, but men experienced a more pronounced increase from 1992 (incidence RR = 0.868, 95% CI: 0.863–0.873) to 2017 (incidence RR = 1.126, 95% CI: 1.120–1.132). Similarly, the cohort effect revealed a declining trend, though women from earlier birth cohorts (e.g., 1897) faced relatively higher risks (incidence RR = 1.600, 95% CI: 1.538–1.664; prevalence RR = 1.547, 95% CI: 1.356–1.765). By the 1972 birth cohort, the sex gap narrowed significantly (men: incidence RR = 0.551, 95% CI: 0.543–0.559; women: RR = 0.535, 95% CI: 0.529–0.543), suggesting reduced gender disparities in VID burden over time. Sex-specific age, period, and cohort curves for VID incidence and prevalence in males and females are presented in Figures S4 and S5, Supplemental Digital Content, https://links.lww.com/MD/Q994, respectively.

### 3.7. Sociodemographic index and vascular intestinal diseases: global trends and regional disparities

**Figure 4.**
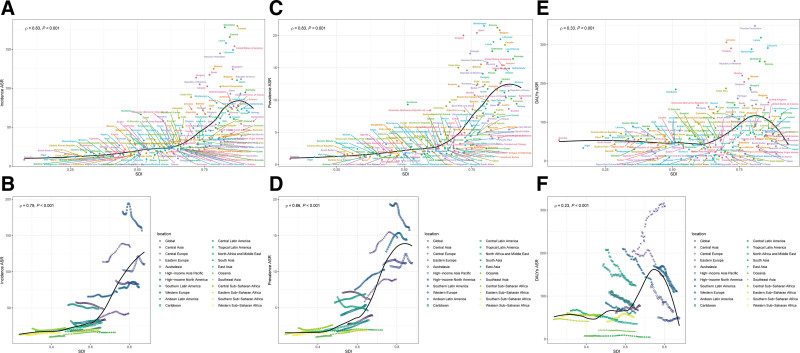
Correlation between sociodemographic index (SDI) and age-standardized rates of vascular intestinal diseases in middle-aged and elderly population in 2021. (A) Correlation between SDI and age-standardized incidence rate across 204 countries and territories; (B) correlation between SDI and age-standardized incidence rate across 21 GBD regions; (C) correlation between SDI and age-standardized prevalence rate across 204 countries and territories; (D) correlation between SDI and age-standardized prevalence rate across 21 GBD regions; (E) correlation between SDI and age-standardized DALY rate across 204 countries and territories; and (F) correlation between SDI and age-standardized DALY rate across 21 GBD regions. DALY = disability-adjusted life year, GBD = global burden of disease, SDI = sociodemographic index.

The burden of VID exhibits a complex and diverse association with the SDI, reflecting significant geographic disparities and the influence of varying levels of development worldwide. Both the age-standardized incidence rate and ASPR showed a strong positive correlation with SDI (*R* = 0.79 and *R* = 0.86, respectively; *P* < .001), suggesting that increased socioeconomic development may enhance VID diagnosis and reporting rates (Fig. 4A–D; country-level: 4A, 4C; region-level: 4B, 4D). Corresponding patterns for the DALY rate are shown in Figure 4E, F (country-level: 4E; region-level: 4F).

### 3.8. Decomposition analysis of changes in DALYs for middle-aged and elderly VID: global, SDI, and gender perspectives

**Figure 5.**
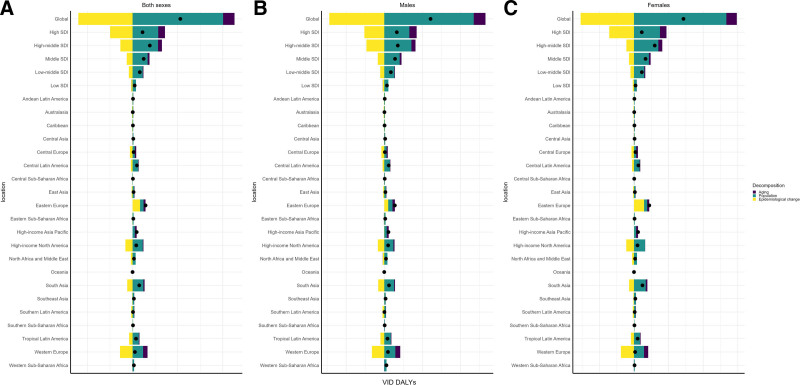
Decomposition analysis of changes in DALYs for vascular intestinal diseases in middle-aged and elderly population from 1990 to 2021. (A) Decomposition analysis results for both sexes combined; (B) decomposition analysis results for males; and (C) decomposition analysis results for females. DALY = disability-adjusted life year.

Globally, the burden of VID among middle-aged and elderly populations, measured in DALYs, increased significantly from 1990 to 2021, with a net change of 5,29,349.42 DALYs (Table S5, Supplemental Digital Content, https://links.lww.com/MD/Q994). This increase was primarily driven by population growth, contributing 10,03,828.12 DALYs (189.63%). However, epidemiological improvements offset part of this growth, reducing the burden by 6,00,466.14 DALYs (–113.43%). Population aging also played a role, adding 1,25,987.45 DALYs (23.8%). These findings suggest that while epidemiological advances have had a positive impact, demographic factors remain the primary drivers of the increasing VID burden in older populations. These decomposition results are presented in Figure 5A, which summarizes the relative contributions of demographic and epidemiological factors to the global increase in DALYs (Fig. 5A).

Significant regional differences in VID burden were observed across SDI regions (Table S5, Supplemental Digital Content, https://links.lww.com/MD/Q994). In high-SDI regions, the overall increase in DALYs was 1,11,001.46, driven mainly by population growth (2,84,241.22 DALYs, 256.07%) and aging (74,705.11 DALYs, 67.3%). However, this was substantially offset by reductions due to epidemiological changes (–2,47,944.87 DALYs, –223.37%). In contrast, low-SDI regions experienced a much smaller net increase of 23,282.92 DALYs, driven primarily by population growth (37,692.55 DALYs, 161.89%), with a minimal contribution from aging (–226.63 DALYs, –0.97%). Middle-SDI regions exhibited a pattern between these 2 extremes, highlighting the influence of socioeconomic development on VID burden trends.

Sex-specific analysis revealed that the VID burden increase was slightly higher among women (2,85,977.66 DALYs) compared to men (2,43,371.76 DALYs; Table S5, Supplemental Digital Content, https://links.lww.com/MD/Q994). Globally, the contribution of population growth was higher in women (5,33,675.89 DALYs, 186.61%) than in men (4,69,875.69 DALYs, 193.07%), while the positive impact of epidemiological improvements was also greater for women (–3,08,274.65 DALYs, –107.8%) compared to men (–2,88,476.78 DALYs, –118.53%). Figure 5B, C presents the sex-specific decomposition of DALY changes for males and females, respectively. Notably, in high-SDI regions, the contribution of aging to the VID burden was significantly higher in women (37,672.49 DALYs, 83.81%) than in men (38,300.14 DALYs, 57.99%), reflecting the potential impact of increased female life expectancy on VID burden in more developed areas. Taken together, these visualizations in Figure 5 clearly illustrate the differing roles of demographic and epidemiological transitions in shaping sex-specific VID burdens over time.

### 3.9. Evolving health inequalities in vascular intestinal diseases: a global perspective from 1990 to 2021

**Figure 6.**
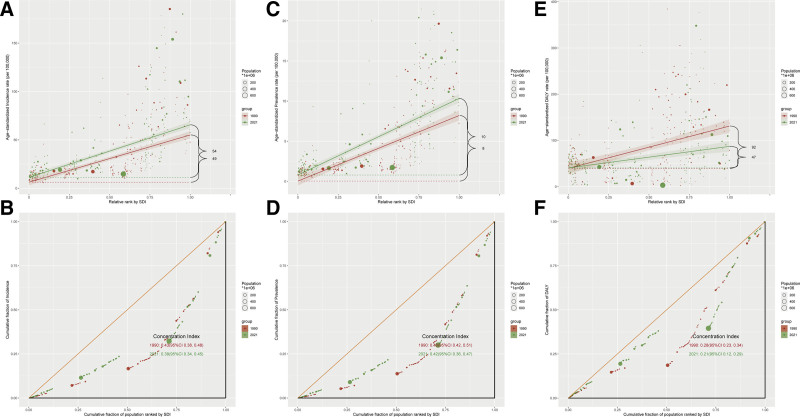
Health inequality analysis of vascular intestinal diseases in middle-aged and elderly population from 1990 to 2021. (A) Slope index of inequality for age-standardized incidence rate; (B) concentration index for age-standardized incidence rate; (C) slope index of inequality for age-standardized prevalence rate; (D) concentration index for age-standardized prevalence rate; (E) slope index of inequality for age-standardized DALY rate; and (F) concentration index for age-standardized DALY rate. DALY = disability-adjusted life year.

Global health inequalities related to VID have shown gradual improvement over time, though significant disparities persist. SII analysis indicated that the incidence rate inequality decreased from 54 in 1990 to 49 in 2021 (Fig. 6A), while prevalence rate inequality declined from 10 to 8 over the same period (Fig. 6C), reflecting a slight narrowing of the gap between high-SDI and low-SDI regions. Notably, the SII for the DALYs rate underwent a substantial shift, dropping from 92 in 1990 to –47 in 2021 (Fig. 6E), suggesting a significant redistribution of the VID burden.

Concentration index analysis further confirmed this trend. The CI for incidence decreased from 0.43 (95% CI: 0.38–0.48) in 1990 to 0.39 (95% CI: 0.34–0.45) in 2021 (Fig. 6B), while the CI for prevalence declined from 0.46 (95% CI: 0.42–0.51) to 0.42 (95% CI: 0.36–0.47; Fig. 6D). Similarly, the CI for DALYs dropped from 0.28 (95% CI: 0.23–0.34) to 0.21 (95% CI: 0.12–0.29; Fig. 6F).

These indicators collectively suggest that although the VID burden remains concentrated in high-SDI regions, the degree of inequality has diminished over time. The most pronounced improvement was observed in the DALY rate, indicating that advances in disease management and prevention strategies have likely yielded greater benefits in regions with varying levels of socioeconomic development.

### 3.10. Projected trends in the global burden of vascular intestinal diseases among middle-aged and elderly: 2022 to 2040

**Figure 7.**
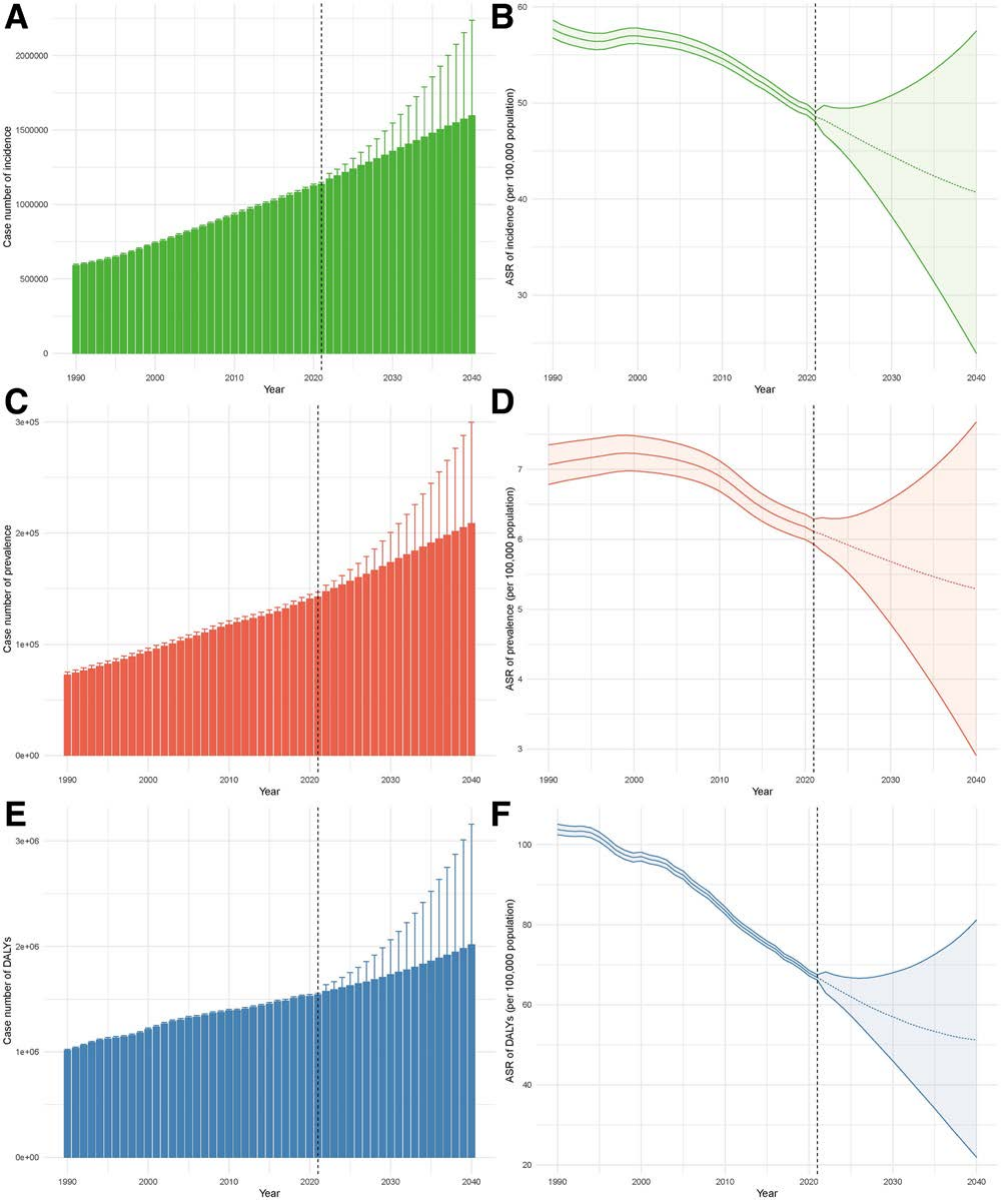
Projected trends in the global burden of vascular intestinal diseases in middle-aged and elderly population from 1990 to 2040. (A) Projected number of incident cases of VID from 1990 to 2040; (B) projected age-standardized incidence rate of VID from 1990 to 2040; (C) projected number of prevalent cases of VID from 1990 to 2040; (D) projected age-standardized prevalence rate of VID from 1990 to 2040; (E) projected number of DALYs due to VID from 1990 to 2040; and (F) projected age-standardized DALY rate of VID from 1990 to 2040. DALY = disability-adjusted life year, VID = vascular intestinal diseases.

The global burden of VID among middle-aged and elderly populations is projected to undergo significant changes between 2022 and 2040, with distinct trends across various epidemiological indicators. Model validation through posterior predictive checks demonstrated satisfactory predictive accuracy, with mean absolute percentage error values below 10% for both incidence and prevalence projections, and approximately 95% of observed values falling within the predicted 95% credible intervals, indicating robust model calibration and reliability for future burden estimation. According to the BAPC disease burden projection model, the absolute number of VID cases is expected to increase steadily, while all ASRs are projected to decline (Table S6, Supplemental Digital Content, https://links.lww.com/MD/Q994). Specifically, the number of VID incident cases is projected to rise from 11,71,709 in 2022 to 15,96,062 in 2040, reflecting a 36.2% increase. However, the corresponding ASR is expected to decrease from 48.24/1,00,000 in 2022 to 40.71/1,00,000 in 2040, representing a 15.6% decline. Figure 7A, B illustrates the projected number of incident cases and the age-standardized incidence rate of VID from 1990 to 2040. Similarly, VID prevalence is projected to increase from 1,47,273 cases in 2022 to 2,08,473 cases in 2040, a 41.6% rise, while the prevalence ASR is expected to decline from 6.07/1,00,000 to 5.29/1,00,000, a reduction of 12.8%. Figure 7C, D presents the predicted trends in the number of prevalent cases and the ASPR over the same period. The most pronounced changes are projected in the burden measured by DALYs. The number of DALYs attributable to VID is expected to increase from 15,72,880 in 2022 to 20,16,029 in 2040, a 28.2% rise. However, the corresponding DALY ASR is projected to decline significantly from 65.54/1,00,000 to 51.18/1,00,000, a reduction of 21.9%. Figure 7E, F demonstrates the future burden of VID in terms of total DALYs and the corresponding age-standardized DALY rate.

These projections suggest that while the absolute VID burden is likely to increase due to population aging and growth, the individual risk may decrease over time, potentially reflecting advances in prevention, diagnosis, and treatment strategies. Nonetheless, the continued rise in case numbers underscores the need for global healthcare systems to prioritize VID management and allocate sufficient resources for its prevention and treatment.

## 4. Discussion

This study provides a comprehensive analysis of the global trends in the incidence, prevalence, and disease burden of VID among middle-aged and elderly populations from 1990 to 2021, with projections extending to 2040. The findings reveal that while the ASRs of VID have declined, indicating a reduction in individual risk, the continued rise in absolute case numbers remains a critical public health challenge worldwide. As a relatively rare condition situated at the intersection of vascular and gastrointestinal surgery, VID’s evolving burden reflects the complexities of the global health landscape. In recent years, advancements in medical technology, the widespread adoption of early diagnostic methods, and improvements in health-related behaviors have played a crucial role in reducing ASRs. However, factors such as population aging, shifts in the global disease spectrum, and unequal distribution of healthcare resources have driven a steady increase in the absolute number of VID cases. This “dual burden” underscores the need for public health policies that extend beyond primary prevention to include healthcare system strengthening. Addressing the growing demand for healthcare services due to demographic shifts requires a more integrated approach, focusing not only on reducing VID incidence but also on enhancing healthcare infrastructure and ensuring equitable resource allocation.

The analysis demonstrated a consistent decline in the ASR of VID since 1990, particularly in high-income countries and regions with high-SDI scores, aligning with previous research findings.^[4]^ This trend reflects the rapid advancement of global medical technologies and the success of public health interventions. The decline in VID ASR can be attributed mainly to significant improvements in disease prevention and management strategies. For instance, large-scale early screening programs and minimally invasive vascular interventional procedures widely implemented in high-income countries have played a critical role in reducing both incidence and mortality rates.^[11]^ Additionally, the effective management of cardiovascular risk factors such as hypertension and diabetes has indirectly lowered VID risk.^[12]^ Despite declining ASRs, the absolute number of VID cases has increased significantly over the past 32 years, driven primarily by population growth and aging. Our findings indicate that population aging alone contributed approximately 23.8% to the increase in VID burden, while population growth accounted for an even larger share. This trend underscores the need for global health policies to focus more on managing aging-related diseases while enhancing health education and early intervention programs for older populations. Although high-SDI regions have reported the largest absolute number of VID cases, they also exhibited the most pronounced declines in ASR. This outcome is closely linked to the advantages enjoyed by developed countries in terms of medical technology, health education, and policy implementation. However, these regions still face the challenge of an increasing total number of cases due to the continuous growth of their elderly populations. As a result, even as individual risks decline, the overall demand for healthcare resources continues to rise, potentially exacerbating the financial burden on healthcare systems in high-income countries. In contrast, low-SDI regions displayed different trends, with limited ASR reductions and even upward trends in some areas. Several contributing factors may explain this phenomenon: inadequate healthcare resources and infrastructure that limit access to diagnosis and treatment; insufficient management of chronic conditions such as hypertension and diabetes, which are prevalent but often poorly controlled in these regions^[13,14]^ and slow improvements in living conditions, making it difficult to adopt healthier dietary and lifestyle habits associated with reduced VID risk. These findings highlight the pressing need to address global health inequities. Strengthening disease screening and diagnostic capacities in low-SDI regions through international aid and policy support is essential for improving local healthcare systems. Focused investments in healthcare infrastructure and public health education could help mitigate the growing VID burden and promote health equity worldwide.

Our findings indicate that the global burden of VID has increased slightly more among women than men over the past 3 decades. Although this pattern is partially explained by demographic factors – particularly the greater longevity of women – it also suggests more intricate interactions involving biological, behavioral, and structural determinants. For example, estrogen-mediated vascular protection in premenopausal women may delay the onset of VID; however, the risk rises significantly post-menopause due to declining hormone levels and an increased prevalence of associated comorbidities, such as atherosclerosis and atrial fibrillation.^[15,16]^

Furthermore, gender disparities in healthcare-seeking behaviors and access to medical services may exacerbate these differences. In certain LMICs, sociocultural norms and structural barriers frequently restrict women’s timely access to healthcare, resulting in delayed diagnoses and more advanced disease at presentation.^[17,18]^ Conversely, men are more commonly exposed to modifiable risk factors such as tobacco use, uncontrolled hypertension, and occupational stress, potentially contributing to an earlier onset of VID.

These findings underscore the necessity for sex-specific preventive strategies. In high-SDI settings, targeted screening programs and effective management of cardiovascular risk factors among elderly women could mitigate complications associated with advanced VID. In LMICs, policies should prioritize interventions designed to enhance gender-equitable access to diagnostic and emergency care for acute abdominal conditions. Future studies are warranted to disaggregate VID subtypes by sex and age, elucidating both the pathophysiological mechanisms and healthcare-related factors underlying observed gender disparities.

This study highlights significant geographic disparities in the global burden of VID. For example, high-income North America reported the highest VID burden globally in 2021, coupled with a marked decline in ASR. This pattern likely reflects the application of advanced medical technologies and effective disease management strategies. However, the absolute increase in disease burden also underscores the region’s continued dependence on healthcare resources. Treating VID often requires costly interventional procedures and extended hospital care,^[19,20]^ which may place substantial pressure on healthcare resource allocation. In contrast, the North Africa and Middle East region exhibited the highest ASR growth rate for VID globally. This trend may be attributed to several factors, including lifestyle changes driven by rapid urbanization, such as the adoption of Western diets and reduced physical activity. Additionally, the pace of healthcare infrastructure development has lagged behind population growth, while chronic disease management and early screening programs remain underdeveloped. These findings emphasize the need for health interventions tailored to the cultural and healthcare system contexts of these regions to ensure effective disease management. Similarly, Eastern Europe reported a significantly higher VID ASR than the global average, with the highest DALY ASR recorded in 2021. This concerning trend could be linked to unhealthy dietary habits characterized by high salt and fat intake, along with elevated smoking and alcohol consumption rates.^[21,22]^ Moreover, healthcare systems in this region may have struggled to adapt to evolving disease patterns during economic transitions, further exacerbating the VID burden. Targeted policy reforms focusing on health promotion, risk factor management, and healthcare system strengthening are essential to mitigating the growing VID burden in these high-risk regions.

Our findings extend and complement previous VID burden analyses, particularly the work by Jiang et al, which examined global VID burden through 2019 using GBD 2019 data across all age groups.^[4]^ While their study reported declining ASR alongside increasing absolute case numbers – a pattern consistent with our observations – our analysis provides several distinctive contributions. First, by specifically focusing on middle-aged and elderly populations (≥45 years), we captured the demographic segment bearing the greatest VID burden, with mortality rates escalating from 1% to 3% in younger adults to nearly 50% after age 95.^[1]^ Second, utilizing the GBD 2021 dataset allowed us to incorporate pandemic-era data and extend the analysis through 2021, revealing continued burden evolution in the post-2019 period. Third, our implementation of BAPC modeling to project trends through 2040 addresses a critical gap in long-term VID burden forecasting, providing essential evidence for healthcare system planning in an aging global population. These methodological advances and extended temporal scope enable more targeted policy recommendations for the populations and timeframes most relevant to upcoming healthcare challenges.

Although this study indicates some improvements in global VID burden inequality, significant disparities persist between high-SDI and low-SDI regions. It is important to acknowledge that the UIs reported throughout our analysis reflect inherent variations in data quality and estimation methods across different regions. The wider UIs observed in low-SDI regions (e.g., incidence in low-SDI regions: 95% UI ranging from 1.22 to 2.09/1,00,000) compared to high-SDI regions suggest greater data variability and potentially less robust surveillance systems in resource-limited settings. These uncertainty ranges do not diminish the validity of observed trends but rather underscore the need for strengthened disease surveillance infrastructure in regions with wider confidence bounds. Notably, despite these uncertainties, the consistent directionality of trends across multiple metrics (incidence, prevalence, and DALYs) and the narrow CIs for EAPC estimates provide robust evidence for the regional disparities and temporal patterns identified in this study. Future research should focus on the following key areas:

Socioeconomic development and disease burden dynamics: Further exploration is needed to understand the dynamic relationship between socioeconomic development and disease burden. Investigating how socioeconomic factors influence long-term disease trends and assessing the direct impact of economic growth on healthcare investment could provide valuable insights for policy development.

Precision medicine and personalized interventions: Leveraging advances in genomics and big data analytics could help identify population-specific susceptibility to VID and inform tailored intervention strategies. Early screening and treatment protocols could be designed based on patients genetic backgrounds and lifestyle factors, enabling more effective and individualized disease management.

Environmental factors and disease risk: The high incidence of VID is closely linked to environmental factors such as pollution, dietary habits, and climate change.^[23,24]^ Future research should quantify the specific contributions of these environmental determinants to VID burden and inform targeted policy interventions aimed at mitigating their impact. By advancing research through interdisciplinary collaboration and comprehensive data analysis, we can develop more effective strategies to address the global burden of VID while reducing health disparities across regions and between genders. This approach would support the development of more equitable, evidence-based global health policies and resource allocation frameworks. To translate these epidemiological insights into tangible health improvements, targeted interventions must address both the documented disparities and the projected burden increases.

This study projects that although the ASR of VID is expected to decline further by 2040, the absolute number of cases will continue to rise, necessitating immediate, evidence-based policy actions across multiple healthcare domains. Based on our findings, we propose the following actionable strategies: first, healthcare systems must implement age-stratified screening protocols targeting high-risk populations aged ≥65 years, particularly in high-SDI regions where absolute case burdens are highest. Specific measures should include: integration of VID risk assessment into routine geriatric health examinations; development of clinical decision support tools incorporating age-specific risk calculators; and establishment of rapid-access diagnostic pathways for elderly patients presenting with abdominal symptoms.^[3]^ Second, to address the rising burden in low-SDI regions (EAPC 0.36–0.66%), international health organizations should prioritize capacity-building initiatives, including: training programs for emergency physicians in early VID recognition; telemedicine networks connecting rural facilities to vascular surgery expertise; and subsidized access to essential diagnostic imaging technologies such as CT angiography.^[3]^ Third, given the observed birth cohort effect showing lower risk in younger generations, primary prevention campaigns should focus on modifiable vascular risk factors. Evidence-based interventions include: national programs promoting Mediterranean-style dietary patterns to reduce atherosclerotic burden; smoking cessation initiatives targeting middle-aged adults (45–64 years) before peak VID risk period; and hypertension and diabetes control programs integrated into community health centers.^[5]^ Fourth, to optimize resource allocation in the context of growing case numbers, healthcare planners should: expand endovascular intervention capacity, which has demonstrated favorable outcomes compared to open surgery in high-risk elderly patients^[3]^; develop regional VID treatment networks with hub-and-spoke models ensuring 24/7 access to emergency revascularization; and implement value-based payment models incentivizing early diagnosis and minimally invasive approaches. Finally, addressing gender disparities requires targeted approaches, including heightened clinical suspicion for VID in elderly women, who demonstrate higher mortality rates,^[1]^ and development of sex-specific diagnostic algorithms accounting for atypical presentations more common in female patients.

## 5. Limitation

This study leverages the comprehensive GBD 2021 database spanning 204 countries and integrates multiple analytical approaches – including Joinpoint regression, APC analysis, and Bayesian projection modeling – to provide the first age-focused assessment of VID burden dynamics through 2040. For the first time in the field of VID research, multiple analytical methods, including Joinpoint regression, APC analysis, and the BAPC prediction model, were integrated to reveal the epidemiological characteristics and dynamic trends of VID. Additionally, the study provides detailed stratified analyses based on the SDI, geographic regions, and gender-specific differences, offering practical insights for policy development. This study has several limitations. First, data quality disparities exist across regions, with potential underreporting in low-income settings affecting burden estimates. The wider UIs in these regions reflect this limitation. Future research should focus on improving data collection and validation in these regions. Second, while this study primarily investigates macro-level trends, it does not examine the specific impact of individual risk factors such as dietary patterns, smoking, and body mass index on VID burden. Lastly, although the BAPC model provides reliable projections, its results remain subject to uncertainties related to future advances in medical technology and policy changes.

## 6. Conclusions

This study comprehensively analyzed global trends in the burden of VID among middle-aged and elderly populations from 1990 to 2021 and projected their future trajectory from 2022 to 2040. The findings reveal the complex dynamics of VID burden, reflecting both progress and ongoing challenges in global public health.

First, although the absolute number of VID cases has increased, the widespread decline in ASRs suggests a reduction in individual disease risk. This trend can likely be attributed to advances in medical technology, improved health awareness, and the effective implementation of preventive strategies worldwide. However, substantial disparities persist across SDI regions and countries. While high-SDI regions reported the highest absolute number of VID cases, they also experienced the most significant decline in ASR. In contrast, low-SDI regions showed slower improvements or even rising trends, underscoring the persistent issue of global health inequity.

Second, APC analysis revealed a consistent increase in VID risk with advancing age, accompanied by a clear birth cohort effect, where younger cohorts exhibited significantly lower disease risks. This finding highlights the potential value of early intervention and health education in reducing future VID burdens. Additionally, gender-specific analysis indicated that while the disease burden increased slightly more in women than in men, this trend was primarily driven by demographic dynamics rather than inherent differences in disease risk.

Third, decomposition analysis showed that population growth and aging were the primary drivers of the increasing VID burden, while epidemiological improvements partially offset this growth. This finding underscores the critical role of demographic changes in shaping disease burden trends while reflecting the positive impact of medical advancements in mitigating VID development.

Finally, health inequality analysis and future projections suggest that although global VID burden inequality has lessened to some extent, substantial disparities remain. By 2040, the absolute number of VID cases is expected to continue rising, while ASRs are projected to decline further, indicating continued improvements in individual risk. However, healthcare systems must be prepared to manage the growing number of cases through enhanced resource allocation and more robust healthcare infrastructure.

## Acknowledgments

The authors gratefully acknowledge all participants of the GBD 2021 for their contribution.

## Author contributions

**Conceptualization:** Zengkai Zhou.

**Data curation:** Zengkai Zhou.

**Formal analysis:** Zengkai Zhou.

**Methodology:** Zengkai Zhou.

**Project administration:** Zengkai Zhou.

**Resources:** Zengkai Zhou.

**Software:** Zengkai Zhou.

**Supervision:** Huayou Luo.

**Validation:** Zengkai Zhou.

**Visualization:** Zengkai Zhou.

**Writing – original draft:** Zengkai Zhou, Ruo Shu.

**Writing – review & editing:** Zengkai Zhou, Tong Zhang.

## Supplementary Material


