# Supplementary material for: Evolution of the global burden of vascular intestinal diseases in middle-aged and elderly population: Trend analysis from 1990 to 2021 and projections to 2040
Source: Medicine (Baltimore). 2025 Dec 26;104(52):e46774. doi: 10.1097/MD.0000000000046774 (PMC12746939; doi:10.1097/MD.0000000000046774)
Supplement: Supplementary file 1 [file medi-104-e46774-s001.pdf]

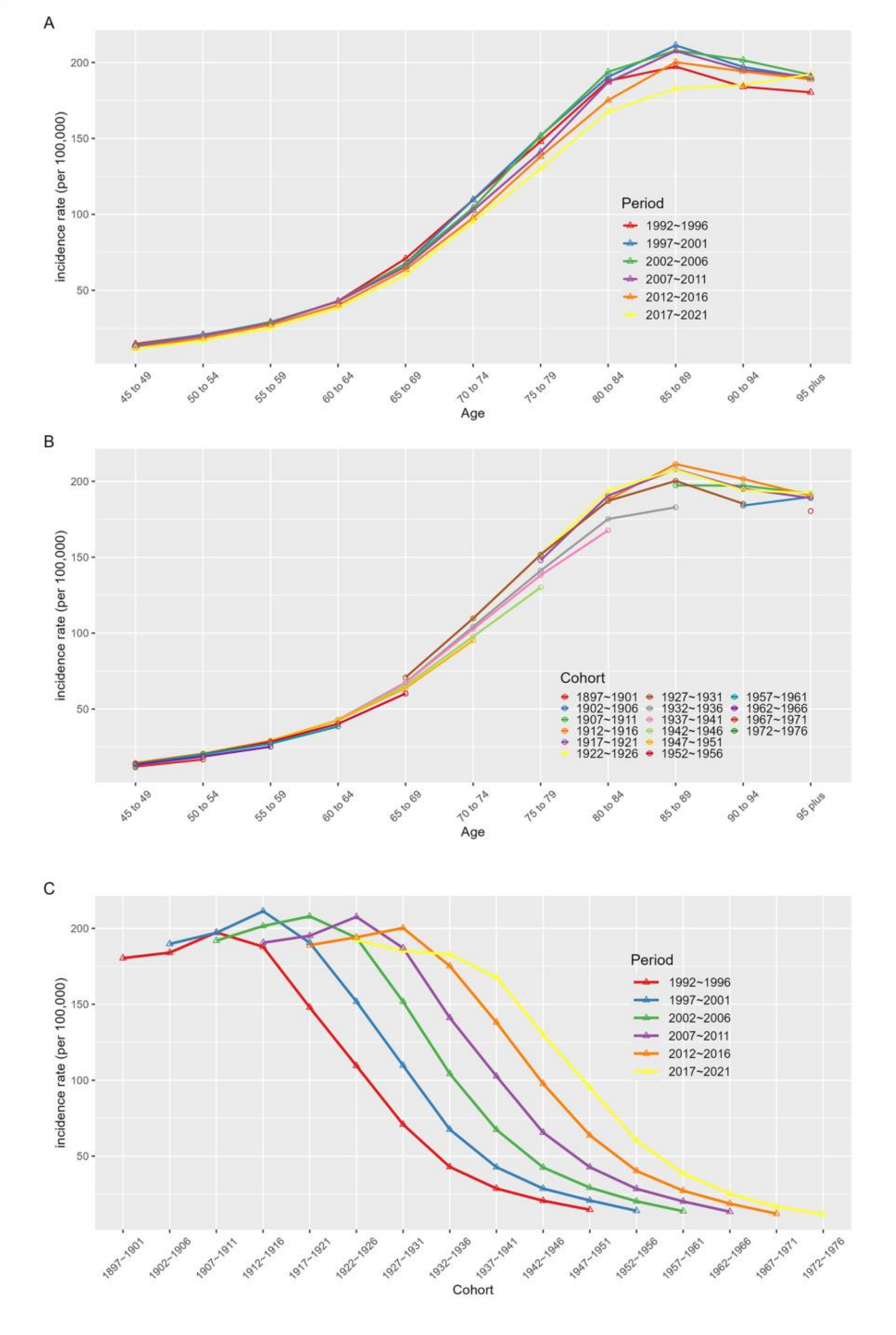

Supplementary Figure 1. Period and cohort effects on incidence of Vascular Intestinal Disorders in middle-aged and elderly population

A: Period effects on incidence rate by age group

B: Cohort effects on incidence rate by age group

C: Period effects on incidence rate by calendar year

Abbreviations: VID, vascular intestinal diseases; ASR, age-standardized rate; DALY, disability-adjusted life year; EAPC, estimated annual percentage change; UI, uncertainty interval; SDI, Socio-demographic Index.

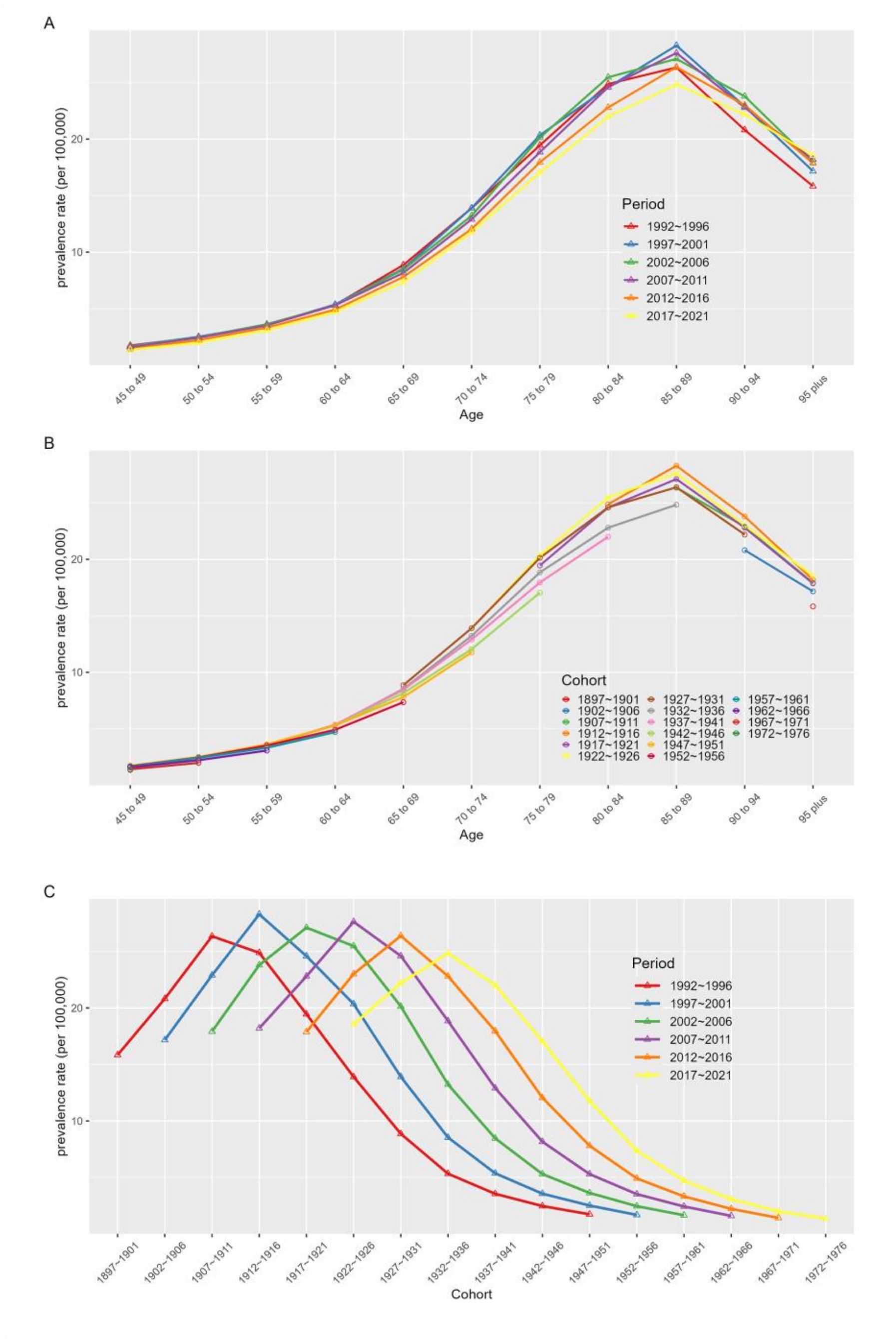

Supplementary Figure 2. Period and cohort effects on prevalence of Vascular Intestinal Disorders in middle-aged and elderly population

A: Period effects on prevalence rate by age group

B: Cohort effects on prevalence rate by age group

C: Period effects on prevalence rate by calendar year

Abbreviations: VID, vascular intestinal diseases; ASR, age-standardized rate; DALY, disability-adjusted life year; EAPC, estimated annual percentage change; UI, uncertainty interval; SDI, Socio-demographic Index.

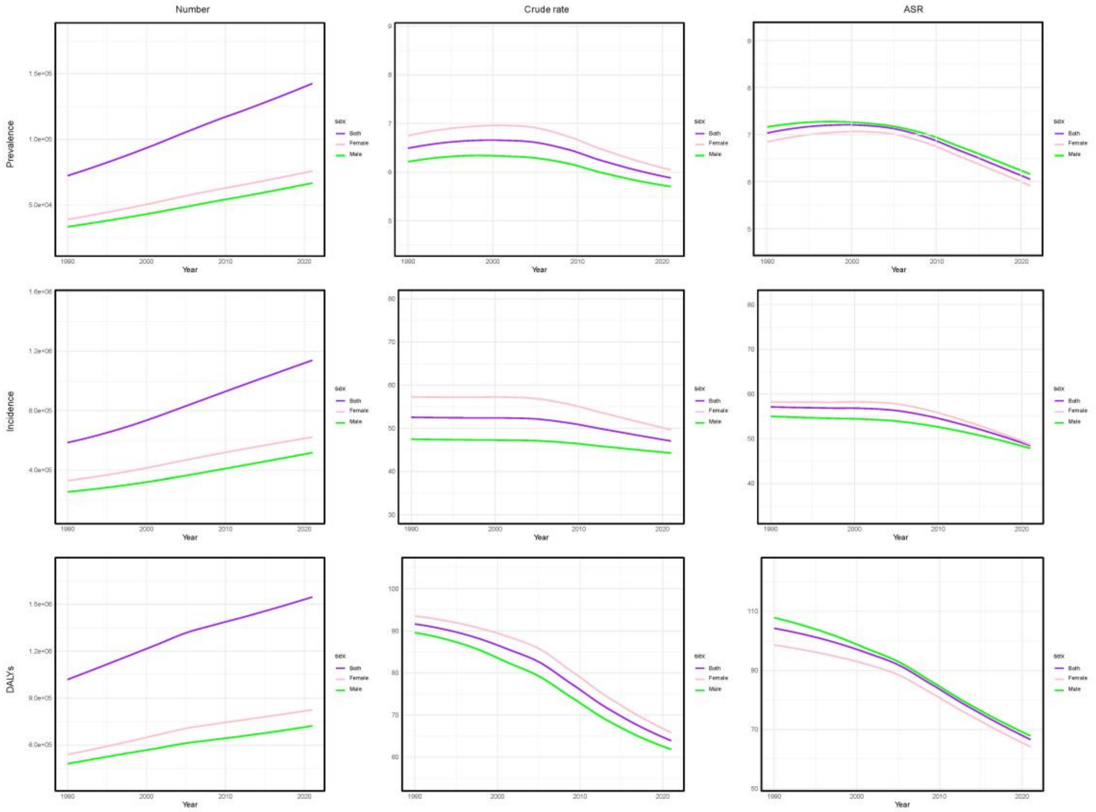

Supplementary Figure 3. Trends in the number of cases, crude rates, and age-standardized rates of incidence, prevalence, and DALYs for Vascular Intestinal Disorders in middle-aged and elderly population from 1990 to 2021

Abbreviations: VID, vascular intestinal diseases; ASR, age-standardized rate; DALY, disability-adjusted life year; UI, uncertainty interval.

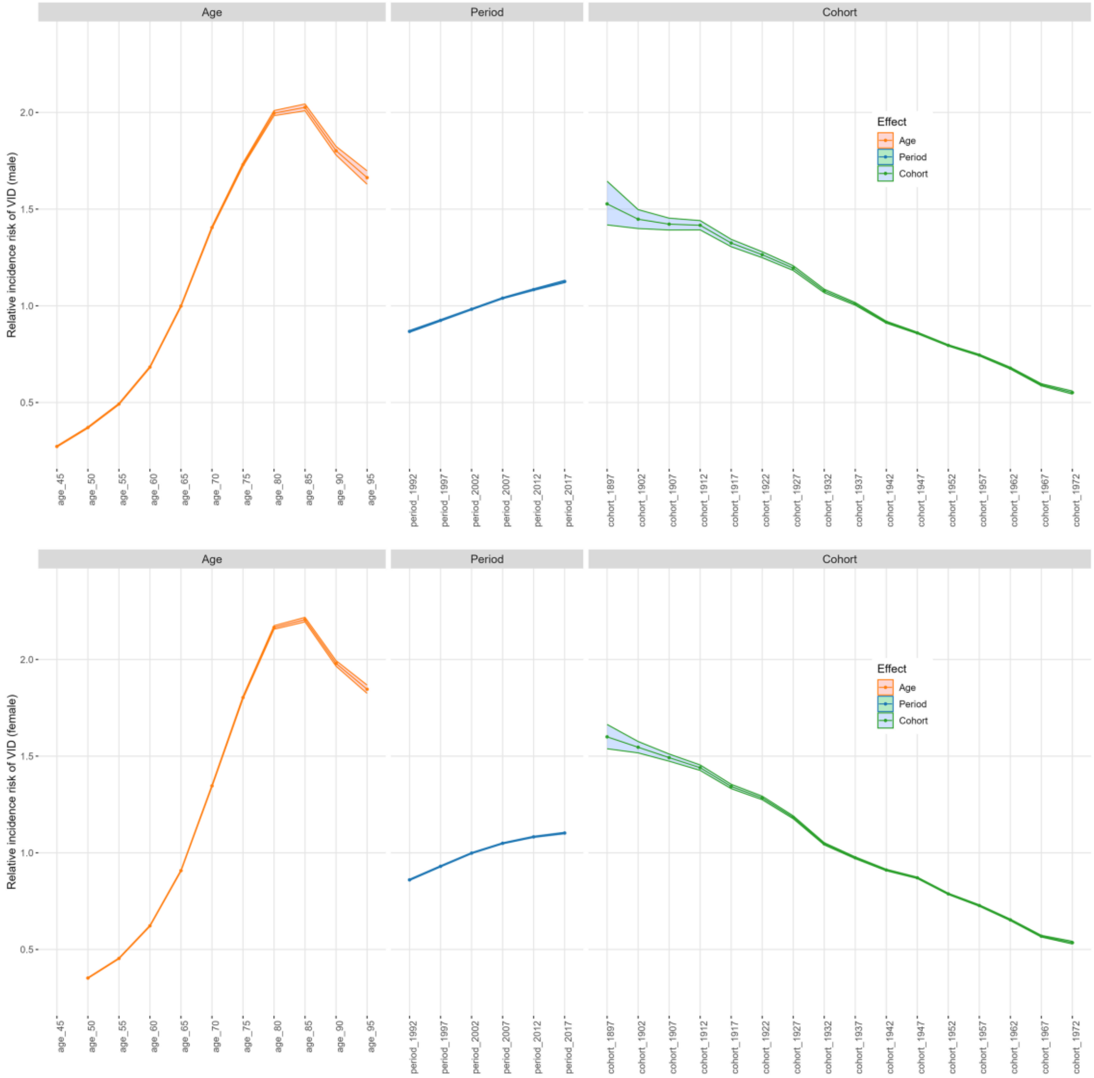

Supplementary Figure 4. Age-Period-Cohort analysis of incidence of Vascular Intestinal Disorders in middle-aged and elderly population by sex

(Upper panel: Males; Lower panel: Females)

Abbreviations: VID, vascular intestinal diseases; RR, relative risk; UI, uncertainty interval.

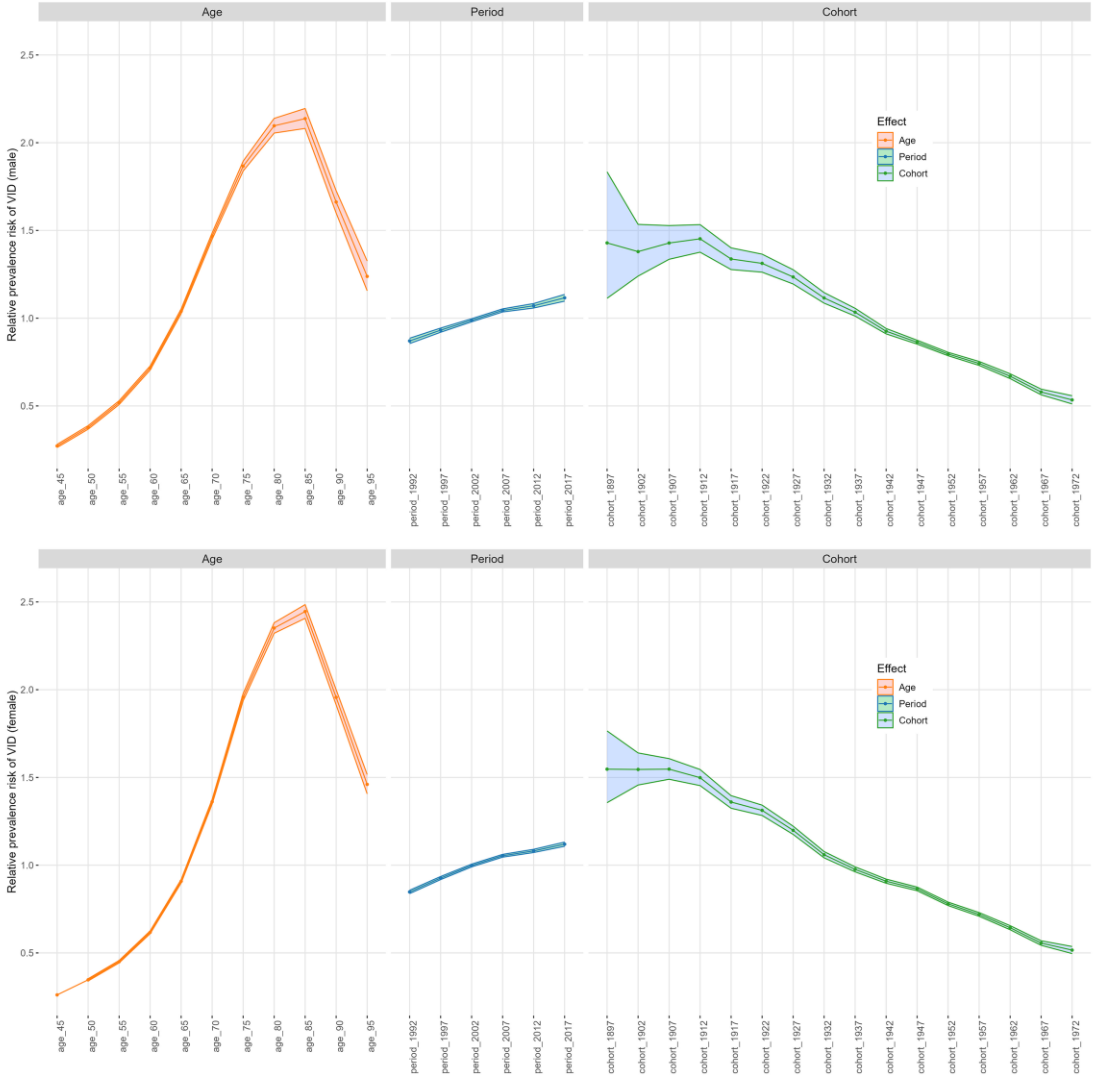

Supplementary Figure 5. Age-Period-Cohort analysis of prevalence of Vascular Intestinal Disorders in middle-aged and elderly population by sex

(Upper panel: Males; Lower panel: Females)

Abbreviations: VID, vascular intestinal diseases; RR, relative risk; UI, uncertainty interval.

Table S1. Global and regional incidence of middle-aged and elderly VID in 1990 and 2021, and EAPC of ASR from 1990 to 2021.

Table S1. Global and regional incidence of middle-aged and elderly VID in 1990 and 2021, and EAPC of ASR from 1990 to 2021.					
**Location**	**1990**		**2021**		**EAPC, 1990-2021**
	**Number**	**ASR**	**Number**	**ASR**	
Afghanistan	246.21 (159.69–356.60)	13.53 (8.98–19.27)	424.85 (273.42–618.85)	16.19 (11.09–22.37)	0.71 (0.58 to 0.84)
Albania	84.87 (50.03–132.34)	15.24 (9.20–23.34)	249.33 (155.38–375.87)	20.93 (12.95–31.80)	1.28 (1.11 to 1.45)
Algeria	668.81 (421.05–977.36)	21.18 (13.60–30.53)	3018.68 (1948.39–4356.28)	31.99 (21.03–45.40)	1.38 (1.34 to 1.41)
American Samoa	0.98 (0.60–1.47)	16.42 (10.42–24.07)	2.46 (1.57–3.57)	18.50 (12.02–26.36)	0.33 (0.26 to 0.4)
Andorra	9.14 (5.71–13.61)	58.69 (36.92–87.31)	32.09 (20.93–47.85)	74.94 (48.68–112.04)	0.71 (0.64 to 0.79)
Angola	136.06 (87.42–197.45)	14.81 (9.90–20.87)	615.41 (408.78–891.16)	21.72 (14.91–30.53)	1.36 (1.22 to 1.5)
Antigua and Barbuda	2.60 (1.65–3.82)	18.45 (11.44–27.51)	6.38 (3.90–9.58)	21.54 (13.37–31.94)	0.43 (0.33 to 0.53)
Argentina	5469.67 (3698.26–7558.79)	62.15 (42.08–85.88)	10295.32 (6923.21–14515.61)	66.52 (44.49–94.21)	0.2 (0.12 to 0.27)
Armenia	370.60 (243.31–530.82)	52.06 (34.65–73.93)	868.93 (608.90–1222.66)	73.20 (51.37–103.12)	1.29 (1.22 to 1.35)
Australia	4401.26 (2972.42–6232.91)	81.04 (54.54–115.08)	10789.37 (7058.53–15781.07)	83.91 (54.31–123.71)	0.06 (-0.02 to 0.15)
Austria	1851.12 (1493.87–2281.78)	54.15 (43.55–67.02)	3747.62 (2691.85–4894.08)	73.40 (52.29–96.66)	1.36 (0.81 to 1.9)
Azerbaijan	237.02 (149.13–352.04)	17.13 (10.98–25.05)	698.83 (454.37–1005.54)	25.04 (16.64–35.34)	1.52 (1.33 to 1.72)
Bahamas	14.60 (9.44–21.17)	35.33 (23.05–50.73)	41.09 (26.20–59.67)	36.43 (23.50–52.41)	0.23 (0.19 to 0.26)
Bahrain	22.34 (14.67–31.85)	65.64 (45.28–90.44)	133.94 (83.86–199.17)	66.88 (45.00–94.89)	-0.44 (-0.66 to -0.22)
Bangladesh	2197.30 (1445.55–3140.34)	18.06 (12.08–25.48)	10251.34 (6737.32–14525.98)	27.50 (18.23–38.70)	1.49 (1.44 to 1.54)
Barbados	16.15 (10.36–23.30)	21.05 (13.25–30.91)	33.95 (21.53–49.97)	23.99 (15.13–35.56)	0.33 (0.25 to 0.4)
Belarus	2380.24 (1632.83–3239.75)	67.31 (46.39–91.50)	5619.06 (3839.16–7731.50)	124.96 (85.36–172.18)	2.4 (2.26 to 2.54)
Belgium	3815.76 (2605.88–5351.93)	88.98 (60.23–126.32)	7244.05 (4925.11–10566.35)	111.63 (74.34–164.32)	0.61 (0.4 to 0.82)
Belize	3.44 (2.11–5.22)	13.77 (8.47–20.84)	14.03 (8.66–21.18)	17.02 (10.76–25.10)	0.65 (0.5 to 0.81)
Benin	43.75 (27.40–65.34)	8.48 (5.42–12.51)	161.52 (103.24–235.54)	12.24 (8.08–17.36)	1.16 (1.11 to 1.21)
Bermuda	5.80 (3.76–8.39)	34.79 (22.73–50.05)	14.01 (9.01–20.86)	37.39 (23.72–56.26)	0.13 (0.05 to 0.2)

Bhutan	9.90 (6.28–14.64)	16.23 (10.68–23.34)	52.29 (34.38–74.83)	32.79 (21.73–46.67)	2.43 (2.35 to 2.51)
Bolivia (Plurinational State of)	148.47 (97.52–211.93)	18.66 (12.54–26.21)	571.68 (372.65–823.97)	24.56 (16.32–34.96)	0.97 (0.93 to 1.02)
Bosnia and Herzegovina	199.29 (127.92–300.52)	18.89 (12.44–27.92)	481.48 (329.36–691.77)	27.98 (18.93–40.56)	1.68 (1.42 to 1.95)
Botswana	29.34 (18.72–42.85)	19.96 (13.09–28.58)	114.97 (77.13–160.75)	29.13 (19.99–39.91)	1.11 (0.94 to 1.28)
Brazil	7442.79 (5590.82–9877.01)	32.33 (24.64–42.43)	19855.07 (14389.03–26957.45)	28.90 (21.05–39.12)	0.37 (0.01 to 0.73)
Brunei Darussalam	22.89 (14.89–33.43)	80.75 (54.31–114.32)	96.44 (65.39–133.69)	102.21 (71.37–137.24)	0.71 (0.55 to 0.88)
Bulgaria	843.47 (545.39–1241.30)	25.06 (16.28–36.87)	1457.58 (1040.78–2051.49)	36.40 (25.52–51.86)	1.62 (1.44 to 1.8)
Burkina Faso	94.00 (59.03–140.37)	8.82 (5.76–12.84)	302.62 (194.98–438.32)	13.00 (8.61–18.48)	1.46 (1.4 to 1.52)
Burundi	58.79 (38.98–84.64)	10.72 (7.26–15.24)	143.06 (98.37–205.00)	12.75 (9.06–17.85)	0.56 (0.49 to 0.64)
Cabo Verde	5.86 (3.71–8.63)	9.37 (5.84–13.98)	19.37 (12.39–28.43)	16.60 (10.81–24.03)	1.88 (1.79 to 1.98)
Cambodia	93.15 (57.20–140.35)	8.51 (5.44–12.47)	399.20 (252.83–583.26)	12.98 (8.50–18.54)	1.54 (1.48 to 1.59)
Cameroon	108.52 (67.34–163.08)	9.68 (6.24–14.14)	432.90 (273.44–635.84)	13.77 (8.99–19.76)	1.11 (1.07 to 1.15)
Canada	14387.72 (10051.48–18910.25)	161.37 (112.03–213.50)	36293.14 (25232.16–47536.68)	180.05 (124.14–238.55)	0.42 (0.32 to 0.51)
Central African Republic	46.38 (30.32–66.62)	16.99 (11.53–23.82)	93.72 (63.66–133.42)	17.70 (12.52–24.42)	0.06 (0 to 0.12)
Chad	68.49 (44.12–99.03)	9.37 (6.12–13.44)	182.70 (119.19–264.51)	12.52 (8.44–17.67)	0.99 (0.95 to 1.03)
Chile	1694.04 (1125.53–2380.54)	64.03 (42.85–89.44)	5207.13 (3985.33–6668.75)	73.51 (56.20–94.25)	0.4 (-0.03 to 0.83)
China	37119.12 (21818.68–56992.32)	17.23 (10.46–25.88)	87988.23 (56705.27–128195.83)	14.78 (9.61–21.42)	-0.69 (-0.83 to -0.54)
Colombia	2068.97 (1431.49–2827.03)	45.73 (32.14–61.50)	9363.92 (6261.03–13354.84)	62.08 (41.53–88.55)	1.04 (0.94 to 1.14)
Comoros	6.46 (4.29–9.33)	14.06 (9.62–19.89)	19.17 (13.14–27.39)	15.85 (11.11–22.26)	0.32 (0.27 to 0.37)
Congo	51.44 (34.17–74.47)	20.30 (13.85–28.82)	163.07 (104.29–238.36)	24.99 (16.67–35.52)	0.69 (0.67 to 0.71)
Cook Islands	0.82 (0.52–1.20)	24.28 (15.64–35.07)	2.64 (1.71–3.81)	36.08 (23.38–52.32)	1.11 (1.06 to 1.16)
Costa Rica	340.53 (229.93–479.77)	75.30 (51.08–105.64)	1326.98 (901.55–1883.29)	88.58 (60.26–125.81)	0.57 (0.53 to 0.61)
Coted'Ivoire	97.12 (59.51–147.00)	9.79 (6.34–14.21)	367.61 (231.24–544.36)	13.10 (8.56–18.77)	0.77 (0.69 to 0.84)
Croatia	810.60 (591.80–1127.34)	50.49 (37.20–69.75)	1248.07 (959.64–1650.63)	47.82 (36.22–63.91)	-0.1 (-0.23 to 0.03)

Cuba	1120.25 (737.05–1645.41)	40.29 (26.54–59.10)	2731.58 (1886.96–3917.55)	49.90 (34.33–71.85)	0.8 (0.74 to 0.87)
Cyprus	52.27 (34.77–76.12)	25.67 (17.29–37.05)	180.28 (127.62–253.60)	31.63 (22.26–44.70)	0.58 (0.49 to 0.66)
Czechia	1277.98 (873.21–1859.21)	33.80 (22.88–49.49)	2247.34 (1534.50–3298.40)	37.46 (25.12–55.57)	-0.14 (-0.33 to 0.05)
Democratic People's Republic of Korea	675.59 (406.18–1035.39)	15.31 (9.57–22.74)	1777.51 (1112.38–2611.39)	19.44 (12.29–28.23)	0.88 (0.78 to 0.97)
Democratic Republic of the Congo	839.27 (543.34–1218.27)	23.60 (15.82–33.59)	1957.49 (1289.72–2838.46)	21.92 (14.89–31.07)	-0.29 (-0.53 to -0.05)
Denmark	1128.67 (778.21–1616.60)	50.49 (34.34–72.90)	1549.62 (1067.83–2241.75)	46.83 (31.45–68.57)	-0.48 (-0.59 to -0.38)
Djibouti	3.79 (2.40–5.65)	12.66 (8.46–18.09)	22.01 (14.43–32.21)	15.05 (10.32–21.29)	0.55 (0.48 to 0.62)
Dominica	4.81 (3.17–6.98)	30.10 (19.80–43.94)	7.20 (4.77–10.36)	31.60 (21.10–45.29)	0.19 (0.13 to 0.26)
Dominican Republic	150.93 (93.12–226.50)	15.18 (9.54–22.47)	601.80 (387.76–878.12)	22.09 (14.31–32.06)	1.28 (1.18 to 1.38)
Ecuador	285.68 (198.18–392.52)	21.02 (14.79–28.57)	1108.06 (857.54–1433.29)	25.44 (19.77–32.80)	0.69 (0.49 to 0.89)
Egypt	1109.39 (672.79–1686.17)	15.81 (10.08–23.13)	4594.42 (2934.70–6752.71)	28.02 (18.58–39.94)	1.84 (1.77 to 1.9)
El Salvador	156.71 (104.02–222.55)	19.69 (13.21–27.72)	576.83 (381.38–811.54)	33.78 (22.21–47.75)	1.9 (1.71 to 2.09)
Equatorial Guinea	7.53 (4.88–10.92)	15.88 (10.59–22.52)	41.52 (26.72–60.09)	34.35 (22.65–48.82)	2.93 (2.7 to 3.16)
Eritrea	23.22 (15.00–34.35)	9.83 (6.72–13.95)	85.77 (58.58–123.17)	14.29 (10.22–19.85)	1.08 (0.93 to 1.22)
Estonia	692.99 (474.81–947.23)	121.13 (82.97–165.72)	1362.41 (925.46–1866.75)	165.85 (111.36–230.74)	1.09 (0.9 to 1.28)
Eswatini	10.32 (6.38–15.44)	13.17 (8.48–19.14)	27.89 (19.14–38.92)	19.01 (13.38–25.87)	1.18 (1.15 to 1.21)
Ethiopia	614.23 (410.76–875.90)	13.87 (9.61–19.33)	1661.02 (1143.23–2321.85)	16.18 (11.41–22.22)	0.65 (0.51 to 0.79)
Fiji	12.35 (7.62–18.45)	12.84 (8.24–18.62)	33.73 (22.03–48.38)	15.96 (10.71–22.51)	0.61 (0.57 to 0.65)
Finland	1107.42 (744.86–1607.96)	55.41 (37.00–80.84)	2185.30 (1514.13–3206.38)	60.58 (40.68–90.58)	0.1 (0 to 0.19)
France	14843.01 (10459.54–20417.72)	63.55 (44.07–88.74)	27278.55 (18811.96–38498.27)	70.56 (47.48–101.31)	0.27 (0.2 to 0.33)
Gabon	33.21 (21.92–47.75)	22.83 (15.27–32.55)	79.58 (51.30–114.58)	30.34 (20.03–42.89)	0.84 (0.79 to 0.89)
Gambia	9.64 (6.08–14.16)	11.11 (7.26–15.95)	31.74 (20.60–46.76)	12.81 (8.52–18.48)	0.3 (0.21 to 0.4)
Georgia	347.53 (215.59–522.66)	20.31 (12.71–30.31)	545.75 (398.00–722.17)	33.39 (24.22–44.44)	2.08 (1.79 to 2.36)
Germany	30757.99 (20495.29–44044.74)	86.08 (57.09–123.77)	50275.00 (33509.56–72022.00)	94.72 (61.61–137.98)	0.64 (0.16 to 1.13)

Ghana	167.67 (103.73–251.58)	10.86 (6.97–15.86)	612.67 (388.69–894.99)	14.57 (9.58–20.74)	0.85 (0.74 to 0.96)
Greece	2167.30 (1390.15–3180.98)	51.23 (32.63–75.59)	3242.84 (2221.79–4589.56)	49.26 (32.76–71.08)	0.29 (-0.19 to 0.77)
Greenland	11.39 (7.94–15.30)	135.32 (95.98–178.90)	33.74 (24.45–43.93)	178.20 (130.16–230.85)	0.89 (0.86 to 0.93)
Grenada	2.86 (1.82–4.19)	15.13 (9.40–22.55)	7.04 (4.42–10.43)	22.21 (14.14–32.42)	1.14 (1.03 to 1.25)
Guam	4.01 (2.42–6.06)	20.73 (13.04–30.44)	13.24 (8.55–19.07)	22.62 (14.61–32.61)	0.44 (0.39 to 0.49)
Guatemala	144.15 (91.45–212.21)	16.38 (10.96–23.21)	597.03 (394.87–852.46)	20.54 (13.80–28.93)	0.68 (0.49 to 0.86)
Guinea	71.68 (44.89–106.29)	8.40 (5.37–12.31)	158.05 (102.26–227.57)	10.93 (7.25–15.46)	0.76 (0.68 to 0.85)
Guinea-Bissau	9.81 (6.27–14.41)	10.03 (6.63–14.37)	23.13 (15.12–33.24)	13.23 (9.04–18.40)	0.86 (0.79 to 0.93)
Guyana	18.06 (11.63–26.49)	18.01 (11.82–25.99)	39.07 (25.66–56.58)	22.00 (14.70–31.38)	0.67 (0.6 to 0.74)
Haiti	124.94 (81.66–180.36)	15.52 (10.37–22.03)	267.84 (175.06–389.76)	14.41 (9.71–20.42)	-0.15 (-0.2 to -0.1)
Honduras	230.55 (153.89–322.66)	45.63 (30.89–63.21)	1037.41 (700.19–1480.99)	66.38 (45.57–93.44)	1.29 (1.23 to 1.35)
Hungary	1990.37 (1465.56–2719.74)	49.54 (36.28–68.05)	3219.90 (2361.18–4417.70)	59.34 (42.96–82.22)	0.34 (0.24 to 0.43)
Iceland	61.90 (39.51–89.96)	79.40 (50.38–115.98)	121.62 (78.92–174.58)	76.91 (49.36–111.24)	-0.04 (-0.37 to 0.29)
India	20670.56 (13242.26–30178.13)	17.84 (11.88–25.32)	59367.27 (40975.02–82242.40)	19.10 (13.40–26.09)	0.21 (0.14 to 0.29)
Indonesia	3409.38 (2163.82–4997.42)	14.91 (9.88–21.19)	9963.71 (6713.91–14036.58)	18.43 (12.94–25.07)	0.69 (0.64 to 0.74)
Iran (Islamic Republic of)	1707.76 (1036.75–2582.08)	25.99 (16.38–38.27)	4923.70 (3180.26–7088.34)	23.98 (15.89–33.79)	-0.34 (-0.41 to -0.27)
Iraq	309.00 (188.43–471.36)	14.48 (8.99–21.75)	1487.44 (914.62–2227.16)	22.29 (14.30–32.24)	1.68 (1.57 to 1.78)
Ireland	587.65 (384.81–848.37)	51.49 (33.47–75.01)	1580.60 (1059.60–2341.81)	71.93 (47.76–107.13)	1.36 (1.28 to 1.45)
Israel	686.99 (457.59–992.09)	51.57 (34.21–74.88)	2110.99 (1422.61–3123.29)	61.30 (40.79–91.32)	0.4 (0.3 to 0.49)
Italy	22139.33 (14736.95–31963.27)	88.38 (58.16–128.80)	23585.54 (17686.75–30813.38)	55.60 (41.00–73.69)	-1.72 (-2.1 to -1.34)
Jamaica	92.82 (58.39–136.48)	19.47 (12.13–28.90)	173.52 (109.80–250.95)	20.51 (12.97–29.66)	0.24 (0.19 to 0.29)
Japan	51156.58 (32918.06–74077.77)	108.78 (70.01–157.43)	97523.95 (69802.92–129712.99)	110.31 (74.96–153.61)	0.35 (0.17 to 0.53)
Jordan	83.88 (53.18–123.90)	25.90 (16.97–37.24)	719.59 (466.71–1047.85)	36.85 (24.53–52.50)	1.26 (1.19 to 1.34)
Kazakhstan	1326.58 (868.61–1897.05)	39.81 (26.32–56.46)	2491.63 (1643.57–3523.39)	51.51 (34.42–72.30)	1.05 (0.91 to 1.18)

Kenya	400.92 (253.23–594.56)	20.54 (13.25–30.06)	1289.41 (850.17–1850.66)	24.46 (16.66–34.41)	0.85 (0.73 to 0.97)
Kiribati	2.14 (1.41–3.02)	22.17 (14.89–30.97)	4.80 (3.22–6.72)	24.61 (16.96–33.90)	0.19 (0.13 to 0.25)
Kuwait	61.48 (38.61–91.33)	41.55 (27.40–59.08)	462.32 (289.42–676.20)	62.80 (41.10–87.64)	1.7 (1.6 to 1.8)
Kyrgyzstan	138.61 (89.39–203.14)	17.25 (11.23–25.14)	259.63 (163.76–381.00)	20.02 (13.06–28.63)	0.44 (0.36 to 0.53)
Lao People's Democratic Republic	39.21 (24.01–59.14)	7.60 (4.86–11.19)	135.91 (85.82–199.56)	11.57 (7.58–16.49)	1.47 (1.4 to 1.53)
Latvia	1274.61 (893.41–1712.69)	127.42 (89.26–171.57)	1925.44 (1281.82–2620.50)	158.00 (104.44–217.13)	0.33 (0.06 to 0.59)
Lebanon	126.10 (78.27–187.38)	22.16 (14.11–32.29)	662.88 (429.10–941.16)	40.10 (25.78–57.30)	2.06 (2 to 2.12)
Lesotho	25.53 (16.19–37.72)	11.32 (7.31–16.51)	41.86 (29.17–58.03)	15.02 (10.66–20.49)	0.89 (0.85 to 0.92)
Liberia	24.63 (15.40–36.50)	8.46 (5.40–12.37)	62.40 (39.26–93.41)	11.97 (7.92–17.29)	1.34 (1.28 to 1.4)
Libya	114.17 (71.01–170.13)	23.05 (14.70–33.71)	407.96 (256.77–604.85)	29.47 (19.31–42.26)	0.85 (0.7 to 1.01)
Lithuania	1096.66 (746.42–1491.74)	87.76 (59.65–119.39)	2633.96 (1830.03–3563.32)	149.23 (102.31–203.58)	1.45 (1.26 to 1.63)
Luxembourg	131.02 (85.43–188.36)	86.33 (56.09–124.61)	273.00 (181.74–396.15)	93.15 (61.41–136.03)	-0.41 (-0.64 to -0.18)
Madagascar	111.79 (71.42–166.35)	9.50 (6.25–13.83)	303.68 (203.91–445.20)	12.04 (8.50–17.02)	0.78 (0.75 to 0.81)
Malawi	93.04 (61.35–136.20)	10.90 (7.44–15.56)	241.98 (165.38–346.11)	14.11 (9.96–19.74)	0.88 (0.84 to 0.92)
Malaysia	406.67 (260.10–597.96)	17.48 (11.43–25.23)	2519.95 (1659.23–3631.01)	34.56 (23.13–49.27)	2.35 (2.18 to 2.52)
Maldives	2.66 (1.60–4.04)	11.79 (7.47–17.23)	16.44 (10.15–24.72)	19.69 (12.61–28.70)	1.62 (1.47 to 1.77)
Mali	77.76 (47.32–118.58)	7.84 (4.99–11.63)	271.15 (171.67–399.26)	12.09 (7.91–17.40)	1.52 (1.44 to 1.59)
Malta	35.17 (24.64–50.11)	30.22 (21.21–43.01)	97.20 (70.20–134.63)	34.99 (24.58–49.50)	0.07 (-0.08 to 0.23)
Marshall Islands	0.57 (0.36–0.83)	13.86 (9.00–19.94)	1.56 (0.99–2.27)	16.69 (11.01–23.67)	0.49 (0.46 to 0.53)
Mauritania	26.89 (17.27–39.21)	10.51 (6.88–15.15)	78.80 (49.16–115.40)	14.11 (9.01–20.35)	0.74 (0.65 to 0.84)
Mauritius	23.24 (14.09–35.37)	12.38 (7.73–18.49)	84.71 (53.51–124.41)	16.83 (10.74–24.57)	0.79 (0.67 to 0.92)
Mexico	7111.88 (4874.85–9793.07)	65.32 (45.51–88.56)	13996.67 (9905.38–19154.19)	41.72 (29.82–56.67)	-1.69 (-1.81 to -1.58)
Micronesia (Federated States of)	1.67 (1.08–2.41)	13.38 (8.82–19.15)	3.78 (2.42–5.46)	18.87 (12.48–26.74)	1.17 (1.15 to 1.2)
Monaco	11.70 (7.51–17.09)	60.46 (38.11–89.75)	19.19 (12.68–27.81)	71.19 (45.95–104.67)	0.47 (0.39 to 0.55)

Mongolia	43.21 (27.72–62.52)	15.30 (10.01–21.82)	138.85 (88.48–202.03)	21.85 (14.61–30.70)	1.33 (1.21 to 1.44)
Montenegro	111.35 (80.17–154.81)	66.03 (47.61–91.44)	219.20 (164.40–294.85)	81.28 (60.79–109.59)	1.08 (0.95 to 1.21)
Morocco	605.74 (376.96–896.19)	16.21 (10.26–23.71)	2365.74 (1542.01–3379.26)	25.91 (17.17–36.52)	1.53 (1.47 to 1.58)
Mozambique	152.83 (103.62–218.39)	11.08 (7.77–15.46)	463.75 (336.39–645.84)	17.62 (13.09–24.02)	1.62 (1.57 to 1.66)
Myanmar	366.79 (214.79–571.44)	5.90 (3.56–9.01)	1210.06 (744.62–1814.75)	9.05 (5.71–13.31)	1.56 (1.48 to 1.63)
Namibia	28.41 (18.76–40.44)	16.71 (11.30–23.37)	92.35 (62.15–128.84)	25.11 (17.30–34.33)	1.32 (1.23 to 1.42)
Nauru	0.24 (0.15–0.35)	20.23 (13.07–29.09)	0.39 (0.24–0.56)	25.66 (16.66–36.36)	0.66 (0.47 to 0.86)
Nepal	399.96 (262.92–578.49)	17.56 (11.85–24.88)	1575.91 (1050.09–2216.81)	26.61 (18.10–36.96)	1.3 (1.24 to 1.35)
Netherlands	3544.66 (2371.48–5288.31)	64.13 (42.57–96.21)	7177.89 (4755.56–10298.28)	73.25 (47.80–106.21)	0.39 (0.27 to 0.5)
New Zealand	1320.87 (851.00–1864.93)	119.50 (76.47–169.75)	1797.44 (1256.33–2414.96)	75.04 (52.20–101.41)	-0.77 (-1.21 to -0.33)
Nicaragua	88.79 (57.67–127.65)	22.12 (14.69–31.23)	421.18 (272.88–608.79)	32.88 (21.56–46.98)	1.44 (1.31 to 1.56)
Niger	57.97 (35.95–87.01)	8.49 (5.50–12.32)	238.89 (153.89–349.96)	11.60 (7.72–16.62)	1.12 (1.08 to 1.15)
Nigeria	1724.75 (1070.11–2574.13)	15.11 (9.60–22.21)	4125.50 (2661.68–5983.12)	18.13 (12.07–25.60)	0.73 (0.59 to 0.86)
Niue	0.11 (0.07–0.17)	18.55 (11.70–27.15)	0.17 (0.11–0.25)	27.80 (17.81–40.60)	1.35 (1.3 to 1.39)
North Macedonia	144.54 (96.16–211.87)	28.73 (19.32–41.75)	286.46 (191.88–418.54)	31.59 (21.28–46.02)	0.28 (0.17 to 0.38)
Northern Mariana Islands	1.42 (0.86–2.16)	33.16 (21.11–48.30)	5.13 (3.28–7.50)	34.48 (22.56–49.49)	-0.17 (-0.29 to -0.04)
Norway	1567.27 (1055.25–2213.79)	82.10 (54.09–118.08)	1927.75 (1321.51–2680.33)	69.27 (46.64–97.76)	-0.71 (-0.82 to -0.61)
Oman	42.33 (25.76–63.67)	23.93 (15.16–34.82)	230.35 (142.64–344.27)	45.57 (29.81–64.81)	2.1 (2.04 to 2.16)
Pakistan	3150.30 (2047.35–4537.66)	21.78 (14.41–30.98)	5382.74 (3665.90–7540.31)	18.38 (12.94–25.10)	-0.48 (-0.56 to -0.39)
Palau	0.46 (0.28–0.67)	17.75 (11.19–25.94)	1.66 (1.05–2.42)	25.69 (16.54–36.85)	1.09 (1.02 to 1.15)
Palestine	45.79 (29.29–66.39)	20.64 (13.45–29.52)	216.47 (139.50–316.00)	34.09 (22.58–48.37)	1.54 (1.47 to 1.62)
Panama	149.61 (101.03–210.50)	38.00 (25.89–53.02)	590.71 (388.13–825.34)	48.67 (31.94–67.99)	0.72 (0.65 to 0.78)
Papua New Guinea	36.39 (20.49–57.98)	7.01 (4.13–10.84)	120.88 (73.11–182.53)	8.20 (5.21–11.94)	0.39 (0.3 to 0.48)
Paraguay	106.78 (71.73–156.05)	18.27 (12.45–26.43)	390.15 (268.99–565.79)	25.18 (17.55–36.23)	1.22 (1.17 to 1.28)

Peru	521.03 (340.46–739.48)	16.63 (11.04–23.27)	1912.16 (1189.60–2823.16)	21.03 (13.12–30.92)	0.81 (0.76 to 0.87)
Philippines	893.38 (533.73–1369.98)	11.55 (7.17–17.27)	2160.79 (1344.03–3220.34)	9.88 (6.39–14.32)	-0.52 (-0.55 to -0.49)
Poland	3649.66 (2377.41–5333.08)	30.93 (20.10–45.30)	5459.85 (4342.02–6870.48)	27.60 (21.71–35.04)	-1.44 (-2.16 to -0.72)
Portugal	1550.58 (1081.96–2154.70)	40.30 (28.04–56.39)	5134.53 (3903.11–6852.90)	71.62 (53.13–96.90)	1.7 (1.39 to 2.01)
Puerto Rico	302.33 (195.78–440.85)	30.70 (19.89–44.84)	607.24 (385.73–878.20)	31.55 (19.55–46.61)	-0.07 (-0.17 to 0.03)
Qatar	15.86 (9.19–25.14)	43.66 (28.09–63.52)	251.38 (150.21–389.22)	92.95 (63.18–130.13)	2.48 (2.32 to 2.64)
Republic of Korea	5492.82 (3321.67–8497.11)	61.10 (38.21–92.24)	28772.79 (18501.42–41423.03)	111.60 (71.52–161.24)	2.07 (1.78 to 2.36)
Republic of Moldova	635.09 (430.70–865.27)	52.62 (36.23–71.11)	1733.31 (1199.64–2391.11)	104.91 (72.62–144.63)	2.73 (2.55 to 2.91)
Romania	2676.49 (1796.75–3869.92)	34.80 (23.43–50.20)	6544.69 (4531.80–9545.10)	63.74 (43.79–93.11)	2.39 (2.19 to 2.6)
Russian Federation	55846.80 (39269.43–74625.28)	113.30 (80.04–150.88)	96176.75 (72103.21–122353.62)	144.80 (108.46–184.42)	1.17 (1.03 to 1.31)
Rwanda	76.30 (50.89–109.52)	12.46 (8.51–17.57)	210.31 (140.36–303.13)	14.45 (9.96–20.36)	0.46 (0.31 to 0.61)
Saint Kitts and Nevis	3.62 (2.43–5.14)	36.00 (23.94–51.52)	8.44 (5.55–12.37)	44.75 (30.01–64.68)	0.63 (0.57 to 0.69)
Saint Lucia	5.41 (3.50–7.80)	23.54 (15.34–33.84)	18.39 (11.99–26.68)	27.62 (18.07–39.96)	0.41 (0.36 to 0.47)
Saint Vincent and the Grenadines	2.65 (1.61–4.00)	14.02 (8.50–21.25)	6.14 (3.84–9.10)	15.49 (9.74–22.86)	0.38 (0.26 to 0.5)
Samoa	2.95 (1.83–4.43)	13.17 (8.37–19.46)	6.96 (4.41–10.11)	17.78 (11.45–25.49)	0.99 (0.96 to 1.03)
San Marino	3.46 (2.06–5.26)	36.28 (21.36–55.85)	7.80 (4.85–11.62)	38.88 (23.63–58.88)	0.27 (0.06 to 0.47)
Sao Tome and Principe	1.61 (1.01–2.40)	9.51 (6.06–14.01)	3.79 (2.35–5.68)	13.24 (8.51–19.31)	1.05 (0.95 to 1.16)
Saudi Arabia	388.49 (242.72–576.28)	25.99 (16.86–37.40)	2178.82 (1358.10–3256.18)	44.13 (29.02–62.75)	1.73 (1.69 to 1.77)
Senegal	84.45 (53.57–124.74)	10.34 (6.72–15.00)	283.44 (182.87–411.03)	14.22 (9.38–20.29)	0.94 (0.88 to 1)
Serbia	1258.88 (868.92–1800.54)	41.73 (29.10–59.03)	2467.69 (1826.64–3418.33)	53.42 (39.05–74.55)	0.63 (0.53 to 0.73)
Seychelles	2.15 (1.37–3.12)	14.03 (8.93–20.45)	6.46 (4.12–9.45)	20.87 (13.53–30.18)	1.06 (0.89 to 1.24)
Sierra Leone	41.27 (25.62–61.32)	7.80 (4.93–11.48)	108.92 (69.85–157.51)	11.23 (7.41–15.92)	1.28 (1.19 to 1.36)
Singapore	500.45 (309.19–748.57)	76.68 (48.68–112.33)	2899.67 (1936.51–4027.38)	124.52 (83.36–172.71)	1.75 (1.48 to 2.02)
Slovakia	293.75 (186.03–442.29)	18.13 (11.44–27.40)	810.98 (556.74–1165.63)	30.95 (21.10–44.77)	1.3 (1.04 to 1.55)

Slovenia	402.01 (284.98–563.05)	59.45 (42.04–83.59)	854.81 (608.78–1190.84)	66.33 (46.28–93.75)	0.21 (0.03 to 0.38)
Solomon Islands	3.41 (2.03–5.23)	8.94 (5.52–13.37)	11.30 (7.07–16.66)	11.82 (7.66–16.91)	0.74 (0.67 to 0.8)
Somalia	41.34 (26.67–61.34)	8.25 (5.60–11.70)	102.09 (68.88–145.97)	8.16 (5.83–11.17)	-0.06 (-0.09 to -0.04)
South Africa	977.20 (617.51–1433.25)	17.64 (11.36–25.49)	2500.15 (1699.64–3484.64)	20.13 (13.97–27.60)	0.55 (0.42 to 0.68)
South Sudan	77.41 (50.74–113.29)	12.49 (8.39–17.99)	115.22 (77.57–166.90)	13.15 (9.25–18.41)	0.19 (0.12 to 0.26)
Spain	12378.66 (8455.22–17745.19)	80.75 (54.96–116.25)	32072.07 (24312.52–41412.37)	117.27 (87.77–152.72)	1.19 (1.01 to 1.37)
Sri Lanka	292.15 (175.06–449.18)	10.36 (6.41–15.58)	1216.77 (754.25–1808.36)	16.44 (10.26–24.37)	1.58 (1.51 to 1.65)
Sudan	303.10 (185.23–459.27)	12.37 (7.73–18.47)	1042.49 (661.99–1535.49)	20.37 (13.33–29.21)	1.69 (1.64 to 1.75)
Suriname	10.22 (6.29–15.43)	14.46 (9.08–21.50)	29.61 (18.70–43.34)	16.86 (10.76–24.44)	0.6 (0.48 to 0.72)
Sweden	4073.37 (2664.40–5921.15)	93.67 (60.25–137.77)	3301.72 (2163.99–4832.76)	54.32 (34.61–81.01)	-1.74 (-2.08 to -1.4)
Switzerland	1373.23 (864.69–2020.41)	47.65 (29.54–70.85)	2895.30 (1937.40–4285.38)	57.67 (37.71–86.38)	1.08 (0.58 to 1.57)
Syrian Arab Republic	204.34 (124.17–310.36)	14.88 (9.36–22.01)	873.38 (542.94–1292.02)	24.07 (15.49–34.65)	1.6 (1.44 to 1.76)
Taiwan (Province of China)	1383.95 (869.06–2051.35)	32.53 (20.95–47.36)	9182.51 (7113.56–12268.44)	78.06 (60.51–104.51)	2.75 (2.02 to 3.49)
Tajikistan	109.54 (67.53–164.66)	14.80 (9.27–21.96)	301.94 (194.65–431.80)	20.66 (13.91–28.54)	1.23 (1.14 to 1.33)
Thailand	1294.66 (794.78–1943.71)	14.67 (9.28–21.47)	6422.51 (4079.38–9448.39)	21.34 (13.59–31.34)	1.25 (1.23 to 1.26)
Timor-Leste	5.47 (3.16–8.64)	7.65 (4.75–11.51)	25.46 (16.34–36.97)	11.24 (7.35–16.13)	1.5 (1.35 to 1.64)
Togo	29.16 (18.00–43.46)	9.49 (6.11–13.72)	132.63 (85.32–196.01)	13.74 (9.17–19.65)	1.22 (1.14 to 1.3)
Tokelau	0.04 (0.03–0.06)	11.78 (7.57–17.20)	0.08 (0.05–0.11)	19.39 (12.71–27.70)	1.65 (1.62 to 1.68)
Tonga	2.49 (1.57–3.66)	16.86 (10.86–24.51)	4.97 (3.24–7.11)	22.76 (14.94–32.38)	0.83 (0.78 to 0.88)
Trinidad and Tobago	57.23 (37.16–82.63)	25.78 (16.88–36.99)	135.49 (86.07–195.81)	25.74 (16.35–37.27)	0.06 (-0.01 to 0.13)
Tunisia	252.09 (155.86–375.04)	19.08 (12.08–27.93)	1182.97 (752.99–1703.27)	32.97 (21.21–47.07)	1.84 (1.76 to 1.91)
Turkey	2003.55 (1293.16–2909.04)	22.00 (14.47–31.51)	9192.68 (5962.88–13340.38)	36.10 (23.57–52.12)	1.11 (0.78 to 1.44)
Turkmenistan	93.42 (58.81–138.76)	18.35 (11.82–26.75)	308.70 (206.58–432.13)	28.89 (19.84–39.66)	1.68 (1.59 to 1.77)
Tuvalu	0.22 (0.14–0.32)	12.28 (7.95–17.83)	0.53 (0.34–0.76)	18.49 (12.14–26.53)	1.15 (1.09 to 1.21)

Uganda	186.16 (124.74–267.49)	12.34 (8.47–17.44)	559.59 (387.32–800.98)	16.26 (11.55–22.78)	0.94 (0.91 to 0.98)
Ukraine	24971.03 (17178.03–33784.33)	126.06 (86.84–170.26)	22554.32 (16123.95–29374.71)	102.49 (72.93–133.98)	-0.68 (-0.8 to -0.55)
United Arab Emirates	36.41 (21.52–56.48)	29.72 (19.06–43.27)	601.87 (362.17–929.43)	41.50 (27.57–58.60)	0.75 (0.54 to 0.95)
United Kingdom	20685.73 (13464.30–29877.77)	82.84 (53.08–121.31)	27566.78 (19891.74–37594.22)	76.93 (54.39–106.51)	0.34 (-0.07 to 0.75)
United Republic of Tanzania	283.23 (184.56–415.05)	11.03 (7.42–15.84)	964.33 (643.89–1394.75)	15.49 (10.62–21.97)	1.09 (1.05 to 1.13)
United States Virgin Islands	6.45 (4.04–9.59)	28.35 (18.13–41.39)	17.18 (11.12–24.54)	33.92 (21.74–49.09)	0.68 (0.58 to 0.78)
United States of America	163855.83 (118987.59–211899.92)	185.21 (132.99–242.30)	249218.59 (193440.43–306991.49)	153.95 (118.61–191.41)	-0.66 (-0.8 to -0.52)
Uruguay	1058.59 (714.54–1479.54)	97.10 (65.40–135.97)	1790.38 (1284.65–2425.18)	113.65 (80.45–155.63)	0.46 (0.37 to 0.54)
Uzbekistan	512.65 (306.56–778.74)	16.48 (9.99–24.74)	1559.77 (979.77–2277.24)	23.04 (15.08–32.51)	1.32 (1.23 to 1.42)
Vanuatu	1.66 (1.01–2.50)	10.23 (6.47–14.98)	6.31 (4.09–9.03)	13.52 (9.07–18.84)	0.89 (0.86 to 0.93)
Venezuela (Bolivarian Republic of)	1170.87 (794.35–1626.05)	47.41 (32.59–65.01)	4658.96 (3069.90–6714.10)	57.91 (38.44–82.99)	0.8 (0.67 to 0.94)
Viet Nam	1469.41 (945.50–2137.57)	14.48 (9.46–20.90)	4771.88 (3029.99–7049.36)	18.94 (12.34–27.53)	0.83 (0.75 to 0.9)
Yemen	184.31 (113.59–276.78)	14.90 (9.49–21.85)	689.17 (448.60–996.66)	19.06 (12.77–26.89)	0.99 (0.83 to 1.14)
Zambia	85.70 (57.60–122.94)	13.34 (9.26–18.66)	256.46 (174.37–371.12)	16.14 (11.39–22.74)	0.53 (0.48 to 0.58)
Zimbabwe	150.85 (91.92–230.06)	13.97 (8.84–20.74)	243.12 (155.72–351.41)	13.82 (9.30–19.31)	-0.37 (-0.51 to -0.23)

Abbreviations: VID, vascular intestinal diseases; ASR, age-standardized rate (per 100,000 population); EAPC, estimated annual percentage change; UI, uncertainty interval; CI, confidence interval.

Table S2. Global and regional prevalence of middle-aged and elderly VID in 1990 and 2021, and EAPC of ASR from 1990 to 2021.

Table S2. Global and regional prevalence of middle-aged and elderly VID in 1990 and 2021, and EAPC of ASR from 1990 to 2021.					
**Location**	**1990**		**2021**		**EAPC, 1990-2021**
	**Number**	**ASR**	**Number**	**ASR**	
Afghanistan	20.52 (13.48–29.43)	1.08 (0.72–1.53)	38.57 (25.68–54.69)	1.41 (1.01–1.89)	1.02 (0.85 to 1.19)
Albania	11.46 (7.11–17.39)	2.03 (1.31–3.00)	38.81 (26.68–54.52)	3.24 (2.20–4.60)	1.78 (1.47 to 2.1)
Algeria	64.68 (42.42–92.55)	2.00 (1.34–2.82)	312.72 (216.21–434.25)	3.30 (2.34–4.49)	1.66 (1.61 to 1.71)
American Samoa	0.10 (0.07–0.15)	1.67 (1.13–2.37)	0.27 (0.19–0.37)	1.99 (1.42–2.70)	0.47 (0.39 to 0.56)
Andorra	1.56 (1.13–2.09)	10.03 (7.27–13.40)	6.56 (5.01–8.41)	15.37 (11.74–19.70)	1.3 (1.16 to 1.45)
Angola	12.70 (8.78–17.59)	1.29 (0.92–1.73)	66.61 (49.69–87.59)	2.25 (1.73–2.88)	1.94 (1.73 to 2.14)
Antigua and Barbuda	0.28 (0.19–0.40)	2.02 (1.33–2.93)	0.74 (0.50–1.06)	2.48 (1.70–3.48)	0.57 (0.44 to 0.69)
Argentina	601.98 (474.17–751.58)	6.79 (5.34–8.50)	1282.75 (1042.65–1565.05)	8.29 (6.70–10.17)	0.61 (0.52 to 0.71)
Armenia	50.68 (39.99–63.40)	7.01 (5.56–8.72)	144.92 (119.58–174.35)	12.12 (9.96–14.65)	2.08 (1.98 to 2.18)
Australia	545.40 (430.76–682.34)	10.01 (7.84–12.62)	1499.43 (1195.51–1859.39)	11.56 (9.05–14.57)	0.29 (0.15 to 0.42)
Austria	336.98 (273.59–411.55)	9.70 (7.77–12.00)	488.62 (387.75–609.22)	9.56 (7.44–12.14)	-0.35 (-0.73 to 0.03)
Azerbaijan	23.00 (14.91–33.72)	1.63 (1.09–2.36)	70.91 (48.77–99.39)	2.50 (1.78–3.43)	1.67 (1.48 to 1.87)
Bahamas	1.87 (1.41–2.43)	4.48 (3.41–5.77)	5.80 (4.47–7.41)	5.08 (3.97–6.42)	0.47 (0.41 to 0.53)
Bahrain	2.54 (1.87–3.39)	7.43 (5.69–9.54)	15.28 (10.64–21.15)	8.54 (6.62–10.84)	-0.24 (-0.55 to 0.07)
Bangladesh	207.21 (146.58–282.49)	1.67 (1.20–2.24)	1264.60 (948.38–1657.91)	3.36 (2.54–4.38)	2.48 (2.41 to 2.55)
Barbados	1.78 (1.28–2.43)	2.35 (1.62–3.29)	4.21 (3.03–5.71)	2.97 (2.11–4.07)	0.6 (0.52 to 0.68)
Belarus	248.24 (191.72–315.13)	6.98 (5.38–8.87)	696.84 (571.23–841.70)	15.52 (12.68–18.82)	3.03 (2.87 to 3.2)
Belgium	618.37 (498.71–754.20)	14.14 (11.27–17.44)	1378.42 (1146.08–1651.59)	20.81 (17.08–25.23)	0.94 (0.69 to 1.2)
Belize	0.36 (0.23–0.54)	1.44 (0.91–2.16)	1.58 (1.06–2.26)	1.89 (1.30–2.64)	0.86 (0.68 to 1.05)
Benin	4.02 (2.58–5.90)	0.76 (0.50–1.10)	17.19 (12.02–23.67)	1.26 (0.92–1.69)	1.61 (1.55 to 1.66)
Bermuda	0.76 (0.56–1.01)	4.53 (3.34–6.00)	2.02 (1.51–2.63)	5.36 (3.92–7.09)	0.2 (0.03 to 0.36)
Bhutan	0.91 (0.60–1.30)	1.44 (0.98–1.99)	5.89 (4.28–7.94)	3.69 (2.70–4.94)	3.29 (3.21 to 3.37)
Bolivia (Plurinational State of)	13.74 (9.63–18.82)	1.67 (1.19–2.25)	70.56 (52.90–92.15)	2.98 (2.26–3.85)	2 (1.96 to 2.04)
Bosnia and Herzegovina	30.76 (21.25–42.67)	2.89 (2.07–3.91)	86.24 (66.54–110.22)	4.96 (3.78–6.42)	2.32 (1.93 to 2.72)
Botswana	2.86 (1.94–4.00)	1.87 (1.30–2.59)	11.75 (8.63–15.52)	2.91 (2.19–3.78)	1.26 (1.16 to 1.37)

Brazil	1587.51 (1275.78–1944.03)	6.56 (5.32–7.98)	3194.34 (2581.01–3899.17)	4.62 (3.74–5.63)	-0.77 (-0.92 to -0.63)
Brunei Darussalam	2.16 (1.44–3.07)	7.49 (5.23–10.34)	9.31 (6.80–12.44)	9.84 (7.50–12.69)	0.83 (0.64 to 1.02)
Bulgaria	142.12 (106.40–186.13)	4.09 (3.05–5.37)	293.28 (238.98–355.44)	7.22 (5.79–8.88)	2.38 (2.15 to 2.61)
Burkina Faso	8.90 (5.75–13.14)	0.79 (0.53–1.14)	32.44 (23.18–43.86)	1.34 (0.98–1.79)	2 (1.9 to 2.1)
Burundi	6.44 (4.44–8.82)	1.10 (0.77–1.50)	20.13 (14.84–26.47)	1.64 (1.24–2.13)	1.36 (1.25 to 1.47)
Cabo Verde	0.60 (0.40–0.86)	0.96 (0.63–1.41)	2.39 (1.67–3.30)	2.05 (1.47–2.77)	2.5 (2.41 to 2.59)
Cambodia	8.30 (5.24–12.35)	0.73 (0.48–1.05)	43.42 (30.30–60.10)	1.41 (1.02–1.89)	2.36 (2.29 to 2.43)
Cameroon	10.51 (6.73–15.48)	0.89 (0.60–1.28)	48.48 (33.67–66.71)	1.49 (1.07–2.00)	1.62 (1.54 to 1.71)
Canada	1441.06 (1136.99–1800.02)	16.17 (12.64–20.37)	3731.64 (3038.65–4522.85)	18.53 (14.86–22.78)	0.52 (0.4 to 0.64)
Central African Republic	4.83 (3.41–6.58)	1.62 (1.17–2.17)	9.95 (7.45–13.03)	1.72 (1.32–2.20)	0.1 (0.05 to 0.15)
Chad	6.35 (4.29–8.99)	0.84 (0.58–1.19)	18.79 (13.53–25.40)	1.23 (0.92–1.63)	1.27 (1.24 to 1.3)
Chile	196.51 (155.70–245.12)	7.37 (5.87–9.14)	886.66 (723.08–1077.40)	12.46 (10.14–15.18)	1.78 (1.69 to 1.86)
China	4118.58 (2562.92–6232.11)	1.90 (1.23–2.80)	10227.25 (7080.04–14377.55)	1.73 (1.21–2.41)	-0.28 (-0.34 to -0.23)
Colombia	218.09 (169.65–276.94)	4.73 (3.74–5.92)	1361.12 (1089.69–1671.63)	9.07 (7.27–11.13)	2.08 (1.92 to 2.25)
Comoros	0.78 (0.54–1.05)	1.55 (1.11–2.07)	2.73 (2.04–3.55)	2.13 (1.62–2.75)	1.02 (0.89 to 1.14)
Congo	5.23 (3.80–6.93)	1.93 (1.42–2.53)	18.73 (14.09–24.48)	2.75 (2.13–3.50)	1.15 (1.08 to 1.22)
Cook Islands	0.10 (0.07–0.13)	2.85 (2.06–3.82)	0.37 (0.29–0.47)	5.04 (3.87–6.45)	1.67 (1.6 to 1.74)
Costa Rica	41.97 (33.29–52.25)	9.23 (7.35–11.44)	222.46 (181.33–269.73)	15.01 (12.25–18.19)	1.54 (1.42 to 1.65)
Coted'Ivoire	9.42 (5.86–13.97)	0.89 (0.59–1.26)	39.59 (27.45–55.10)	1.36 (0.99–1.82)	1.2 (1.12 to 1.29)
Croatia	185.17 (150.08–227.74)	11.43 (9.31–14.00)	326.10 (269.39–388.59)	12.37 (10.09–14.94)	0.16 (0.04 to 0.27)
Cuba	175.94 (137.29–222.77)	6.26 (4.88–7.94)	526.82 (423.14–646.92)	9.68 (7.76–11.91)	1.4 (1.36 to 1.43)
Cyprus	11.43 (8.12–15.44)	5.33 (3.77–7.22)	47.21 (36.35–59.87)	8.09 (6.16–10.37)	1.48 (1.35 to 1.61)
Czechia	238.81 (187.59–300.69)	6.20 (4.82–7.88)	455.52 (359.12–569.36)	7.49 (5.77–9.55)	0.14 (-0.08 to 0.35)
Democratic People's Republic of Korea	66.45 (40.00–101.35)	1.47 (0.93–2.17)	186.31 (125.13–263.34)	2.03 (1.38–2.83)	1.19 (1.07 to 1.31)
Democratic Republic of the Congo	95.67 (66.71–130.50)	2.47 (1.74–3.34)	254.70 (190.32–330.22)	2.67 (2.03–3.42)	0.13 (-0.2 to 0.46)
Denmark	239.80 (191.30–294.84)	10.60 (8.34–13.18)	347.03 (276.42–427.91)	10.20 (7.95–12.82)	-0.55 (-0.87 to -0.22)
Djibouti	0.47 (0.32–0.66)	1.42 (1.01–1.93)	3.28 (2.39–4.38)	2.06 (1.56–2.68)	1.22 (1.07 to 1.36)
Dominica	0.64 (0.48–0.84)	3.97 (2.94–5.20)	1.02 (0.78–1.29)	4.43 (3.42–5.63)	0.3 (0.21 to 0.4)

Dominican Republic	15.76 (10.29–23.17)	1.55 (1.03–2.25)	68.77 (49.32–93.05)	2.51 (1.82–3.38)	1.55<br>(1.39 to 1.71)
Ecuador	28.55 (20.76–38.12)	2.06 (1.53–2.72)	198.74 (156.82–249.35)	4.52 (3.58–5.65)	2.9<br>(2.8 to 3)
Egypt	105.91 (64.97–160.12)	1.45 (0.94–2.11)	464.31 (309.75–662.83)	2.81 (1.96–3.87)	2.08<br>(1.99 to 2.17)
El Salvador	14.43 (9.90–20.33)	1.80 (1.25–2.51)	65.57 (49.14–85.67)	3.88 (2.90–5.08)	2.62<br>(2.36 to 2.89)
Equatorial Guinea	0.72 (0.49–0.98)	1.41 (0.99–1.91)	5.09 (3.77–6.69)	4.11 (3.10–5.33)	4.03<br>(3.78 to 4.28)
Eritrea	2.68 (1.80–3.73)	0.98 (0.69–1.31)	11.52 (8.63–14.92)	1.71 (1.32–2.16)	1.67<br>(1.52 to 1.81)
Estonia	82.28 (67.18–99.04)	14.32 (11.66–<br>17.27)	170.73 (142.69–202.94)	21.22 (17.55–<br>25.47)	1.26<br>(1.03 to 1.49)
Eswatini	0.93 (0.58–1.39)	1.14 (0.74–1.65)	2.60 (1.87–3.51)	1.70 (1.26–2.25)	1.28<br>(1.22 to 1.35)
Ethiopia	55.83 (39.31–75.67)	1.16 (0.84–1.54)	161.78 (121.90–210.28)	1.51 (1.16–1.93)	1.15<br>(0.99 to 1.32)
Fiji	1.26 (0.80–1.87)	1.27 (0.85–1.82)	3.48 (2.44–4.80)	1.62 (1.16–2.19)	0.67<br>(0.62 to 0.72)
Finland	205.49 (162.29–257.63)	10.10 (7.88–<br>12.79)	480.67 (392.28–582.97)	12.86 (10.21–<br>15.98)	0.66<br>(0.5 to 0.82)
France	2754.25 (2231.75–3346.41)	11.58 (9.25–<br>14.27)	5731.15 (4702.77–6897.95)	14.61 (11.76–<br>17.91)	0.81<br>(0.7 to 0.92)
Gabon	3.39 (2.42–4.57)	2.26 (1.62–3.03)	9.61 (7.30–12.35)	3.56 (2.75–4.53)	1.38<br>(1.33 to 1.42)
Gambia	0.94 (0.62–1.36)	1.04 (0.70–1.46)	3.41 (2.42–4.68)	1.34 (0.98–1.80)	0.66<br>(0.58 to 0.74)
Georgia	34.72 (21.80–51.78)	2.02 (1.28–2.98)	61.35 (48.23–77.31)	3.77 (2.93–4.79)	2.44<br>(2.16 to 2.72)
Germany	4905.47 (3936.17–6014.45)	13.46 (10.70–<br>16.65)	8960.25 (7332.41–<br>10831.57)	16.39 (13.17–<br>20.14)	0.23<br>(-0.08 to 0.54)
Ghana	16.95 (10.88–24.75)	1.04 (0.69–1.48)	67.31 (47.35–93.04)	1.55 (1.13–2.08)	1.12<br>(0.97 to 1.26)
Greece	294.80 (215.73–391.06)	6.93 (5.01–9.28)	538.62 (422.27–676.61)	7.97 (6.03–10.32)	0.55<br>(0.36 to 0.73)
Greenland	1.07 (0.82–1.38)	12.52 (9.65–<br>15.84)	3.37 (2.75–4.10)	17.57 (14.32–<br>21.36)	1.13<br>(1.1 to 1.16)
Grenada	0.30 (0.20–0.42)	1.60 (1.04–2.32)	0.84 (0.59–1.16)	2.62 (1.88–3.55)	1.43<br>(1.27 to 1.58)
Guam	0.43 (0.27–0.63)	2.20 (1.48–3.12)	1.45 (1.03–1.99)	2.49 (1.77–3.40)	0.52<br>(0.46 to 0.58)
Guatemala	12.82 (8.20–18.87)	1.37 (0.92–1.95)	59.89 (41.77–82.89)	2.02 (1.43–2.76)	1.19<br>(0.93 to 1.45)
Guinea	6.41 (4.05–9.46)	0.73 (0.47–1.06)	15.77 (10.92–21.73)	1.06 (0.75–1.43)	1.11<br>(1.01 to 1.21)
Guinea-Bissau	0.93 (0.62–1.31)	0.90 (0.62–1.24)	2.51 (1.82–3.37)	1.35 (1.02–1.75)	1.32<br>(1.27 to 1.37)
Guyana	1.99 (1.42–2.70)	1.94 (1.41–2.60)	4.73 (3.53–6.20)	2.60 (1.97–3.37)	0.94<br>(0.84 to 1.04)
Haiti	11.70 (8.15–16.13)	1.38 (0.98–1.88)	28.04 (19.93–38.09)	1.45 (1.06–1.92)	0.25<br>(0.2 to 0.29)
Honduras	23.31 (17.46–30.38)	4.44 (3.35–5.76)	150.33 (119.54–187.10)	9.30 (7.44–11.53)	2.51<br>(2.42 to 2.59)
Hungary	408.39 (334.91–491.38)	9.99 (8.14–12.08)	765.12 (636.15–909.53)	14.09 (11.59–<br>16.90)	0.71<br>(0.53 to 0.9)

Iceland	8.95 (6.90–11.43)	11.37 (8.67–14.69)	16.51 (12.61–21.23)	10.38 (7.79–13.56)	-0.58<br>(-0.9 to -0.26)
India	1880.51 (1240.82–2695.30)	1.55 (1.06–2.16)	5252.17 (3790.40–7049.59)	1.65 (1.21–2.18)	0.21<br>(0.12 to 0.3)
Indonesia	326.50 (214.19–472.05)	1.37 (0.95–1.91)	956.29 (692.40–1291.75)	1.71 (1.31–2.22)	0.73<br>(0.66 to 0.8)
Iran (Islamic Republic of)	183.19 (115.56–272.52)	2.77 (1.82–3.98)	506.36 (348.97–707.73)	2.45 (1.75–3.34)	-0.46<br>(-0.53 to -0.38)
Iraq	29.18 (17.62–44.39)	1.35 (0.83–2.02)	148.31 (93.78–219.21)	2.18 (1.46–3.10)	1.87<br>(1.75 to 1.99)
Ireland	109.42 (86.53–137.17)	9.40 (7.33–11.93)	331.97 (267.45–408.49)	14.88 (11.88–18.48)	1.94<br>(1.77 to 2.11)
Israel	115.80 (91.25–145.30)	8.51 (6.62–10.81)	407.91 (326.20–505.60)	11.73 (9.27–14.70)	0.9<br>(0.73 to 1.06)
Italy	3911.49 (3127.95–4838.95)	15.37 (12.16–19.25)	4063.57 (3325.11–4947.08)	9.39 (7.49–11.70)	-1.94<br>(-2.07 to -1.8)
Jamaica	10.73 (7.46–14.89)	2.26 (1.54–3.17)	21.05 (14.98–28.56)	2.50 (1.78–3.39)	0.41<br>(0.34 to 0.48)
Japan	5440.30 (3723.78–7615.64)	11.56 (7.92–16.18)	9834.24 (7511.55–12571.54)	11.15 (7.93–15.08)	0.14<br>(-0.02 to 0.31)
Jordan	10.26 (7.12–14.15)	3.09 (2.22–4.15)	80.97 (57.65–110.98)	4.18 (3.12–5.52)	1.08<br>(1.01 to 1.16)
Kazakhstan	147.75 (112.09–191.17)	4.40 (3.37–5.65)	298.06 (234.59–374.30)	6.12 (4.86–7.61)	1.36<br>(1.18 to 1.53)
Kenya	46.87 (31.46–66.20)	2.31 (1.57–3.23)	152.28 (111.99–201.21)	2.71 (2.02–3.54)	0.51<br>(0.48 to 0.54)
Kiribati	0.24 (0.18–0.32)	2.35 (1.74–3.08)	0.58 (0.45–0.73)	2.78 (2.17–3.49)	0.35<br>(0.28 to 0.42)
Kuwait	6.87 (4.69–9.66)	4.64 (3.39–6.18)	52.71 (36.99–72.46)	7.33 (5.59–9.45)	1.92<br>(1.79 to 2.05)
Kyrgyzstan	13.81 (9.20–19.67)	1.70 (1.15–2.41)	26.82 (17.87–38.15)	2.04 (1.42–2.80)	0.51<br>(0.42 to 0.6)
Lao People's Democratic Republic	3.37 (2.08–5.05)	0.62 (0.40–0.91)	13.54 (9.11–19.16)	1.13 (0.80–1.55)	2.09<br>(2 to 2.18)
Latvia	148.07 (122.18–178.75)	14.74 (12.13–17.82)	225.77 (188.90–267.10)	18.99 (15.76–22.66)	0.49<br>(0.39 to 0.59)
Lebanon	12.61 (8.07–18.37)	2.19 (1.45–3.12)	73.69 (53.01–99.73)	4.44 (3.16–6.06)	2.45<br>(2.38 to 2.52)
Lesotho	2.24 (1.44–3.30)	0.97 (0.63–1.40)	3.70 (2.67–4.98)	1.28 (0.94–1.69)	0.83<br>(0.79 to 0.88)
Liberia	2.20 (1.41–3.25)	0.73 (0.47–1.07)	6.76 (4.57–9.55)	1.26 (0.90–1.70)	2.03<br>(1.94 to 2.13)
Libya	11.01 (6.96–16.29)	2.20 (1.43–3.18)	42.16 (28.37–60.57)	3.02 (2.15–4.16)	1.06<br>(0.88 to 1.23)
Lithuania	118.73 (95.22–147.75)	9.50 (7.61–11.84)	335.88 (281.58–395.83)	19.42 (16.15–23.06)	2.41<br>(2.25 to 2.56)
Luxembourg	22.58 (18.07–27.89)	14.58 (11.60–18.13)	51.97 (41.57–63.87)	17.54 (13.95–21.66)	0.29<br>(-0.02 to 0.59)
Madagascar	12.13 (7.97–17.45)	0.96 (0.65–1.35)	41.50 (30.14–55.50)	1.49 (1.13–1.93)	1.45<br>(1.4 to 1.49)
Malawi	10.79 (7.44–14.80)	1.16 (0.82–1.56)	35.67 (26.50–46.70)	1.93 (1.46–2.50)	1.74<br>(1.65 to 1.82)
Malaysia	41.85 (28.49–59.20)	1.79 (1.26–2.46)	349.16 (266.20–448.00)	4.83 (3.73–6.14)	3.46<br>(3.24 to 3.68)
Maldives	0.26 (0.16–0.39)	1.11 (0.73–1.61)	2.02 (1.38–2.88)	2.47 (1.78–3.37)	2.59<br>(2.39 to 2.79)

Mali	7.29 (4.45–10.92)	0.69 (0.44–1.01)	28.75 (19.98–39.68)	1.23 (0.89–1.66)	2.02 (1.93 to 2.1)
Malta	7.98 (6.17–10.19)	6.77 (5.22–8.67)	24.67 (19.55–30.34)	8.69 (6.71–10.94)	0.43 (0.19 to 0.67)
Marshall Islands	0.06 (0.04–0.08)	1.33 (0.92–1.85)	0.17 (0.12–0.23)	1.73 (1.28–2.30)	0.75 (0.69 to 0.8)
Mauritania	2.50 (1.68–3.55)	0.94 (0.64–1.33)	8.76 (6.12–12.07)	1.54 (1.10–2.09)	1.32 (1.22 to 1.43)
Mauritius	2.39 (1.49–3.59)	1.26 (0.82–1.84)	9.42 (6.49–13.18)	1.88 (1.31–2.61)	1.06 (0.9 to 1.21)
Mexico	777.32 (610.94–976.79)	7.01 (5.59–8.70)	1976.63 (1609.39–2411.45)	5.84 (4.79–7.08)	-0.7 (-0.79 to -0.62)
Micronesia (Federated States of)	0.16 (0.11–0.23)	1.26 (0.87–1.76)	0.43 (0.31–0.58)	2.08 (1.54–2.76)	1.69 (1.64 to 1.73)
Monaco	1.96 (1.48–2.55)	9.84 (7.25–13.09)	3.43 (2.71–4.30)	12.38 (9.59–15.85)	0.74 (0.61 to 0.87)
Mongolia	3.92 (2.59–5.65)	1.36 (0.92–1.93)	13.85 (9.35–19.65)	2.13 (1.52–2.91)	1.71 (1.55 to 1.87)
Montenegro	27.38 (21.45–34.56)	16.09 (12.62–20.28)	58.97 (48.39–71.10)	21.48 (17.57–25.98)	1.45 (1.3 to 1.61)
Morocco	56.08 (35.63–82.60)	1.47 (0.95–2.14)	233.11 (162.22–321.84)	2.53 (1.80–3.44)	1.77 (1.71 to 1.82)
Mozambique	18.04 (12.75–24.53)	1.20 (0.87–1.60)	70.58 (55.13–89.49)	2.44 (1.93–3.06)	2.42 (2.36 to 2.48)
Myanmar	33.81 (19.07–53.19)	0.53 (0.31–0.81)	121.66 (77.21–180.27)	0.90 (0.59–1.30)	1.96 (1.87 to 2.04)
Namibia	2.56 (1.74–3.58)	1.45 (1.00–1.99)	9.03 (6.67–11.98)	2.39 (1.81–3.12)	1.65 (1.54 to 1.76)
Nauru	0.03 (0.02–0.04)	2.13 (1.48–2.95)	0.05 (0.03–0.06)	3.05 (2.29–3.98)	1.02 (0.77 to 1.27)
Nepal	38.45 (26.78–52.99)	1.62 (1.15–2.19)	189.35 (141.67–246.35)	3.15 (2.38–4.06)	2.16 (2.12 to 2.21)
Netherlands	663.57 (529.22–822.22)	11.85 (9.33–14.85)	1482.65 (1198.55–1805.61)	14.88 (11.84–18.39)	0.76 (0.56 to 0.95)
New Zealand	169.06 (132.93–211.40)	15.24 (11.87–19.24)	222.47 (178.63–276.18)	9.25 (7.35–11.61)	-1.57 (-1.79 to -1.34)
Nicaragua	8.62 (5.82–12.14)	2.10 (1.46–2.90)	49.35 (36.29–65.12)	3.81 (2.85–4.96)	2.14 (1.97 to 2.32)
Niger	5.44 (3.45–7.98)	0.75 (0.50–1.06)	25.33 (18.00–34.53)	1.17 (0.86–1.56)	1.65 (1.57 to 1.74)
Nigeria	175.10 (111.98–256.72)	1.50 (0.98–2.16)	418.89 (289.61–581.18)	1.79 (1.29–2.42)	0.7 (0.55 to 0.85)
Niue	0.01 (0.01–0.02)	2.06 (1.41–2.87)	0.02 (0.02–0.03)	3.55 (2.63–4.70)	1.76 (1.73 to 1.8)
North Macedonia	25.24 (19.13–32.67)	4.96 (3.79–6.40)	55.39 (42.75–70.30)	6.00 (4.62–7.62)	0.57 (0.33 to 0.81)
Northern Mariana Islands	0.17 (0.12–0.24)	4.09 (2.99–5.46)	0.69 (0.52–0.90)	4.63 (3.55–5.93)	0.01 (-0.14 to 0.17)
Norway	228.19 (181.49–284.88)	11.70 (9.03–15.02)	266.85 (215.24–328.78)	9.43 (7.43–11.86)	-1.19 (-1.38 to -1.01)
Oman	4.24 (2.66–6.32)	2.35 (1.56–3.37)	24.58 (16.35–35.34)	4.90 (3.55–6.59)	2.39 (2.32 to 2.47)
Pakistan	290.28 (195.77–407.64)	1.95 (1.34–2.71)	464.47 (331.75–624.70)	1.51 (1.12–1.98)	-0.75 (-0.85 to -0.65)
Palau	0.05 (0.03–0.07)	1.92 (1.31–2.72)	0.20 (0.14–0.27)	3.11 (2.28–4.14)	1.4 (1.32 to 1.49)

Palestine	4.56 (3.04–6.49)	2.03 (1.38–2.84)	23.17 (16.32–31.89)	3.65 (2.70–4.82)	1.83 (1.75 to 1.9)
Panama	15.41 (11.40–20.38)	3.88 (2.90–5.08)	71.25 (54.96–90.70)	5.89 (4.55–7.49)	1.17 (1.07 to 1.26)
Papua New Guinea	3.45 (1.88–5.56)	0.64 (0.37–1.00)	11.48 (7.09–17.32)	0.75 (0.49–1.09)	0.39 (0.29 to 0.49)
Paraguay	16.84 (12.08–22.61)	2.82 (2.05–3.76)	73.38 (56.67–93.28)	4.69 (3.64–5.93)	1.94 (1.86 to 2.01)
Peru	49.78 (34.41–69.52)	1.57 (1.11–2.15)	232.53 (164.24–319.73)	2.56 (1.82–3.50)	1.67 (1.62 to 1.73)
Philippines	91.91 (54.99–141.69)	1.16 (0.73–1.73)	214.98 (139.22–318.20)	0.97 (0.66–1.38)	-0.59 (-0.62 to -0.56)
Poland	522.97 (388.22–689.12)	4.39 (3.24–5.81)	1165.92 (965.34–1402.46)	5.87 (4.80–7.14)	0.03 (-0.66 to 0.72)
Portugal	263.02 (206.80–328.07)	6.72 (5.24–8.45)	1252.96 (1049.23–1483.77)	17.19 (14.17–20.66)	3.01 (2.66 to 3.37)
Puerto Rico	38.12 (28.12–50.70)	3.84 (2.82–5.13)	78.16 (58.24–103.29)	4.10 (2.94–5.58)	0 (-0.14 to 0.14)
Qatar	1.51 (0.87–2.36)	3.95 (2.58–5.69)	24.56 (15.29–37.53)	9.23 (6.78–12.34)	2.84 (2.71 to 2.98)
Republic of Korea	538.64 (322.81–827.76)	5.90 (3.67–8.86)	2948.56 (2057.73–4077.31)	11.45 (7.95–15.88)	2.27 (1.95 to 2.58)
Republic of Moldova	71.36 (56.27–88.65)	5.79 (4.57–7.19)	240.96 (202.71–284.72)	14.59 (12.24–17.30)	3.51 (3.32 to 3.71)
Romania	391.44 (304.56–495.86)	5.01 (3.88–6.36)	1262.92 (1048.09–1508.08)	12.27 (10.10–14.77)	3.32 (3.15 to 3.49)
Russian Federation	6308.93 (5137.43–7692.71)	12.66 (10.28–15.47)	10497.31 (9032.64–12050.18)	15.80 (13.55–18.21)	0.95 (0.81 to 1.09)
Rwanda	8.52 (5.94–11.67)	1.26 (0.89–1.70)	30.90 (22.34–41.25)	1.97 (1.45–2.59)	1.57 (1.36 to 1.78)
Saint Kitts and Nevis	0.48 (0.37–0.61)	4.64 (3.51–6.00)	1.39 (1.07–1.76)	7.31 (5.72–9.18)	1.28 (1.15 to 1.41)
Saint Lucia	0.63 (0.46–0.84)	2.68 (1.96–3.58)	2.40 (1.80–3.15)	3.59 (2.71–4.69)	0.74 (0.66 to 0.82)
Saint Vincent and the Grenadines	0.28 (0.18–0.42)	1.49 (0.94–2.23)	0.67 (0.44–0.98)	1.69 (1.12–2.45)	0.44 (0.27 to 0.6)
Samoa	0.30 (0.20–0.44)	1.32 (0.88–1.88)	0.78 (0.55–1.06)	1.97 (1.43–2.64)	1.27 (1.22 to 1.31)
San Marino	0.46 (0.30–0.67)	4.82 (3.06–7.17)	1.11 (0.78–1.53)	5.45 (3.69–7.77)	0.44 (0.17 to 0.7)
Sao Tome and Principe	0.15 (0.10–0.23)	0.89 (0.58–1.30)	0.43 (0.29–0.61)	1.48 (1.04–2.04)	1.62 (1.51 to 1.73)
Saudi Arabia	37.74 (24.42–54.63)	2.47 (1.67–3.46)	230.22 (149.85–334.52)	4.67 (3.31–6.33)	2.08 (2.04 to 2.12)
Senegal	7.97 (5.19–11.40)	0.94 (0.63–1.32)	30.75 (21.95–41.54)	1.50 (1.09–1.99)	1.39 (1.32 to 1.46)
Serbia	252.41 (195.97–318.50)	8.21 (6.39–10.34)	567.36 (467.72–682.63)	12.11 (9.89–14.71)	1.29 (1.13 to 1.45)
Seychelles	0.21 (0.14–0.30)	1.38 (0.93–1.97)	0.74 (0.53–1.02)	2.42 (1.76–3.25)	1.58 (1.36 to 1.8)
Sierra Leone	3.73 (2.34–5.56)	0.69 (0.44–1.01)	11.46 (8.01–15.78)	1.14 (0.82–1.54)	1.76 (1.62 to 1.89)
Singapore	49.30 (30.73–73.88)	7.48 (4.82–10.98)	292.10 (211.40–388.31)	12.59 (9.17–16.68)	1.89 (1.59 to 2.18)
Slovakia	47.95 (33.52–66.81)	2.93 (2.03–4.10)	179.32 (140.17–225.78)	6.76 (5.23–8.60)	2.85 (2.77 to 2.93)

Slovenia	83.48 (66.38–103.46)	12.21 (9.69–15.14)	200.01 (161.90–244.23)	15.26 (12.19–18.88)	0.81 (0.71 to 0.9)
Solomon Islands	0.34 (0.21–0.52)	0.85 (0.54–1.27)	1.18 (0.79–1.68)	1.21 (0.85–1.66)	0.93 (0.86 to 1.01)
Somalia	4.65 (3.07–6.57)	0.83 (0.58–1.13)	12.42 (8.89–16.77)	0.87 (0.65–1.14)	0.14 (0.12 to 0.16)
South Africa	95.02 (61.96–137.46)	1.68 (1.12–2.40)	243.81 (177.14–328.14)	1.93 (1.43–2.55)	0.6 (0.44 to 0.77)
South Sudan	8.28 (5.69–11.45)	1.26 (0.88–1.73)	14.93 (10.82–19.97)	1.57 (1.18–2.05)	0.67 (0.62 to 0.72)
Spain	2163.74 (1767.21–2626.24)	13.92 (11.26–17.05)	5903.13 (4895.74–7047.58)	20.40 (16.70–24.64)	0.97 (0.61 to 1.33)
Sri Lanka	29.52 (18.01–45.06)	1.02 (0.65–1.52)	142.40 (97.35–201.05)	1.93 (1.34–2.70)	2.18 (2.1 to 2.27)
Sudan	27.54 (16.64–41.49)	1.09 (0.67–1.62)	101.36 (66.67–146.30)	1.95 (1.33–2.73)	1.95 (1.88 to 2.02)
Suriname	1.03 (0.65–1.53)	1.44 (0.93–2.09)	3.13 (2.13–4.42)	1.76 (1.22–2.46)	0.79 (0.63 to 0.96)
Sweden	730.36 (584.93–902.03)	16.35 (12.77–20.66)	542.25 (430.56–673.77)	8.63 (6.65–11.05)	-2.37 (-2.49 to -2.24)
Switzerland	212.96 (159.72–281.33)	7.25 (5.31–9.76)	531.72 (417.47–670.03)	10.28 (7.88–13.22)	1.08 (0.74 to 1.43)
Syrian Arab Republic	19.54 (11.96–29.68)	1.39 (0.89–2.05)	89.96 (58.72–129.58)	2.46 (1.69–3.42)	1.88 (1.68 to 2.08)
Taiwan (Province of China)	134.82 (86.89–197.31)	3.15 (2.09–4.52)	1240.16 (993.83–1538.98)	10.60 (8.48–13.18)	4.03 (3.36 to 4.71)
Tajikistan	10.43 (6.56–15.59)	1.39 (0.89–2.05)	29.59 (20.38–41.31)	1.98 (1.44–2.65)	1.28 (1.18 to 1.38)
Thailand	134.30 (88.12–196.95)	1.50 (1.03–2.13)	812.94 (590.72–1089.84)	2.72 (1.99–3.63)	1.94 (1.89 to 1.98)
Timor-Leste	0.50 (0.28–0.79)	0.66 (0.41–0.99)	2.55 (1.76–3.53)	1.11 (0.78–1.52)	1.99 (1.81 to 2.17)
Togo	2.86 (1.81–4.19)	0.89 (0.59–1.26)	15.46 (11.03–21.16)	1.53 (1.13–2.03)	1.81 (1.67 to 1.95)
Tokelau	0.00 (0.00–0.01)	1.12 (0.74–1.61)	0.01 (0.01–0.01)	2.15 (1.57–2.90)	2.13 (2.11 to 2.15)
Tonga	0.27 (0.18–0.37)	1.78 (1.23–2.46)	0.58 (0.44–0.76)	2.67 (2.03–3.46)	1.17 (1.12 to 1.22)
Trinidad and Tobago	6.56 (4.77–8.75)	2.90 (2.12–3.86)	15.60 (11.31–20.81)	2.95 (2.13–3.95)	0.07 (-0.02 to 0.16)
Tunisia	25.22 (16.15–37.11)	1.88 (1.23–2.72)	124.47 (87.36–171.62)	3.47 (2.47–4.73)	2.07 (1.99 to 2.15)
Turkey	213.67 (149.13–295.63)	2.31 (1.64–3.14)	1185.34 (882.36–1558.73)	4.68 (3.52–6.11)	2.34 (2.24 to 2.44)
Turkmenistan	9.07 (5.95–13.09)	1.75 (1.18–2.48)	31.59 (22.89–42.47)	2.93 (2.19–3.83)	1.86 (1.79 to 1.93)
Tuvalu	0.02 (0.01–0.03)	1.19 (0.80–1.68)	0.06 (0.04–0.08)	2.07 (1.51–2.78)	1.6 (1.52 to 1.68)
Uganda	23.51 (16.56–32.21)	1.45 (1.04–1.96)	85.88 (64.57–111.11)	2.34 (1.79–2.98)	1.68 (1.56 to 1.8)
Ukraine	2729.72 (2204.69–3358.11)	13.71 (11.04–16.91)	2351.10 (1962.77–2777.68)	10.76 (8.95–12.77)	-0.85 (-1.04 to -0.66)
United Arab Emirates	3.54 (2.14–5.38)	2.77 (1.84–3.93)	61.27 (38.03–91.93)	4.13 (2.94–5.61)	0.93 (0.71 to 1.15)
United Kingdom	3302.69 (2623.51–4128.53)	13.02 (10.16–16.55)	5416.09 (4513.73–6423.33)	14.91 (12.27–17.89)	0.65 (0.59 to 0.7)

United Republic of Tanzania	34.15 (23.56–46.88)	1.24 (0.87–1.67)	139.13 (102.95–182.90)	2.13 (1.60–2.75)	1.76<br>(1.72 to 1.8)
United States Virgin Islands	0.79 (0.55–1.08)	3.41 (2.44–4.61)	2.25 (1.73–2.89)	4.42 (3.32–5.76)	0.89<br>(0.81 to 0.98)
United States of America	17331.27 (14216.50–20984.15)	19.65 (15.89–24.11)	24793.88 (20870.92–29286.61)	15.40 (12.81–18.38)	-0.91<br>(-1.01 to -0.81)
Uruguay	144.00 (117.90–173.47)	13.10 (10.67–15.86)	316.22 (265.50–373.04)	20.01 (16.69–23.75)	1.28<br>(1.14 to 1.41)
Uzbekistan	50.53 (30.79–76.92)	1.61 (1.00–2.41)	157.56 (106.53–222.48)	2.30 (1.65–3.12)	1.45<br>(1.34 to 1.56)
Vanuatu	0.16 (0.10–0.24)	0.94 (0.60–1.37)	0.63 (0.45–0.87)	1.31 (0.96–1.77)	1.08<br>(1.05 to 1.11)
Venezuela (Bolivarian Republic of)	121.67 (93.58–156.29)	4.85 (3.78–6.15)	679.48 (544.36–836.99)	8.42 (6.77–10.34)	1.87<br>(1.67 to 2.07)
Viet Nam	154.06 (106.09–214.81)	1.50 (1.05–2.08)	608.01 (433.26–833.95)	2.46 (1.81–3.30)	1.48<br>(1.39 to 1.58)
Yemen	16.83 (10.47–24.87)	1.31 (0.84–1.89)	65.88 (44.37–93.42)	1.79 (1.25–2.46)	1.22<br>(1.04 to 1.4)
Zambia	10.10 (7.13–13.63)	1.45 (1.05–1.92)	38.04 (28.54–49.82)	2.21 (1.70–2.83)	1.32<br>(1.19 to 1.44)
Zimbabwe	13.71 (8.36–20.81)	1.23 (0.78–1.82)	21.44 (14.22–30.50)	1.17 (0.82–1.61)	-0.62<br>(-0.81 to -0.42)

Abbreviations: VID, vascular intestinal diseases; ASR, age-standardized rate (per 100,000 population); EAPC, estimated annual percentage change; UI, uncertainty interval; CI, confidence interval.

Table S3. Global and regional DALYs of middle-aged and elderly VID in 1990 and 2021, and EAPC of ASR from 1990 to 2021.

Table S3. Global and regional DALYs of middle-aged and elderly VID in 1990 and 2021, and EAPC of ASR from 1990 to 2021.					
**Location**	**1990**		**2021**		**EAPC, 1990-2021**
	**Number**	**ASR**	**Number**	**ASR**	
Afghanistan	1643.26 (654.89–3127.92)	89.57 (36.32–172.77)	1180.70 (496.93–2288.74)	48.05 (21.03–91.16)	-2.3 (-2.44 to -2.16)
Albania	155.07 (110.57–207.65)	31.60 (22.35–42.42)	261.74 (173.14–387.65)	22.15 (14.56–32.89)	-1.02 (-1.2 to -0.83)
Algeria	1222.26 (662.97–2102.91)	42.40 (22.43–71.50)	1986.42 (1145.98–3167.98)	23.07 (13.11–36.83)	-2.04 (-2.18 to -1.89)
American Samoa	0.27 (0.17–0.39)	4.30 (2.81–6.42)	1.64 (0.90–2.49)	12.00 (6.43–18.28)	5.31 (4.38 to 6.25)
Andorra	16.94 (9.57–26.93)	117.28 (66.21–185.97)	37.78 (22.83–57.50)	84.03 (50.82–128.12)	-0.74 (-0.93 to -0.55)
Angola	457.89 (209.07–776.50)	49.45 (23.25–83.11)	1317.19 (632.37–2230.69)	47.57 (23.42–79.61)	-0.13 (-0.22 to -0.04)
Antigua and Barbuda	6.82 (5.53–8.34)	46.42 (37.63–56.89)	8.01 (6.44–9.78)	28.35 (22.84–34.62)	-1.71 (-2.03 to -1.4)
Argentina	16131.49 (13706.50–18810.50)	186.74 (158.39–217.84)	17783.73 (14970.10–20745.50)	114.52 (96.46–133.61)	-1.37 (-1.5 to -1.24)
Armenia	1407.38 (1191.22–1689.09)	196.37 (166.26–236.52)	3618.44 (2959.48–4224.69)	305.53 (250.06–356.78)	1.35 (1.09 to 1.62)
Australia	6389.56 (5479.93–7329.40)	119.84 (102.50–137.52)	8424.75 (6891.35–9952.48)	62.77 (51.66–74.08)	-2.09 (-2.24 to -1.95)
Austria	5049.64 (4281.04–5810.35)	147.01 (124.41–169.42)	3431.11 (2817.64–4041.43)	64.27 (53.21–75.71)	-2.87 (-3.18 to -2.56)
Azerbaijan	322.44 (213.55–472.07)	23.77 (15.73–34.56)	577.39 (351.59–882.03)	21.51 (13.17–32.72)	-0.15 (-0.33 to 0.02)
Bahamas	56.01 (45.58–68.51)	136.89 (111.39–167.56)	95.63 (73.85–122.98)	87.37 (67.78–111.68)	-1.35 (-1.48 to -1.22)
Bahrain	32.59 (18.59–47.71)	92.16 (51.56–135.41)	58.28 (38.41–85.60)	33.99 (22.50–49.96)	-4.11 (-4.43 to -3.8)
Bangladesh	10003.92 (5550.94–16485.34)	83.94 (46.31–138.13)	15961.08 (9058.11–25512.09)	45.28 (25.70–72.26)	-2.13 (-2.31 to -1.96)
Barbados	44.62 (36.66–53.68)	55.91 (46.08–67.10)	59.51 (42.34–78.26)	41.61 (29.65–54.71)	-0.96 (-1.08 to -0.85)
Belarus	7043.74 (5714.48–8913.71)	200.02 (162.37–252.16)	9488.34 (7349.87–12089.92)	211.96 (163.98–270.94)	0.18 (-0.04 to 0.39)
Belgium	9877.71 (8379.66–11376.85)	227.14 (192.10–261.88)	11205.54 (9184.40–13138.61)	163.04 (135.21–191.04)	-1.28 (-1.47 to -1.1)
Belize	8.36 (6.55–11.12)	33.72 (26.40–44.84)	24.66 (19.59–30.27)	31.19 (24.79–38.23)	-0.44 (-0.9 to 0.02)
Benin	329.94 (187.82–548.07)	61.11 (34.79–101.34)	761.56 (426.79–1262.09)	54.53 (30.99–89.41)	-0.4 (-0.49 to -0.3)
Bermuda	18.29 (14.03–22.70)	113.38 (87.11–140.48)	15.81 (11.96–20.69)	40.03 (30.31–52.39)	-3.83 (-4.16 to -3.5)
Bhutan	47.63 (22.01–86.28)	81.96 (38.63–146.37)	98.77 (55.18–167.10)	63.75 (35.73–107.27)	-0.88 (-0.98 to -0.77)

Bolivia (Plurinational State of)	995.95 (489.32–1611.06)	126.77 (62.33–205.98)	2147.72 (1176.95–3491.64)	95.29 (52.30–154.61)	-0.98 (-1.03 to -0.93)
Bosnia and Herzegovina	1002.66 (679.32–1380.41)	98.67 (66.88–135.38)	1384.40 (898.49–1957.48)	78.42 (50.85–110.98)	-0.81 (-0.97 to -0.64)
Botswana	63.56 (29.00–116.35)	45.21 (20.45–82.39)	129.21 (74.57–205.26)	34.85 (20.25–55.08)	-0.91 (-1.14 to -0.68)
Brazil	47834.45 (44235.86–51230.58)	209.95 (192.58–225.33)	84838.91 (76256.00–92346.80)	123.80 (111.04–134.83)	-1.89 (-1.97 to -1.81)
Brunei Darussalam	18.79 (11.62–27.94)	76.87 (46.90–115.65)	51.81 (34.69–73.62)	62.23 (40.79–89.03)	-0.18 (-0.45 to 0.08)
Bulgaria	4118.27 (3528.97–4783.94)	127.52 (109.22–147.48)	4396.01 (3584.97–5293.69)	108.22 (87.98–130.57)	0.33 (-0.05 to 0.71)
Burkina Faso	470.52 (265.02–777.63)	40.49 (22.88–66.76)	1081.15 (548.39–1894.55)	43.10 (22.04–74.98)	0.13 (-0.01 to 0.28)
Burundi	68.70 (9.11–181.25)	11.77 (1.53–32.52)	325.48 (100.42–650.59)	28.02 (8.61–56.12)	2.2 (1.57 to 2.83)
Cabo Verde	18.33 (10.19–33.44)	29.30 (16.25–53.63)	50.26 (26.66–83.14)	44.27 (23.35–73.24)	0.72 (0.39 to 1.06)
Cambodia	187.68 (96.02–372.78)	19.27 (9.63–39.22)	340.21 (171.45–742.49)	12.85 (6.30–29.05)	-1.62 (-1.81 to -1.44)
Cameroon	643.35 (339.63–1109.15)	55.07 (29.20–94.41)	1851.16 (974.60–3231.25)	54.93 (29.17–95.27)	-0.17 (-0.33 to 0)
Canada	13595.96 (11613.62–15538.59)	153.81 (131.05–176.05)	20348.42 (16761.63–23831.89)	97.94 (80.99–114.74)	-1.38 (-1.5 to -1.25)
Central African Republic	184.78 (101.60–302.90)	66.09 (37.17–107.06)	382.66 (199.17–639.98)	70.51 (37.84–115.96)	0.29 (0.2 to 0.37)
Chad	359.23 (189.36–638.22)	46.43 (24.44–82.61)	980.44 (523.86–1744.41)	62.94 (33.94–110.86)	0.92 (0.74 to 1.09)
Chile	5506.51 (4724.99–6361.24)	212.98 (182.47–246.00)	8355.95 (6976.01–9829.53)	117.05 (97.80–137.69)	-1.89 (-2.02 to -1.77)
China	12295.66 (9261.42–14920.52)	6.65 (4.78–8.13)	15970.74 (12474.11–19927.64)	2.96 (2.29–3.70)	-3.1 (-3.53 to -2.66)
Colombia	7450.39 (6385.27–8593.99)	168.18 (143.95–193.89)	16513.20 (12981.06–20417.26)	109.73 (86.35–135.63)	-1.33 (-1.63 to -1.03)
Comoros	22.06 (10.40–42.92)	44.42 (21.19–85.43)	46.13 (22.85–84.32)	36.96 (18.31–67.53)	-0.76 (-0.85 to -0.68)
Congo	174.82 (97.17–282.48)	67.34 (37.27–108.73)	430.59 (216.80–708.94)	66.37 (33.75–109.50)	-0.07 (-0.11 to -0.03)
Cook Islands	0.46 (0.29–0.70)	12.17 (7.81–18.56)	0.84 (0.51–1.34)	11.94 (7.25–19.17)	0.17 (0.11 to 0.24)
Costa Rica	1105.28 (930.13–1299.25)	244.15 (205.20–287.09)	2803.81 (2266.12–3352.93)	186.96 (151.45–223.47)	-0.95 (-1.16 to -0.74)
Coted'Ivoire	740.29 (397.63–1294.19)	70.51 (37.91–122.20)	1966.81 (1004.40–3609.15)	65.14 (33.67–118.27)	-0.51 (-0.73 to -0.3)
Croatia	3718.61 (3205.98–4246.42)	240.10 (207.70–273.07)	3750.68 (3160.06–4361.56)	142.51 (119.65–166.28)	-2 (-2.19 to -1.82)
Cuba	4107.36 (3467.68–4820.23)	149.66 (126.30–175.46)	7029.34 (5679.06–8551.74)	127.17 (102.74–154.86)	-0.66 (-0.78 to -0.55)
Cyprus	324.85 (186.08–515.44)	193.14 (108.03–312.72)	374.95 (251.04–541.22)	72.44 (47.57–106.12)	-3.34 (-3.5 to -3.18)

Czechia	5857.92 (5042.93–6712.67)	153.37 (131.86–175.80)	4942.54 (4074.75–5917.16)	79.53 (65.46–95.41)	-2.25 (-2.59 to -1.92)
Democratic People's Republic of Korea	193.55 (111.08–333.27)	5.10 (2.86–9.08)	332.83 (197.69–653.47)	3.84 (2.26–7.66)	-0.95 (-1.02 to -0.89)
Democratic Republic of the Congo	2281.20 (1299.21–3913.15)	61.67 (35.85–103.78)	5089.04 (2660.21–8803.06)	57.63 (30.44–99.01)	-0.23 (-0.3 to -0.15)
Denmark	2987.21 (2517.84–3495.46)	131.31 (110.67–153.77)	2370.67 (1951.54–2783.34)	67.74 (55.94–79.58)	-2.84 (-3.33 to -2.35)
Djibouti	16.90 (9.37–29.44)	52.24 (29.28–89.09)	76.09 (41.31–128.16)	49.39 (27.04–82.34)	-0.38 (-0.49 to -0.26)
Dominica	20.60 (13.31–29.72)	128.32 (82.64–185.47)	24.15 (15.33–35.82)	107.84 (68.37–160.28)	-0.61 (-0.65 to -0.57)
Dominican Republic	470.31 (304.17–672.10)	51.10 (32.95–72.95)	1076.92 (596.50–1714.59)	39.95 (22.15–63.57)	-0.43 (-0.57 to -0.29)
Ecuador	1501.83 (1259.04–1767.16)	114.42 (95.90–134.71)	3648.74 (2768.07–4695.28)	84.89 (64.69–108.75)	-0.39 (-0.73 to -0.04)
Egypt	3456.22 (2028.85–5171.57)	56.54 (32.30–85.60)	6734.83 (3934.05–10475.79)	46.14 (27.01–71.87)	-1.48 (-1.9 to -1.07)
El Salvador	749.85 (535.87–1012.37)	95.99 (68.54–129.55)	1329.56 (878.49–1897.61)	76.94 (50.95–109.71)	-0.67 (-0.84 to -0.5)
Equatorial Guinea	40.57 (21.21–75.74)	81.55 (43.89–147.08)	73.56 (35.01–128.25)	61.22 (28.65–107.33)	-1.09 (-1.29 to -0.89)
Eritrea	180.35 (98.14–299.36)	63.47 (33.56–109.04)	358.69 (188.85–582.36)	53.90 (28.43–88.27)	-0.67 (-0.75 to -0.59)
Estonia	1211.53 (1015.72–1457.46)	211.90 (177.68–254.90)	1972.48 (1615.92–2351.79)	256.07 (209.47–307.26)	0.61 (-0.05 to 1.27)
Eswatini	44.96 (25.68–75.07)	60.76 (34.51–101.70)	91.88 (52.11–150.69)	62.94 (35.84–102.26)	0.33 (-0.14 to 0.8)
Ethiopia	2500.01 (1633.25–3515.33)	51.02 (33.05–72.29)	3211.83 (1619.81–5047.47)	30.25 (15.38–47.48)	-2.18 (-2.36 to -2)
Fiji	11.64 (7.12–19.11)	10.98 (6.72–18.05)	26.26 (16.28–39.38)	11.98 (7.37–18.00)	0.65 (0.31 to 0.99)
Finland	3218.93 (2652.62–3826.28)	161.59 (132.73–192.15)	3263.80 (2647.45–3844.29)	84.62 (69.26–99.58)	-2.1 (-2.24 to -1.95)
France	43959.04 (37713.49–50349.34)	183.60 (157.28–210.58)	48667.64 (39096.68–58010.06)	114.41 (93.02–136.29)	-1.53 (-1.59 to -1.46)
Gabon	76.74 (40.03–128.40)	53.54 (27.52–90.08)	155.64 (77.38–270.98)	61.86 (30.08–109.06)	0.53 (0.42 to 0.64)
Gambia	73.98 (38.87–129.01)	77.80 (41.28–134.35)	218.32 (99.52–439.00)	82.94 (37.92–165.91)	0.04 (-0.1 to 0.18)
Georgia	42.20 (33.69–53.03)	2.49 (1.99–3.13)	2372.17 (1786.37–3058.25)	144.48 (108.43–187.03)	17.63 (15.55 to 19.74)
Germany	80566.97 (67142.59–94909.96)	219.62 (182.92–258.99)	61212.99 (50273.53–72023.18)	109.41 (90.93–128.18)	-2.27 (-2.54 to -2)
Ghana	820.43 (458.28–1410.86)	50.07 (28.27–85.99)	2538.98 (1366.26–4281.40)	58.31 (31.41–98.03)	0.59 (0.5 to 0.67)
Greece	2557.14 (2165.40–2977.28)	61.49 (51.94–71.54)	5515.58 (4486.51–6586.03)	71.87 (59.03–85.65)	0.15 (-0.18 to 0.47)
Greenland	31.35 (17.51–45.02)	376.38 (198.31–551.08)	48.70 (27.88–69.28)	256.81 (142.53–368.90)	-0.92 (-1.04 to -0.8)

Grenada	16.35 (12.73–20.89)	82.28 (63.63–105.04)	17.78 (14.27–21.95)	58.04 (46.55–71.56)	-1.03 (-1.17 to -0.89)
Guam	1.07 (0.68–1.54)	6.34 (3.84–9.33)	3.08 (2.15–4.26)	5.42 (3.77–7.54)	1.79 (0.55 to 3.04)
Guatemala	597.30 (485.74–716.53)	72.90 (59.41–87.54)	1167.91 (931.90–1436.37)	41.14 (32.86–50.45)	-1.8 (-1.97 to -1.63)
Guinea	435.24 (239.12–735.66)	49.48 (27.21–83.60)	856.55 (466.05–1449.95)	57.43 (31.28–96.63)	0.64 (0.5 to 0.79)
Guinea-Bissau	110.59 (51.30–211.32)	102.66 (48.27–194.65)	176.52 (82.99–334.25)	88.85 (42.86–166.44)	-0.25 (-0.39 to -0.12)
Guyana	86.12 (66.27–109.74)	86.77 (66.72–110.51)	120.14 (86.86–162.57)	68.56 (49.70–92.35)	-0.19 (-0.44 to 0.05)
Haiti	918.80 (503.89–1510.23)	112.02 (60.74–187.55)	1492.13 (730.08–2585.38)	82.16 (39.89–143.23)	-0.97 (-1.03 to -0.91)
Honduras	903.11 (603.25–1313.26)	173.68 (115.61–254.20)	2945.35 (1610.51–4764.35)	184.47 (101.27–297.89)	0.3 (0.16 to 0.45)
Hungary	12167.14 (10697.55–13772.15)	305.13 (267.29–346.10)	10365.07 (8666.54–12229.37)	188.30 (157.17–222.51)	-1.83 (-2.04 to -1.63)
Iceland	95.40 (79.10–112.43)	119.94 (99.53–141.36)	119.56 (94.07–144.76)	70.98 (56.30–85.85)	-1.76 (-2.03 to -1.49)
India	71977.45 (43532.60–113417.23)	62.96 (37.41–99.83)	128800.90 (91237.23–181421.02)	42.49 (30.09–59.88)	-1.35 (-1.45 to -1.24)
Indonesia	3696.24 (1945.56–6356.08)	18.36 (9.11–32.25)	6440.46 (3984.34–13001.61)	14.53 (8.60–29.99)	-0.9 (-0.98 to -0.83)
Iran (Islamic Republic of)	2564.62 (1970.06–3394.60)	41.40 (31.33–54.84)	4856.20 (3989.45–5990.75)	24.92 (20.39–30.86)	-1.56 (-1.68 to -1.43)
Iraq	402.72 (230.89–671.28)	19.20 (10.98–32.04)	952.22 (579.55–1462.66)	15.51 (9.46–23.59)	-0.97 (-1.08 to -0.87)
Ireland	1493.36 (1282.40–1728.70)	133.50 (114.30–154.75)	1863.04 (1522.86–2218.05)	82.41 (67.47–98.06)	-1.3 (-1.5 to -1.11)
Israel	1901.30 (1606.85–2227.54)	145.36 (122.49–170.07)	3385.07 (2707.78–3995.05)	93.59 (75.23–110.44)	-1.56 (-1.72 to -1.4)
Italy	31989.47 (28907.32–34273.78)	128.83 (115.84–138.33)	32203.57 (27005.54–35713.00)	69.49 (59.72–76.45)	-1.97 (-2.16 to -1.77)
Jamaica	172.62 (143.17–208.37)	35.72 (29.57–43.21)	251.80 (183.77–334.34)	29.50 (21.56–39.16)	-0.69 (-0.98 to -0.41)
Japan	22905.05 (20928.33–24600.33)	49.81 (45.24–53.59)	61818.50 (50109.35–69506.23)	51.50 (43.70–57.22)	0.31 (0.06 to 0.56)
Jordan	213.71 (132.28–325.66)	67.46 (41.39–103.11)	862.24 (572.69–1256.69)	47.39 (31.49–68.52)	-1.15 (-1.41 to -0.88)
Kazakhstan	4749.56 (4010.90–5712.69)	143.15 (120.59–173.26)	5765.66 (4438.38–7532.03)	120.18 (92.88–155.68)	-1.3 (-1.76 to -0.85)
Kenya	848.22 (395.31–1589.97)	42.51 (19.80–80.30)	2694.90 (1343.92–4493.27)	49.46 (24.47–83.80)	0.82 (0.64 to 1)
Kiribati	8.36 (4.76–13.51)	82.32 (46.36–134.39)	16.19 (9.21–26.47)	77.91 (44.12–127.28)	-0.07 (-0.16 to 0.02)
Kuwait	147.79 (114.07–183.64)	102.75 (78.54–128.28)	460.99 (346.10–595.67)	65.70 (49.46–84.40)	-0.85 (-2.03 to 0.33)
Kyrgyzstan	126.27 (103.65–156.63)	15.84 (13.02–19.58)	592.13 (450.18–757.76)	46.59 (35.40–59.49)	4.02 (3.44 to 4.61)

Lao People's Democratic Republic	62.28 (31.97–114.90)	14.16 (6.70–27.31)	91.67 (49.78–171.17)	9.13 (4.83–17.42)	-1.58 (-1.68 to -1.49)
Latvia	2372.05 (2022.84–2790.21)	237.99 (202.72–279.88)	3581.98 (2947.98–4261.64)	317.80 (259.97–381.04)	0.67 (0.12 to 1.23)
Lebanon	606.08 (326.47–1010.76)	113.08 (60.33–189.92)	1046.38 (669.38–1538.61)	60.95 (39.09–89.67)	-1.85 (-1.94 to -1.76)
Lesotho	79.85 (39.28–158.91)	36.16 (17.50–72.68)	139.82 (79.15–226.48)	49.66 (27.83–80.81)	1.43 (1 to 1.85)
Liberia	188.00 (104.81–312.61)	60.28 (33.62–100.06)	349.27 (177.58–635.40)	60.64 (31.67–108.50)	-0.13 (-0.26 to 0)
Libya	254.47 (116.00–569.56)	53.46 (24.11–120.27)	464.54 (253.66–789.63)	35.18 (19.09–60.15)	-1.08 (-1.36 to -0.81)
Lithuania	1715.24 (1413.86–2090.25)	138.62 (114.18–168.93)	4899.91 (4009.75–5797.27)	291.16 (237.60–345.65)	2.86 (2.23 to 3.5)
Luxembourg	334.76 (286.20–388.64)	223.33 (190.54–259.33)	400.96 (328.65–479.64)	129.21 (106.30–154.60)	-1.91 (-2.09 to -1.73)
Madagascar	674.37 (369.27–1216.27)	53.24 (29.03–95.96)	1317.60 (682.55–2510.43)	48.53 (25.24–91.91)	-0.26 (-0.29 to -0.22)
Malawi	465.50 (234.09–811.27)	49.00 (24.64–85.40)	872.78 (423.16–1482.90)	47.04 (22.91–79.94)	-0.4 (-0.63 to -0.16)
Malaysia	1317.27 (872.42–1912.66)	57.89 (38.20–84.23)	5601.32 (3628.74–8112.51)	82.46 (53.05–119.72)	1.09 (0.79 to 1.4)
Maldives	1.77 (0.76–3.58)	9.28 (3.97–18.76)	4.31 (2.51–6.87)	5.93 (3.42–9.51)	-1.8 (-1.95 to -1.65)
Mali	740.04 (377.53–1362.49)	70.32 (36.31–128.56)	1545.68 (844.02–2697.86)	65.81 (36.51–113.84)	0.06 (-0.08 to 0.21)
Malta	136.23 (115.31–158.64)	119.23 (100.54–138.92)	210.87 (171.40–251.53)	73.72 (60.09–88.15)	-1.89 (-2.18 to -1.59)
Marshall Islands	0.47 (0.27–0.80)	11.12 (6.26–19.20)	1.11 (0.61–1.84)	10.87 (6.05–18.05)	0.07 (-0.11 to 0.25)
Mauritania	158.80 (76.29–268.91)	57.00 (27.65–96.37)	284.15 (150.26–481.25)	49.81 (26.55–83.74)	-0.61 (-0.72 to -0.49)
Mauritius	49.19 (40.94–58.15)	29.83 (24.70–35.26)	83.59 (67.85–100.15)	17.48 (14.20–20.93)	0.28 (-0.52 to 1.09)
Mexico	20137.49 (18907.25–21372.24)	194.11 (181.48–206.36)	46499.91 (40175.40–52776.46)	139.31 (120.55–157.83)	-0.98 (-1.11 to -0.85)
Micronesia (Federated States of)	1.55 (0.90–2.55)	12.07 (6.94–20.02)	2.71 (1.58–4.40)	12.52 (7.27–20.35)	0.16 (0.06 to 0.25)
Monaco	28.80 (18.15–42.49)	137.28 (86.29–203.93)	36.27 (23.46–52.79)	121.07 (78.25–177.05)	-0.35 (-0.46 to -0.25)
Mongolia	64.84 (42.38–96.61)	23.75 (15.48–35.51)	202.23 (135.78–280.76)	34.22 (22.49–47.60)	1.01 (0.83 to 1.19)
Montenegro	451.20 (331.35–611.78)	268.35 (197.10–363.37)	758.35 (543.96–1017.62)	288.37 (206.44–386.64)	0.29 (0.19 to 0.39)
Morocco	1656.16 (885.96–2859.78)	45.18 (23.94–78.45)	2608.30 (1507.32–4169.93)	29.46 (17.06–47.09)	-1.45 (-1.56 to -1.34)
Mozambique	889.51 (493.32–1471.48)	58.60 (32.68–96.29)	1993.24 (1075.76–3296.04)	68.77 (36.94–113.25)	0.95 (0.8 to 1.1)
Myanmar	608.55 (279.79–1218.76)	10.53 (4.65–21.64)	672.25 (301.80–1386.53)	5.33 (2.36–11.28)	-2.79 (-3.02 to -2.56)

Namibia	89.88 (42.90–174.77)	54.92 (25.44–108.39)	184.64 (103.02–302.04)	51.86 (29.04–84.83)	-0.41 (-0.7 to -0.11)
Nauru	0.16 (0.09–0.26)	12.45 (7.03–19.72)	0.23 (0.11–0.39)	13.91 (6.71–23.52)	0.41 (0.16 to 0.67)
Nepal	1724.48 (745.18–3597.30)	75.78 (31.81–159.96)	3448.97 (1913.26–5558.44)	60.08 (33.25–97.37)	-0.86 (-1.05 to -0.66)
Netherlands	7806.88 (6632.37–8975.09)	139.52 (118.39–160.49)	9332.59 (7713.69–10862.51)	90.40 (75.04–105.19)	-1.58 (-1.82 to -1.35)
New Zealand	1349.72 (1154.59–1540.79)	124.62 (106.33–142.44)	1829.59 (1512.77–2112.32)	74.47 (61.80–85.86)	-1.74 (-2.36 to -1.11)
Nicaragua	186.79 (132.84–249.28)	49.05 (34.62–65.81)	527.60 (355.25–740.53)	42.17 (28.32–59.23)	-0.17 (-0.43 to 0.1)
Niger	324.60 (143.50–659.50)	44.97 (20.20–90.68)	745.28 (238.69–1802.57)	33.38 (10.79–80.22)	-1.14 (-1.35 to -0.92)
Nigeria	5788.46 (3764.70–8739.03)	48.57 (31.58–72.59)	11104.64 (7081.05–16131.53)	46.26 (30.17–65.46)	-0.16 (-0.33 to 0.02)
Niue	0.05 (0.03–0.08)	8.57 (5.20–13.39)	0.06 (0.04–0.09)	9.66 (5.99–15.23)	0.38 (0.27 to 0.5)
North Macedonia	602.70 (445.46–794.03)	124.11 (91.97–162.89)	895.56 (606.40–1229.50)	108.14 (73.17–147.98)	-0.58 (-0.86 to -0.3)
Northern Mariana Islands	0.45 (0.27–0.68)	10.41 (6.29–15.67)	2.58 (1.41–3.75)	18.49 (9.40–27.38)	3.04 (2.18 to 3.91)
Norway	2394.12 (2162.89–2579.99)	119.92 (108.67–129.18)	1692.18 (1459.51–1869.88)	56.34 (49.00–62.14)	-2.19 (-2.44 to -1.95)
Oman	29.95 (16.74–50.24)	17.55 (9.83–29.13)	85.93 (49.89–140.27)	18.11 (10.59–28.77)	1.02 (0.6 to 1.45)
Pakistan	9700.87 (3580.42–19808.07)	69.43 (25.51–142.60)	19196.21 (10318.13–29702.76)	69.00 (37.26–106.73)	-0.44 (-0.58 to -0.3)
Palau	0.25 (0.15–0.41)	9.89 (5.83–15.84)	0.65 (0.38–1.02)	10.10 (5.94–16.11)	0.18 (0.06 to 0.31)
Palestine	147.19 (78.11–262.83)	67.97 (36.08–122.11)	207.14 (135.94–302.39)	34.56 (22.59–50.29)	-2.42 (-2.52 to -2.33)
Panama	400.21 (334.50–477.01)	103.80 (86.61–123.83)	867.76 (648.75–1091.72)	71.18 (53.27–89.54)	-1.03 (-1.22 to -0.85)
Papua New Guinea	21.68 (11.06–37.59)	4.17 (2.16–7.24)	60.46 (35.32–99.01)	3.99 (2.33–6.53)	-0.15 (-0.19 to -0.12)
Paraguay	735.68 (517.89–994.64)	126.51 (88.85–170.88)	2148.03 (1442.40–3125.21)	139.53 (93.88–202.61)	0.52 (0.4 to 0.65)
Peru	1916.03 (1266.74–2718.10)	62.07 (41.03–87.91)	3737.09 (2364.18–5559.72)	41.30 (26.16–61.39)	-1.81 (-2.16 to -1.46)
Philippines	428.47 (257.35–601.86)	6.62 (3.65–9.55)	1419.28 (1055.25–1719.71)	7.24 (5.16–8.79)	0.44 (0.32 to 0.55)
Poland	28710.36 (26903.98–30351.66)	247.17 (230.59–261.94)	29930.76 (26211.21–33225.53)	146.03 (128.20–162.10)	-1.92 (-2.25 to -1.58)
Portugal	6358.87 (5442.63–7380.63)	171.76 (146.50–199.21)	12525.47 (10057.00–14724.42)	161.29 (130.76–189.72)	-0.12 (-0.33 to 0.09)
Puerto Rico	928.25 (776.34–1092.38)	96.24 (80.41–113.42)	573.95 (444.36–717.55)	28.38 (22.04–35.59)	-3.9 (-4.17 to -3.63)
Qatar	10.07 (6.01–15.73)	47.10 (27.89–72.28)	41.84 (25.96–63.87)	21.81 (13.29–33.11)	-2.62 (-3.13 to -2.11)

Republic of Korea	3653.45 (2619.13–4974.10)	52.06 (37.02–71.91)	9757.54 (6874.73–13162.37)	38.07 (26.69–51.45)	-0.49 (-0.69 to -0.3)
Republic of Moldova	1334.30 (1101.49–1635.91)	115.50 (95.65–140.66)	4276.46 (3490.95–5146.42)	257.57 (210.22–310.13)	3.21 (2.86 to 3.55)
Romania	11644.24 (9462.85–14455.50)	155.24 (126.56–191.46)	21226.43 (17100.99–26080.80)	203.68 (163.64–250.54)	0.95 (0.76 to 1.14)
Russian Federation	97388.81 (88413.68–118264.71)	199.69 (180.84–241.95)	230811.74 (208388.70–251407.96)	348.06 (313.99–379.22)	1.89 (1.67 to 2.11)
Rwanda	196.54 (56.63–369.22)	28.23 (7.91–55.00)	296.56 (63.90–825.55)	19.50 (4.21–55.01)	-2.5 (-2.97 to -2.03)
Saint Kitts and Nevis	11.54 (8.90–14.34)	114.68 (88.15–142.32)	12.69 (9.84–15.75)	71.73 (56.08–88.45)	-1.27 (-1.45 to -1.09)
Saint Lucia	23.42 (18.74–28.49)	107.04 (85.73–130.45)	40.66 (30.29–51.81)	62.24 (46.30–79.27)	-2.2 (-2.43 to -1.97)
Saint Vincent and the Grenadines	5.88 (4.86–7.08)	30.91 (25.52–37.24)	7.89 (6.34–9.74)	20.32 (16.34–25.02)	-1.37 (-1.68 to -1.05)
Samoa	1.65 (0.93–2.70)	7.26 (4.05–11.92)	3.26 (1.96–5.13)	8.08 (4.84–12.74)	0.4 (0.36 to 0.43)
San Marino	3.81 (2.47–5.51)	37.15 (24.03–53.72)	4.69 (2.76–7.25)	18.13 (10.70–28.08)	-1.45 (-1.77 to -1.13)
Sao Tome and Principe	8.45 (4.99–13.55)	50.06 (29.36–80.33)	16.91 (7.36–31.07)	58.55 (25.49–107.19)	0.49 (0.37 to 0.6)
Saudi Arabia	946.13 (435.82–1816.35)	66.96 (30.70–129.73)	1773.02 (1021.99–2851.89)	37.01 (22.03–58.45)	-2.11 (-2.21 to -2.01)
Senegal	709.18 (393.90–1171.29)	81.96 (45.66–135.10)	1356.63 (685.53–2327.89)	65.20 (32.79–111.67)	-0.86 (-0.96 to -0.76)
Serbia	7060.38 (5087.14–9534.02)	260.11 (186.83–352.81)	8670.69 (6176.96–11709.82)	183.36 (130.37–248.32)	-1.29 (-1.45 to -1.13)
Seychelles	1.58 (0.98–2.80)	10.46 (6.52–18.64)	2.35 (1.45–5.11)	8.54 (5.21–19.40)	-0.83 (-1.07 to -0.6)
Sierra Leone	311.36 (175.62–521.95)	55.67 (31.46–93.09)	571.12 (321.09–959.57)	55.29 (31.49–91.98)	0.1 (0.05 to 0.15)
Singapore	345.71 (291.10–407.60)	61.28 (51.53–72.21)	999.77 (812.12–1187.89)	43.58 (35.37–51.72)	-0.52 (-0.79 to -0.25)
Slovakia	1858.73 (1342.36–2501.33)	114.31 (82.30–153.94)	2378.14 (1636.89–3294.83)	89.59 (61.48–124.30)	-0.58 (-0.72 to -0.43)
Slovenia	1645.31 (1413.78–1894.95)	241.74 (207.17–278.99)	1798.18 (1447.00–2136.13)	131.18 (105.87–156.03)	-2.28 (-2.65 to -1.91)
Solomon Islands	1.66 (0.75–2.98)	4.32 (2.04–7.68)	5.04 (2.70–8.39)	4.98 (2.69–8.20)	0.6 (0.5 to 0.7)
Somalia	356.20 (189.51–590.00)	61.30 (33.23–100.72)	786.04 (403.39–1336.67)	53.63 (28.13–90.80)	-0.52 (-0.6 to -0.44)
South Africa	2158.78 (1420.87–2905.50)	40.25 (25.94–54.43)	6326.87 (4566.85–7670.41)	52.09 (37.07–63.39)	1.03 (0.74 to 1.31)
South Sudan	354.81 (169.36–645.22)	53.94 (25.76–98.06)	515.26 (231.69–929.72)	53.06 (24.19–94.71)	-0.3 (-0.42 to -0.17)
Spain	36035.86 (30619.00–41429.50)	238.64 (202.15–274.50)	44484.22 (35449.53–52332.28)	142.82 (115.59–167.68)	-1.75 (-1.98 to -1.52)
Sri Lanka	443.26 (292.32–656.58)	18.61 (12.39–27.34)	703.77 (417.18–1083.39)	10.46 (6.22–16.13)	-1.57 (-1.71 to -1.43)

Sudan	1134.51 (485.07–2337.52)	47.88 (20.18–99.75)	1612.32 (848.97–3154.15)	33.24 (17.52–65.02)	-1.26 (-1.41 to -1.11)
Suriname	30.51 (20.48–42.75)	45.19 (30.33–63.29)	61.25 (37.67–92.77)	35.32 (21.70–53.48)	-0.79 (-0.97 to -0.62)
Sweden	5890.70 (5085.30–6699.13)	131.15 (113.21–149.17)	3421.82 (2746.21–4076.15)	50.57 (40.87–60.33)	-3.03 (-3.19 to -2.86)
Switzerland	2340.90 (1946.23–2750.18)	78.39 (65.24–92.10)	3184.90 (2522.24–3812.57)	57.14 (45.76–68.34)	-1.05 (-1.4 to -0.7)
Syrian Arab Republic	630.06 (362.34–997.34)	49.26 (28.10–78.45)	1017.42 (613.00–1559.11)	30.96 (18.89–46.92)	-1.97 (-2.24 to -1.7)
Taiwan (Province of China)	675.54 (567.92–795.15)	17.99 (15.06–21.21)	8110.91 (6493.02–9667.81)	68.44 (54.97–81.51)	2.87 (1.38 to 4.38)
Tajikistan	195.05 (131.83–303.61)	27.23 (18.40–42.71)	525.68 (298.14–852.02)	36.73 (21.01–58.09)	1.13 (0.73 to 1.54)
Thailand	4002.81 (2525.47–5981.16)	48.82 (30.64–73.47)	9583.50 (6073.16–14410.24)	32.15 (20.39–48.33)	-1.54 (-1.72 to -1.35)
Timor-Leste	5.55 (2.16–12.36)	9.36 (3.45–20.87)	14.31 (5.55–29.95)	7.07 (2.66–14.83)	-1.03 (-1.27 to -0.8)
Togo	227.94 (123.70–387.54)	69.18 (37.94–116.38)	746.85 (410.43–1277.80)	68.69 (38.40–115.42)	-0.17 (-0.32 to -0.01)
Tokelau	0.03 (0.02–0.06)	9.38 (5.30–15.63)	0.03 (0.02–0.05)	8.40 (5.22–12.99)	-0.33 (-0.46 to -0.19)
Tonga	1.72 (1.03–2.78)	11.47 (6.85–18.78)	2.93 (1.77–4.73)	13.18 (7.99–21.25)	0.6 (0.41 to 0.79)
Trinidad and Tobago	205.23 (171.49–243.45)	94.37 (78.27–112.30)	285.62 (202.29–384.66)	53.71 (38.09–72.22)	-2.14 (-2.32 to -1.96)
Tunisia	442.21 (263.36–693.30)	35.98 (21.19–56.91)	760.34 (433.62–1253.93)	22.08 (12.60–36.40)	-1.8 (-1.93 to -1.68)
Turkey	7502.62 (4651.83–11744.76)	84.43 (52.35–132.69)	11282.88 (7458.14–16317.96)	45.62 (30.17–65.99)	-1.98 (-2.13 to -1.83)
Turkmenistan	250.70 (201.48–314.46)	51.23 (41.12–64.72)	576.29 (411.46–785.98)	54.83 (39.54–74.16)	0.01 (-0.16 to 0.18)
Tuvalu	0.20 (0.12–0.33)	11.12 (6.29–18.51)	0.31 (0.19–0.48)	10.69 (6.46–16.72)	-0.02 (-0.11 to 0.06)
Uganda	438.64 (177.63–770.33)	27.53 (11.16–48.60)	949.72 (385.26–1680.88)	26.69 (11.00–47.07)	-0.57 (-0.77 to -0.37)
Ukraine	47824.70 (40471.88–56012.62)	242.77 (205.33–284.53)	47979.07 (35073.36–63186.38)	220.05 (160.38–290.55)	-0.46 (-0.63 to -0.3)
United Arab Emirates	96.99 (52.77–159.55)	104.01 (56.68–172.98)	378.66 (221.67–599.23)	67.20 (38.44–109.03)	0.49 (-0.1 to 1.08)
United Kingdom	33854.03 (31713.65–35428.00)	133.62 (125.21–139.83)	56371.79 (50327.92–60602.66)	150.75 (135.81–161.82)	0.63 (0.45 to 0.81)
United Republic of Tanzania	988.67 (470.06–1659.08)	36.30 (17.23–61.08)	2621.55 (1094.24–4903.66)	40.88 (17.29–76.27)	0.42 (0.24 to 0.59)
United States Virgin Islands	17.21 (10.85–25.35)	82.23 (51.10–121.58)	17.23 (10.20–26.80)	34.87 (20.55–54.49)	-2.66 (-2.81 to -2.5)
United States of America	147722.71 (135031.12–156645.62)	166.30 (152.40–176.23)	181772.10 (163195.46–193409.12)	112.51 (101.68–119.49)	-1.58 (-1.78 to -1.37)
Uruguay	4147.78 (3558.91–4781.74)	384.29 (329.01–443.40)	4652.23 (3918.77–5430.07)	290.10 (245.33–338.55)	-0.94 (-1.16 to -0.73)

Uzbekistan	397.24 (306.98–519.69)	13.17 (10.17–17.31)	1589.77 (1232.42–2001.90)	23.88 (18.70–29.80)	2.08 (1.5 to 2.65)
Vanuatu	1.91 (0.87–4.00)	11.17 (5.01–23.43)	6.86 (3.60–12.75)	13.62 (7.06–25.46)	0.73 (0.61 to 0.85)
Venezuela (Bolivarian Republic of)	3808.08 (3202.03–4473.08)	155.56 (130.26–182.94)	11400.14 (7723.88–15515.10)	143.18 (97.82–193.94)	-0.28 (-0.41 to -0.16)
Viet Nam	836.64 (438.22–1533.96)	8.79 (4.51–16.29)	1555.00 (878.55–2938.11)	6.82 (3.80–13.21)	-0.9 (-0.98 to -0.82)
Yemen	572.62 (245.44–1195.86)	48.93 (20.49–105.50)	999.52 (548.18–1706.28)	29.33 (16.13–50.25)	-2.07 (-2.25 to -1.89)
Zambia	414.07 (247.11–643.69)	58.48 (35.10–90.71)	947.36 (527.93–1538.41)	54.31 (30.48–87.95)	-0.54 (-0.76 to -0.32)
Zimbabwe	381.76 (237.10–581.46)	37.82 (23.50–57.69)	907.62 (509.82–1484.99)	50.77 (28.88–82.32)	1.15 (0.68 to 1.63)

Abbreviations: VID, vascular intestinal diseases; DALYs, disability-adjusted life years; ASR, age-standardized rate (per 100,000 population); EAPC, estimated annual percentage change; UI, uncertainty interval; CI, confidence interval.

Table S4. Relative risks (RRs) of middle-aged and elderly VID incidence and prevalence due to age, period and birth cohort effects stratified by sexes.

**Table S4** RRs of middle-aged and elderly VID incidence and prevalence due to age, period and birth cohort effects stratified by sexes.								
**Factor**	**Incidence**				**Prevalence**			
	**Male**		**Female**		**Male**		**Female**	
	**RR (95% CI)**	***P***	**RR (95% CI)**	***P***	**RR (95% CI)**	***P***	**RR (95% CI)**	***P***
**Age (years)**								
45-49	0.273(0.270-0.276)	<0.001	0.261(0.259-0.263)	<0.001	0.272(0.263-0.281)	<0.001	0.261(0.255-0.267)	<0.001
50-54	0.371(0.368-0.374)	<0.001	0.353(0.351-0.355)	<0.001	0.377(0.367-0.387)	<0.001	0.347(0.341-0.353)	<0.001
55-59	0.492(0.489-0.495)	<0.001	0.454(0.452-0.456)	<0.001	0.519(0.508-0.530)	<0.001	0.450(0.443-0.457)	<0.001
60-64	0.683(0.680-0.687)	<0.001	0.623(0.620-0.625)	<0.001	0.716(0.704-0.728)	<0.001	0.617(0.610-0.625)	<0.001
65-69	0.999(0.994-1.003)	0.555	0.908(0.905-0.911)	<0.001	1.039(1.026-1.053)	<0.001	0.908(0.898-0.918)	<0.001
70-74	1.405(1.399-1.411)	<0.001	1.346(1.341-1.351)	<0.001	1.468(1.450-1.486)	<0.001	1.361(1.348-1.375)	<0.001
75-79	1.730(1.722-1.739)	<0.001	1.803(1.796-1.809)	<0.001	1.867(1.839-1.895)	<0.001	1.958(1.937-1.979)	<0.001
80-84	1.996(1.983-2.009)	<0.001	2.165(2.156-2.174)	<0.001	2.096(2.055-2.139)	<0.001	2.351(2.322-2.381)	<0.001
85-89	2.026(2.009-2.044)	<0.001	2.206(2.194-2.217)	<0.001	2.137(2.081-2.195)	<0.001	2.446(2.407-2.485)	<0.001
90-94	1.802(1.780-1.824)	<0.001	1.979(1.965-1.994)	<0.001	1.662(1.600-1.727)	<0.001	1.957(1.914-2.000)	<0.001
95-99	1.663(1.629-1.698)	<0.001	1.846(1.825-1.867)	<0.001	1.238(1.156-1.326)	<0.001	1.461(1.407-1.517)	<0.001
**Period**								
1992	0.868(0.863-0.873)	<0.001	0.860(0.857-0.863)	<0.001	0.871(0.855-0.886)	<0.001	0.848(0.838-0.857)	<0.001
1997	0.925(0.921-0.929)	<0.001	0.930(0.927-0.933)	<0.001	0.932(0.920-0.944)	<0.001	0.927(0.918-0.935)	<0.001
2002	0.982(0.979-0.986)	<0.001	0.998(0.996-1.001)	0.240	0.988(0.979-0.997)	0.009	0.998(0.990-1.006)	0.649
2007	1.040(1.037-1.043)	<0.001	1.049(1.046-1.052)	<0.001	1.044(1.036-1.053)	<0.001	1.054(1.046-1.062)	<0.001
2012	1.084(1.080-1.088)	<0.001	1.082(1.079-1.086)	<0.001	1.071(1.058-1.083)	<0.001	1.081(1.072-1.090)	<0.001
2017	1.126(1.120-1.132)	<0.001	1.102(1.099-1.106)	<0.001	1.116(1.097-1.135)	<0.001	1.120(1.108-1.132)	<0.001
**Birth cohort**								
1897	1.527(1.418-1.644)	<0.001	1.600(1.538-1.664)	<0.001	1.429(1.113-1.834)	0.005	1.547(1.356-1.765)	<0.001
1902	1.448(1.400-1.498)	<0.001	1.546(1.517-1.576)	<0.001	1.379(1.240-1.535)	<0.001	1.546(1.457-1.640)	<0.001

1907	1.422(1.392-1.453)	<0.001	1.492(1.474-1.511)	<0.001	1.428(1.336-1.528)	<0.001	1.547(1.490-1.607)	<0.001
1912	1.416(1.393-1.441)	<0.001	1.440(1.426-1.454)	<0.001	1.452(1.376-1.533)	<0.001	1.499(1.454-1.545)	<0.001
1917	1.325(1.306-1.344)	<0.001	1.343(1.332-1.354)	<0.001	1.337(1.277-1.401)	<0.001	1.360(1.325-1.396)	<0.001
1922	1.264(1.249-1.280)	<0.001	1.284(1.275-1.293)	<0.001	1.313(1.262-1.365)	<0.001	1.312(1.283-1.342)	<0.001
1927	1.195(1.183-1.207)	<0.001	1.184(1.176-1.191)	<0.001	1.235(1.195-1.276)	<0.001	1.199(1.176-1.222)	<0.001
1932	1.077(1.068-1.086)	<0.001	1.047(1.041-1.052)	<0.001	1.115(1.086-1.146)	<0.001	1.060(1.042-1.078)	<0.001
1937	1.010(1.003-1.017)	0.004	0.974(0.970-0.979)	<0.001	1.034(1.012-1.056)	0.002	0.976(0.962-0.990)	0.001
1942	0.915(0.910-0.921)	<0.001	0.911(0.907-0.915)	<0.001	0.926(0.911-0.941)	<0.001	0.909(0.897-0.921)	<0.001
1947	0.860(0.856-0.864)	<0.001	0.871(0.867-0.874)	<0.001	0.863(0.853-0.874)	<0.001	0.865(0.855-0.875)	<0.001
1952	0.795(0.792-0.799)	<0.001	0.788(0.784-0.791)	<0.001	0.796(0.786-0.805)	<0.001	0.780(0.770-0.790)	<0.001
1957	0.745(0.741-0.749)	<0.001	0.727(0.724-0.731)	<0.001	0.742(0.731-0.753)	<0.001	0.719(0.709-0.730)	<0.001
1962	0.678(0.673-0.682)	<0.001	0.653(0.649-0.657)	<0.001	0.669(0.656-0.683)	<0.001	0.644(0.632-0.655)	<0.001
1967	0.592(0.586-0.597)	<0.001	0.569(0.564-0.573)	<0.001	0.579(0.563-0.596)	<0.001	0.556(0.543-0.569)	<0.001
1972	0.551(0.543-0.559)	<0.001	0.535(0.529-0.543)	<0.001	0.534(0.511-0.558)	<0.001	0.516(0.496-0.537)	<0.001

Abbreviations: VID, vascular intestinal diseases; RR, relative risk; UI, uncertainty interval.

Table S5. Changes in the number of DALYs of middle-aged and elderly VID according to population-level determinants from 1990 to 2021.

Table S5. Changes in the number of DALYs of middle-aged and elderly VID according to population-level determinants from 1990 to 2021.					
Sex	Location	Overall Difference	Aging	Population	Epidemiological Change
Both	Global	529349.42	125987.45 (23.8%)	1003828.12 (189.63%)	-600466.14 (-113.43%)
	High SDI	111001.46	74705.11 (67.3%)	284241.22 (256.07%)	-247944.87 (-223.37%)
	High-middle SDI	191121.22	43564.38 (22.79%)	282398.21 (147.76%)	-134841.36 (-70.55%)
	Middle SDI	123543.12	19844.27 (16.06%)	167286.91 (135.41%)	-63588.06 (-51.47%)
	Low-middle SDI	80107	8642.92 (10.79%)	112470.37 (140.4%)	-41006.29 (-51.19%)
	Low SDI	23282.92	-226.63 (-0.97%)	37692.55 (161.89%)	-14183 (-60.92%)
Male	Global	243371.76	61972.86 (25.46%)	469875.69 (193.07%)	-288476.78 (-118.53%)
	High SDI	66050.91	38300.14 (57.99%)	131936.62 (199.75%)	-104185.86 (-157.74%)
	High-middle SDI	71250.32	21681.14 (30.43%)	142661.9 (200.23%)	-93092.72 (-130.66%)
	Middle SDI	56780.7	9276.01 (16.34%)	81314.95 (143.21%)	-33810.26 (-59.55%)
	Low-middle SDI	34870.95	2754.96 (7.9%)	53023.59 (152.06%)	-20907.61 (-59.96%)
	Low SDI	14314.77	-475.4 (-3.32%)	21845.19 (152.61%)	-7055.02 (-49.28%)
Female	Global	285977.66	60576.42 (21.18%)	533675.89 (186.61%)	-308274.65 (-107.8%)
	High SDI	44950.55	37672.49 (83.81%)	150264.92 (334.29%)	-142986.85 (-318.1%)
	High-middle SDI	119870.91	22958.19 (19.15%)	140659.73 (117.34%)	-43747.02 (-36.5%)
	Middle SDI	66762.42	10264.02 (15.37%)	85820.53 (128.55%)	-29322.13 (-43.92%)
	Low-middle SDI	45236.05	6050.86 (13.38%)	59391.8 (131.29%)	-20206.6 (-44.67%)
	Low SDI	8968.14	197.64 (2.2%)	15556.56 (173.46%)	-6786.05 (-75.67%)

Abbreviations: VID, vascular intestinal diseases; DALYs, disability-adjusted life years.

Table S6 The predicted case number and ASR of incidence, DALYs, and prevalence of middle-aged and elderly VID from 2022 to 2040 globally.

Table S6 The predicted case number and ASR of incidence, DALYs, and prevalence of middle-aged and elderly VID from 2022 to 2040 globally.						
	**Incidence**		**Prevalence**		**DALYs**	
**Year**	**Case number**	**ASR**	**Case number**	**ASR**	**Case number**	**ASR**
2022	1171709.315	48.23678455	147272.9392	6.067186329	1572880.306	65.53571166
2023	1192816.739	47.74424674	150244.8837	6.016595245	1588845.222	64.31479694
2024	1215342.466	47.25737892	153412.6618	5.966746311	1607676.073	63.13058301
2025	1238605.244	46.77670663	156699.4819	5.917732534	1627439.852	61.98017714
2026	1261591.156	46.29280225	159977.6134	5.868144706	1645950.934	60.83643525
2027	1284122.026	45.82360891	163211.5782	5.819758133	1663282.8	59.73793095
2028	1307148.729	45.36229938	166505.1346	5.771747496	1683587.432	58.75075291
2029	1331713.633	44.91643848	169985.6737	5.725013054	1707064.81	57.83036223
2030	1357053.031	44.48687794	173572.9467	5.680025994	1731883.991	56.96351297
2031	1381759.41	44.05114149	177116.3752	5.634724553	1755551.364	56.10273042
2032	1405242.086	43.61395205	180553.7891	5.589984672	1777737.908	55.26537825
2033	1428674.699	43.18252848	183989.5666	5.545632781	1802942.873	54.53751237
2034	1453411.623	42.77574638	187572.1673	5.503445712	1831547.364	53.89368805
2035	1478745.934	42.3972722	191221.8329	5.46409099	1861608.954	53.31377924
2036	1503243.942	42.0272008	194782.5788	5.425738238	1890414.275	52.75062211
2037	1526116.013	41.66149665	198180.5907	5.388536751	1917255.807	52.20783065
2038	1548546.654	41.31079205	201519.6053	5.352610175	1947103.843	51.77590847
2039	1571990.24	40.993377	204964.0009	5.319862165	1980599.583	51.4410075
2040	1596062.201	40.7123556	208473.3241	5.291058715	2016028.795	51.18372325

Abbreviations: VID, vascular intestinal diseases; ASR, age-standardized rate (per 100,000 population); DALYs, disability-adjusted life years; BAPC, Bayesian age-period-cohort; UI, uncertainty interval.
